# Trabectedin plus CD13-targeted tissue factor tTF-NGR against advanced relapsed or refractory soft tissue sarcoma: translational data, clinical safety and efficacy

**DOI:** 10.1038/s41598-026-40362-4

**Published:** 2026-02-19

**Authors:** Kathrin Hessling, Caroline Brand, Christian Schwöppe, Mirjam Gerwing, Stefanie Pavelka, Andrew F. Berdel, Heike Hintelmann, Rainer Hamacher, Carsten Müller-Tidow, Gerlinde Egerer, Wolfgang Hartmann, Inga Grünewald, Lars H. Lindner, Dorit Di Gioia, Judith S. Hecker, Sabine Maurer, Daniel Pink, Marius Fried, Sergio A. Zapata Bonilla, Anne-Marie Scheuble, Florian Lordick, Philipp Ivanyi, Manfred Fobker, Georg Lenz, Joachim Gerss, Torsten Kessler, Wolfgang E. Berdel, Christoph Schliemann

**Affiliations:** 1 Department of Medicine A, Hematology, Oncology and Pneumology, University Hospital of Muenster, Albert‑Schweitzer‑Campus 1, 48149 Muenster, Germany; 2 Department of Radiology, University Hospital Muenster, Muenster, Germany; 3 Department of Medical Oncology, West German Cancer Center, University Hospital Essen, Essen, Germany; 4 Center for Internal Medicine 5, University Hospital Heidelberg, Heidelberg, Germany; 5 Gerhard-Domagk-Institute for Pathology, University Hospital Muenster, Muenster, Germany; 6 SarKUM, Department of Medicine III, Ludwig Maximilians University Hospital Großhadern, Munich, Germany; 7 Department of Medicine III, School of Medicine and Health and TranslaTUM Center for Translational Cancer Research, Technische Universitaet Muenchen, Munich, Germany; 8 Department of Oncology and Palliative Care, HELIOS Klinikum Bad Saarow, Bad Saarow, Germany; 9 Department of Internal Medicine C, University Hospital Greifswald, Greifswald, Germany; 10 University Cancer Center Mainz (UCT Mainz) and Clinic and Polyclinic for Internal Medicine III, University Hospital Mainz, Mainz, Germany; 11 Department of Medicine (Oncology, Gastroenterology, Hepatology, Pulmonology), University of Leipzig Medical Center, Comprehensive Cancer Center Central Germany (CCCG), Leipzig, Germany; 12 Department of Hematology and Oncology, Medizinische Hochschule Hannover, Hannover, Germany; 13 Central Laboratory, University Hospital Muenster, Muenster, Germany; 14 Institute of Biostatistics and Clinical Research, University of Muenster, Muenster, Germany

**Keywords:** Soft tissue sarcomas, Trabectedin, CD13-targeted tissue factor tTF-NGR, Cancer, Cardiology, Drug discovery, Medical research, Oncology

## Abstract

Trabectedin is standard for r/r soft tissue sarcomas. tTF-NGR accumulates in tumor vasculature leading to tumor vascular occlusion and tumor infarction. Both compounds in sequence could trap trabectedin inside tumors and increase its efficacy, which then optimizes the pro-coagulatory activity of tTF-NGR. This report summarizes translational data and results of the safety run-in patient cohort of the TRABTRAP trial combining trabectedin plus tTF-NGR. A dose of trabectedin of 1.5 mg/m^2^ (24 h, day 1) combined with 1.0 mg/m^2^ of tTF-NGR (1 h, days 2 and 3, q day 22) represents the approx. Maximum tolerated dose (MTD) and with 0.5 mg/m^2^ tTF-NGR (days 2 and 3) the recommended starting dose for the randomized part of TRABTRAP. None of the 6 patients on 0.5 mg/m^2^ tTF-NGR had dose-limiting toxicity (DLT). Higher doses or additional days of application of tTF-NGR led to grade 3 DLT including early troponin T high sensitivity increase, a reversible non-ST-elevation myocardial infarction in one patient, and reversible thromboembolic events. Pharmacokinetics explain the difference of the MTD between the phase I study and in TRABTRAP. Experimental and clinical efficacy and tolerability of the combination between trabectedin and tTF-NGR supports the active randomized part of TRABTRAP.

**Supplementary Information:**

The online version contains supplementary material available at 10.1038/s41598-026-40362-4.

## Introduction

Soft tissue sarcomas (STS) are heterogeneous mesenchymal cancers originating from connective tissue. STS are made up of distinct subtypes, which collectively account for 1% of all adult cancers and the incidence of STS in Europe ranges between 2 and 5/100,000/year^1^. For patients with unresectable advanced STS, chemotherapy is the standard of treatment^2,3^ and the therapeutic approach is multidisciplinary. Overall survival of patients with advanced STS in general remains poor. Thus, there is an unmet medical need for new therapeutic targets and agents in this group of diseases.

Trabectedin, also known as ecteinascidin 743 or ET-743, is an antitumor chemotherapy drug and a DNA minor groove binder. DNA-binding induces single-strand and double-strand breaks and leads to cell cycle arrest and apoptosis^4^. Trabectedin also acts on various molecular events and immune cells within the tumor environment, in particular on tumor mononuclear phagocytes, which is interpreted to contribute to its anticancer effects^5^. The European Medicines Agency (EMA) gave authorization for the marketing of trabectedin for the treatment of patients with advanced soft-tissue sarcoma, after failure of anthracyclines and ifosfamide, or who are unsuited to receive these agents. In the US, on the basis of a trial reported by Demetri^6^, trabectedin is approved for the treatment of liposarcoma and leiomyosarcoma (L-sarcomas) that are either unresectable or have metastasized. Patients must have received prior chemotherapy with an anthracycline or show contraindications.

Since the early reports by Fehleisen and Coley on the activity of tumor necrosis factor (TNF) against sarcomas and the observation by Lejeune and Eggermont of increased antitumor activity by applying higher doses of TNF and other active biomolecules loco-regionally to the tumor^7,8^, attempts are made to target systemically applied biologically active proteins to the sarcoma vasculature. Tissue factor is the initiator of the extrinsic coagulation pathway. It is located mainly on the abluminal side of vascular wall cells such as endothelial cells and pericytes and becomes active upon vascular wall injury. The CD13-targeted tissue factor tTF-NGR is a recombinant fusion protein made of two moieties: tTF (truncated tissue factor) as the main human initiator of extrinsic coagulation lacking the transmembrane domain at the C-terminus, and GNGRAHA as the targeting peptide binding to CD13 fused to the C terminus of tTF instead of the transmembrane domain. CD13 in the vascular system is mainly expressed on the surface of invasive endothelial cells such as in the tumor vasculature. tTF-NGR after binding to CD13 preferentially induces vascular occlusion in the tumor blood vessels leading to tumor infarction and growth retardation of tumor xenograft models of different histology including sarcomas^9^. A phase I study of tTF-NGR in late stage cancer patients has been completed and identified 3 mg/m^2^ of tTF-NGR given as a single compound on 5 consecutive days, q day 22, as being the maximum tolerated dose (MTD). Dose-limiting was an increase of troponin T hs, which was interpreted as early warning sign for hypoxia in myocardial tissues, although it was not specifically mentioned in the CTCAE toxicity criteria^10^.

The rationale for the “trabectedin trap” (TRABTRAP) study was the robust tTF-NGR efficacy against human sarcoma xenografts^11^, high expression of CD13 in human sarcoma vasculature and in sarcoma cells^12^, and the hypothesis to entrap trabectedin selectively in sarcoma tissues (TRABTRAP) by applying the drug and subsequently tTF-NGR, which also leads to increased antitumor efficacy of tTF-NGR, as observed with other cytotoxics^13^.

Here we report non-clinical data supporting this hypothesis and the results of the patient cohort treated with different tTF-NGR doses together with standard doses of trabectedin to establish a safe starting dose for the combination regimen (phase I/II; started 2021 11 12 with observation until 2025 09 27) in the randomized part of the TRABTRAP study (phase III).

## Methods

### Preclinical methodology

For the preclinical methodology the reader is referred to the Suppl. Information.

### Clinical protocol for the safety run-in patient cohort in the TRABTRAP study

The Investigational Medicinal Product (IMP) batches of the fusion protein tTF-NGR used were produced in our Good Manufacturing Production (GMP) facility under the manufacturer´s authorisation number DE_NW_05_MIA_2021_0009/24.05.03-034 and its predecessors. The protein was dissolved in PBS for use. Trabectedin was taken from clinical batches (Yondelis®, Pharmamar, Madrid, Spain) and applied in-label according to standard of care.

The *TRABTRAP study protocol* (EudraCT 2020-005858-21 (date: 2021-09-17), EU CT 2024-516392-33-00 (date: 03/09/2024)) was approved by the Ethics Committee Westphalia-Lippe (AZ. 2021-300-f-A) and the German National Competent Authority (NCA), the Paul Ehrlich Institute (PEI). The study was conducted according to German Arzneimittelgesetz (AMG) and according to the Declaration of Helsinki. Written informed consent was obligatory before a patient could enter the trial. The main part (see protocol in the Suppl. Information) of the study will test trabectedin as standard of care for patients with advanced metastatic soft tissue sarcoma (STS) beyond first line therapy with anthracyclines alone or in combination, or with contraindications to this first line treatment, randomized against identical dosing of trabectedin followed by daily infusions of tTF-NGR with PFS according to iRECIST^14^ as a primary endpoint. Standard inclusion criteria and exclusion criteria were used (see study protocol in Suppl. Information), specifically complemented by requesting CD13 positivity of the sarcoma cells and/or the tumor vasculature. Patients with a history of coronary heart disease, stroke, transient ischemic attacks (TIA), pulmonary embolism, or deep vein thrombosis were excluded with respect to the mechanism of action of tTF-NGR. Clinical suspicion of coronary heart disease had to be further checked e.g. by cardiac MRI or myocardial scintigraphy to exclude coronary heart disease. Patients with known hereditary syndromes with elevated thromboembolic risk (FV Leiden and prothrombin mutations (G20210A), hereditary antithrombin, protein C and S deficiency, and antiphospholipid syndrome) after one or more clinical thromboembolic events, those with hereditary vascular disorders (such as Klippel-Trenauny-Weber syndrome) with increased thromboembolic risk, those with a Khorana score of > 3, and with elevated troponin T hs (> 50 ng/L) before entry on study were also excluded. Further, presence of active central nervous system (CNS) disease and/or CNS vascular abnormalities detected by MRI/CT was a reason for not including patients.

Before the randomized phase III part of the study, a safety cohort of a minimum of 6 patients was performed to obtain at least 2 cycles each of the combination outlined in the combination arm (1.5 mg/m^2^ trabectedin over 24 h plus 3 mg/m^2^ as a starting dose of tTF-NGR given on days 2–4 over 1 h rate-controlled infusion) to confirm safety of the combination. Trabectedin was given as a 24 h infusion since this is the preferred standard regimen for the participating centers. In case of DLT in this safety cohort, a dose-deescalation protocol for tTF-NGR was planned. Dose Limiting Toxicity (DLT) was defined as occurrence of toxicity of Grade 3 or more according to CTCAE version 5.0 for nonhematological toxicity or Grade 4 or more for hematological toxicity. DLT must be considered by the investigator as being causally related to tTF-NGR. Patients were frequently monitored and study specific examinations were performed as outlined in the protocol. The dose at which 6 patients tolerated 2 cycles without DLT was chosen for the randomized part of the study. This report describes the results of this safety run-in cohort of the TRABTRAP study. This safety cohort had characteristics of a phase I and early phase II study.

### Contrast-enhanced dynamic magnetic resonance imaging (MRI)

Contrast-enhanced dynamic multiparametric MRI was performed exactly according to protocols published before^10,15,16^. Ferrocarbotran and gadobutrol were applied as contrast agents before and after therapy as indicated. Treatment effects were assessed by the following parameters: vascular volume fraction (VVF) was measured for the intratumoral blood volume, k_trans_ (min^−1^) as an established model-based perfusion parameter, and apparent diffusion coefficient (ADC) in mm^2^/s as a quantification of the mobility of water molecules that was shown to correlate with treatment response.

### Statistical analysis

PFS and OS were estimated with the Kaplan–Meier Method and are defined as in the protocols attached (Suppl. Information).

## Results

### Preclinical and translational data leading to the study hypothesis

**Fig. 1:**
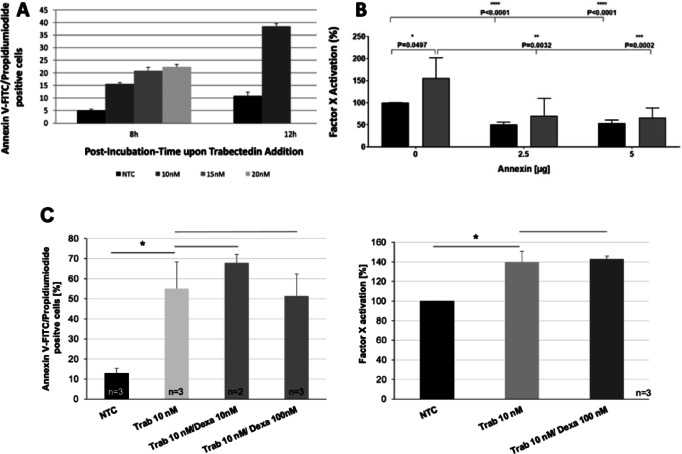
Trabectedin increases the PS concentration on the outer membrane of HUVEC and subsequently increases the pro-coagulatory potential of tTF-NGR. (**A**) Evaluation of 7 different experiments with different doses of trabectedin inducing PS upregulation on HUVEC after 8 and after 12 h by increasing doses of trabectedin (*p* values for all time points < 0.0001 when compared to the non-trabectedin control (NTC)). Propidiumiodide was used as an internal necrosis control. **(B)** Trabectedin-dependent increase of pro-coagulatory activity of HUVEC upon binding of tTF-NGR (black, without trabectedin, grey, with trabectedin (10 nM, 8 h)) and complete abrogation of this effect by masking of PS with annexin V at different doses. Means of 3 experiments with at least fourfold assays each + standard error and *p* values. (**C**) In different experimental settings HUVEC were treated with trabectedin (3–18 h) and dexamethasone (18–45 h) in sequence. Evaluation of the pro-apoptotic status of the cells by Annexin V-/Propidiumiodide-FACS-staining revealed trabectedin-dependent increase of PS on the outer leaflet of the phospholipid bilayer of the HUVEC cellular membrane and thus an increase of the pro-apoptotic status of HUVEC cells (light grey), but showed no effect of dexamethasone on this increased pro-apoptotic status of HUVEC (left panel, dark grey; t-test: NTC vs. Trab 10 nM: *p* = 0.0344*; Trab 10 nM vs. Trab 10 nM/Dexa 10 nM: *p* = 0.516; Trab 10 nM vs. Trab 10 nM/Dexa 100 nM: *p* = 0.838). In the right panel (NTC set at 100%) identically treated cells were analyzed for Factor X activation: trabectedin treatment increased the tTF-NGR-dependent pro-coagulatory activity of HUVEC (light grey) whereas sequential addition of dexamethasone had no influence on this pro-coagulatory activity (dark grey; n = 3 with 3–4 technical repeats each; t-test: NTC vs. Trab 10 nM: *p* = 0.0226*; Trab 10 nM vs. Trab 10 nM/Dexa 100 nM: *p* = 0.798).

We had shown that combination of tTF-NGR with doxorubicin showed better therapeutic activity in vivo when applied in the sequence of doxorubicin before tTF-NGR than each of the compounds as monotherapy with complete abrogation of tumor growth in the animals treated for prolonged times of observation and had provided mechanistical data to explain this combinatorial efficacy^13^. To assure this effect for tTF-NGR and trabectedin and the mechanistic basis for this we tested the pro-apoptotic activity of trabectedin on Human Umbilical Vein Endothelial cells (HUVEC) and also human HT1080 sarcoma cells. Trabectedin significantly increased phosphatidylserine (PS) presence on the outer leaflet of the phospholipid bilayer building the cell membrane (Fig. 1A for HUVEC), and by this the resulting optimized phospholipid milieu in the outer cellular membrane potentiated the pro-coagulatory efficacy of tTF-NGR within the Factor VIIa:tTF-NGR:CD13 complex on the cell membrane (Fig. 1B). This effect was specifically dependent on the presence of PS as it was completely abolished by masking PS via preincubation with annexin V, a PS binder (Fig. 1B), but not by other substances such as corticosteroids (Fig. 1C).

**Fig. 2:**
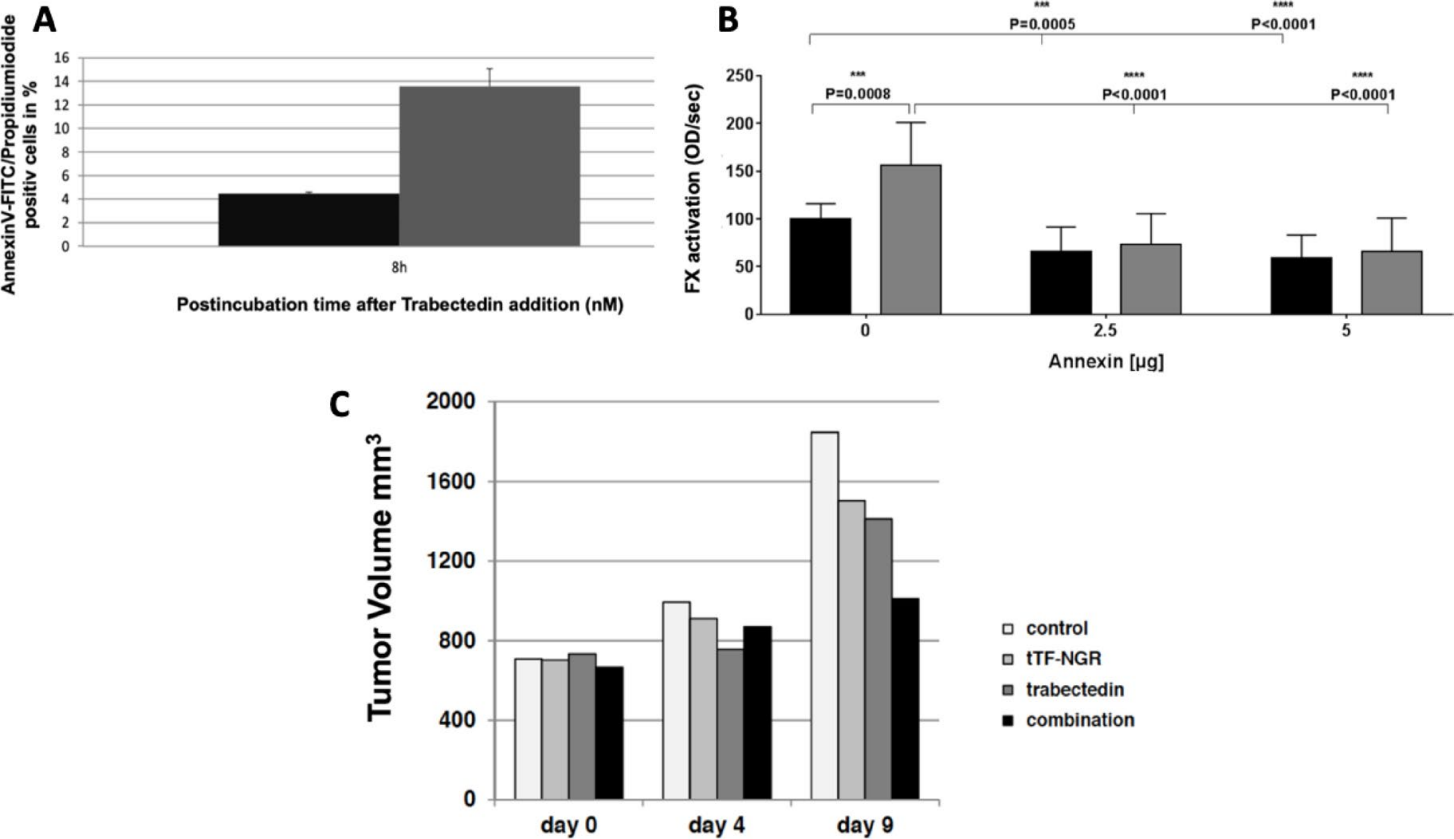
Effects of trabectedin and tTF-NGR on human HT1080 sarcoma in vitro and in vivo. (**A**) Evaluation of 6 different experiments with 10 nM of trabectedin (grey) and 8 h incubation time showing significant PS upregulation on HT1080 sarcoma cells when compared to the non-trabectedin control (black). Means + standard error (*p* value = 0.001). Propidiumiodide was used as an internal necrosis control. (**B**) Trabectedin-dependent increase of pro-coagulatory activity of HT1080 sarcoma upon binding of tTF-NGR (black, without trabectedin, grey, with trabectedin (10 nM, 8 h)) and complete abrogation of this effect by masking of PS with annexin V at different doses. Means of 4 experiments + standard error and *p* values. (**C**) Therapeutic results in vivo in a HT1080 STS xenograft model (single experiment according to the 3R rule). Control, PBS iv; tTF-NGR 1 mg/kg iv day 0; trabectedin 0.1 mg/kg iv day 0; combination, trabectedin plus tTF-NGR (5 h after trabectedin) with identical doses and time schedule (control n = 8, tTF-NGR n = 9, trabectedin n = 9, combination n = 11; at start of experiment). The experiment had to be terminated after measurement on day 9 as dictated by animal protection legislation. Significance (unpaired two-sided t-test) comparing tumor volume increase of PBS control versus combination *p* = 0.019, comparing PBS versus trabectedin *p* = 0.159, comparing PBS versus tTF-NGR *p* = 0.090.

Similar results on upregulation of PS by trabectedin and subsequent increase of pro-coagulatory tTF-NGR efficacy were observed with HT1080 human sarcoma cells (Fig. 2A,B). Further, a systemic combination treatment of trabectedin followed by tTF-NGR inhibited in vivo HT1080 xenograft growth to a higher extent than both compounds when given individually (Fig. 2C). In contrast to the experiments with combinations of doxorubicin and tTF-NGR^13^, we could not reliably test comparative intratumoral trabectedin concentrations upon application in combination with tTF-NGR to mice carrying human sarcoma HT1080 xenografts, since the explanted tumors were completely lytic and sticky after the combination treatment.

### Clinical safety of the TRABTRAP combination in patients with advanced and refractory soft tissue sarcomas

Twenty-six patients entered the dose finding safety cohort of the TRABTRAP trial, 7 of those were screening failures, all due to existing exclusion criteria as defined by the protocol (Supporting Information). We have treated 19 patients within this safety cohort, with 11 out of those having obtained more than one preceding line of systemic therapy prior to entry on trial. Trabectedin dose and application was according to standard of care (1.5 mg/m^2^ over 24 h, day 1, q day 22), tTF-NGR starting dose was 3 mg/m^2^ given as a rate-controlled 1-h infusion on days 2–4, q day 22. Treatment delay or dose reduction of trabectedin was according to standard of care (SoC) as outlined in the SmPC. A de-escalation schedule for tTF-NGR was followed after DLT occurred (see Study Protocol, Supporting Information). Table 1 summarizes the characteristics of all patients treated. A complete AE/SAE overview is given in the Suppl. Table 1. Trabectedin resulted in a noticeable increase of serum transaminases up to grade 4 as known for the drug and also in hematotoxicity. This was completely reversible in all patients. For two patients at the tTF-NGR dose levels of 2 mg/m^2^ and at 1 mg/m^2^, we have interrupted tTF-NGR in the first cycle for 1 day to observe possible additive liver toxicity, but further experience showed reversible transaminase levels under continued tTF-NGR, and lower degrees of increase in follow-up cycles as known for trabectedin, so that we did not pause tTF-NGR anymore when observing trabectedin-related transaminase increase.

**Table 1:** Characteristics and outcome of the 19 patients treated in the safety run-in cohort of the TRABTRAP study.

Gender (m/f)/age subject number	Diagnosis previous Tx, CD13 status	tTF-NGR dose level (mg/m^2^ × days). No. of cycles (started)	DLT	Outcome safety	Outcome efficacy
m/21 1	Synovial sarcoma, G3, UICC stage IV, surgery, ifo/doxo, CD13: v = 1+; t = 2+, c = 2+	3.0 × 4 2	SUSAR/DLT: segmental pulmonary embolus after cycle 2, asymptomatic, grade 3 (CTCAE)	Resolved recovered in next CT scan	iUPD with 1 unconfirmed new lesion and shrinkage of a target lesion
m/59 2	Leiomyosarcoma, G3, UICC stage IV, surgery, radiation, doxo, CD13: v = 2+, t = 0, c = 2+	2.0 × 4 2	SUSAR/DLT: asymptomatic N-STEMI under tTF-NGR combination, but again after trabectedin monotherapy, both grade 3	Resolved recovered both times (see case summary)	SD with tumor lesion shrinkage of − 10%
f/61 3	Leiomyosarcoma, G3, UICC stage IV, ifo/doxo, surgery, radiation, CD13: v = 3+, t = 3+, c = 3+	1.5 × 4 1	SAR/DLT: increase of troponin T hs in cycle 1 without further sequelae, grade 3 (?)	Resolved recovered	SD with tumor lesion shrinkage of − 8%
m/65 4	Liposarcoma, G3, UICC stage IIIB, surgery, ifo/doxo, CD13: v = 2+, t = 1+, c = 2+	1.0 × 4 2	None	SAE: slow troponin T hs increase not reasonably related to tTF-NGR but related to trabectedin, no DLT. Resolving recovering	iUPD
m/51 5	Synovial sarcoma, G3, UICC stage IV, ifo/doxo, surgery, gemcitabine/docetaxel, pazopanib, cyclophosphamide/fludarabine/autologous T-cells (NY-ESO targeted), CD13: v = 2+, t = 2+, c = 2+	1.0 × 4 3	None	No SUSAR or SAE	iCPD
m/52 6	Liposarcoma, G3 UICC stage IV, ifo/doxo, eribulin, CD13: v = 2+, t = 1+, c = 2+	1.0 × 4 3	None	SAR after 3 cycles: PORT related thrombus, CTC AE grade 2 Resolving/recovering under apixaban	SD with tumor lesion shrinkage of − 6% after 3 cycles SD with tumor lesion shrinkage of − 14% after 4 further cycles with trabectedin mono
f/40 7	Leiomyosarcoma, G3 UICC stage IV, doxo, pazopanib, CD13: v = 2+, t = 0, c = 2+	1.0 × 4 5	None	No SUSAR SAE after 5 cycles due to PD	SD with mean tumor lesion shrinkage of − 25% after 3 cycles and PD after 5 cycles
m/53 8	Leiomyosarcoma UICC stage IV, multiple surgeries and lung metastasectomies, radiotherapy, doxo + olaratumab, gemcitabine/docetaxel L19-TNF + dacarbazine, CD13: v = 2+, t = 1+, c = 2+	1.0 × 4 2	SUSAR grade 3, DLT in cycle 2	SUSAR grade 3, DLT in cycle 2: troponin T hs increase, symptomatic segmental thrombo-embolic event in lower lobe of right lung in a previously operated area with asymptomatic deep vein thrombosis Resolved/recovered under anticoagulation	PD
m/44 9	MPSNT metastatic, surgery, radiotherapy, gemcitabine/docetaxel, ifo/carboplatin/etoposide, trofosfamide, CD13: v = 2+, t = 2+, c = 2+	1.0 × 2 3	None	No SUSAR or SAE	iUPD after 3 cycles RECIST: no new lesions, target lesion increase + 27.9%
f/38 10	Synovial sarcoma, G3, UICC stage IV, surgery, radiotherapy, ifo/doxo, high-dose ifo, CD13: v = 0, t = 3+, c = 3+	1.0 × 2 2	None	No SUSAR, DLT SAE due to early tumor progression in cycle 2	iCPD in imaging and clinical symptoms in cycle 2
m/34 11	Myxoid liposarcoma, G3, UICC stage IV, radiotherapy, surgery, ifo/doxo, CD13: v = 2+, t = 3+, c = 3+	1.0 × 2 12	None	No SUSAR Following cycles postponed due to trabectedin- related thrombocytopenia	SD with mean tumor lesion shrinkage of − 4.3% after 9 weeks, continuing stable disease after > 52 weeks
f/30 12	Synovial sarcoma, G2 UICC stage IV, surgery, radiotherapy, ifo/doxo, pazopanib, CD13: v = 2+, t = 0, c = 2+	1.0 × 2 6	None	No SUSAR. SAE due to elevated ASAT + ALAT related to trabectedin resolving/recovering, and suspected port tip thrombus grade 2	PR with − 71% tumor lesion shrinkage after 9 and 18 weeks
m/60 13	Leiomyosarcoma, G3 UICC stage IV, surgery, doxorubicin, gemcitabine/dacarbazin, CD13: v = 2+, t = 3+, c = 3+	1.0 × 2 1	SUSAR grade 3, DLT in cycle 1 (subsegmental pulmonary embolism)	Early tumor progression in cycle 1 Subsegmental pulmonary embolism, resolved/recovered	iUPD
f/35 14	Synovial sarcoma, G3, UICC stage IV, surgery, ifo/doxo, gemcitabine/docetaxel, pazopanib, CD13: v = 3+, t = 1+, c = 3+	0.5 × 2 3	None	No SUSAR or SAE	iUPD
f/53 15	Leiomyosarcoma, G3, UICC IV, surgery, ifo/doxo, ifo/doxo + regional hyperthermia, CD13: v = 2+, t = 3+, c = 3+	0.5 × 2 6	None	SAE/SUSAR: grade 2 anemia in cycles 3, 4, and 6 related to tTF-NGR and to trabectedin Recovered	iUPD after 9 weeks (+ 32.5%) iPD after 18 weeks
f/53 16	Uterine leiomyosarcoma, G2, UICC IV (lung), doxo/dacarbazin, surgery, CD13: v = 2+, t = 3+, c = 3+	0.5 × 2 14	None	No SUSAR or SAE	SD with mean tumor lesion shrinkage of − 4.2% after 9 weeks (3 cycles), − 8.4% after 27 weeks (8 cycles), and -24.2% after 36 weeks iPD from nadir after 45 weeks
f/64 17	Leiomyosarcoma, G3 UICC IV, surgery, doxo, gemcitabine/docetaxel, CD13: v = 2+, t = 0, c = 2+	0.5 × 2 14	None	SAE related to trabectedin and not to tTF-NGR. Cardiomyopathy (see case report) Recovering/resolving	PR after 9 weeks with tumor shrinkage of − 35% and − 47.4% continuing after 18, 27, 36, and 45 weeks. Then End of Treatment with tTF-NGR due to anticoagulation for a deep vein thrombosis of grade 2 and continuing trabectedin monotherapy
f/62 18	Myxoid sarcoma (NOS), G2, UICC IV, surgery, radiotherapy, ifo/doxo + regional hyperthermia, CD13: v = 1+, t = 0, c = 1+	0.5 × 2 12	None	No SUSAR. SAE as jugular thrombosis grade 2 before cycle 13 at restaging (week 36), still being in SD	SD after 9, 18, 27 and 36 weeks with mean target lesion shinkage of − 12.9% End of Treatment due to anticoagulation beginning at week 36
m/58 19	Fibrosarcoma, G2 UICC IV, surgery, radiotherapy, ifo/doxo CD13: v = 2+, t = 2+, c = 2+	05. × 2 2	None	SAE neutropenia and thrombocytopenia grade 4 related to trabectedin and not related to tTF-NGR, recovering/resolving. SAE grade 2 as reaction to platelet transfusion, recovered/resolved. Progressive disease after cycle 2	iPD

m, male; f, female; CD13: v, vascular score, t, tumor cell score, c, composite score according to^12^; DLT, dose limiting toxicity; doxo/ifo, doxorubicin/ifosfamide; iCPD, i confirmed progressive disease according to iRECIST^14^; iUPD, i unconfirmed progressive disease according to iRECIST; iSD, i stable disease according to iRECIST after 3 cycles if not stated otherwise; SAE, severe adverse event; SAR, severe adverse reaction; SUSAR, suspected unexpected severe adverse reaction; N-STEMI, non-ST elevation myocardial infarction; L19-TNF, TNF (tumor necrosis factor) coupled to an antibody against the EDB domain of fibronectin; MPSNT, malignant peripheral nerve sheath tumor. Patient numbers are double-pseudonymized. Outcome is documented for the time of treatment. Overall survival is described in the case reports (Suppl. Information, Appendix A, date 2025 09 27).

**Fig. 3:**
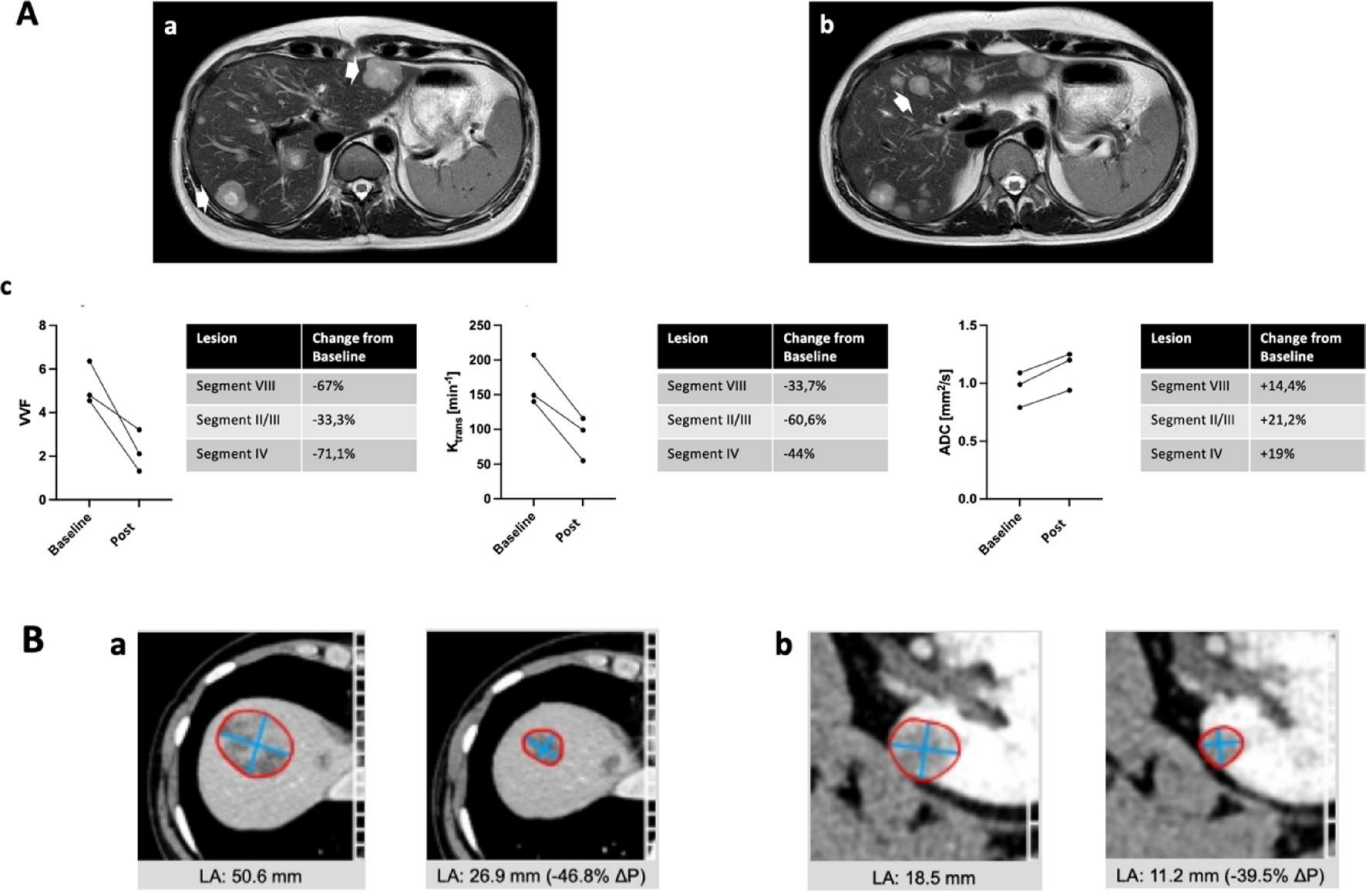
Multiparametric MRI and iRECIST of a patient (subject number 7) obtaining the combination with 1 mg/m^2^ tTF-NGR. (**A**) (**a**,**b**) T2w images of a leimyosarcoma with multiple liver metastases. White arrows indicate the analyzed lesions. (**c**) Analysis of the vascular volume fraction (VVF), k_trans_ from dynamic contrast enhanced (DCE)-imaging and diffusion weighted imaging (apparent diffusion coefficient, ADC) of the three independently analyzed lesions show a significant decrease in perfusion and permeability (VVD and k_trans_) as well as an increase in ADC. All 3 lesions were analyzed separately. Baseline, before treatment onset of cycle 2; Post, 5 h after the 2nd application of tTF-NGR (day 3, cycle 2). (**B**) CT scans. A target lesion liver, segment VIII. (**a**) Left: before start of cycle 1, right: after cycle 3. A 2nd lesion showed no change, thus the overall result was SD. (**b**) target lesion left kidney. Left: before start of cycle 1, right: after cycle 3.

Appendix A (Suppl. Information) contains summarized case reports of all 19 patients treated focusing on safety aspects for the patients sorted by tTF-NGR dose level. The MTD of tTF-NGR in the TRABTRAP combination was considerably lower than in the phase I monotherapy and approx. at the 1 mg/m^2^ dose level. To test remaining “target hit” for this dose level of 1.0 mg/m^2^ tTF-NGR, we measured selective inhibition of tumor perfusion in a patient (leiomyosarcoma, f/40, patient number 7 in Table 1) on this dose level. A dynamic multiparameter magnetic resonance imaging (MRI) protocol as published in the phase I report^10,15,16^ was repeated and imaging confirmed selective inhibition of tumor blood perfusion as observed before (Fig. 3A). Figure 3 further shows the size reduction of target liver and kidney lesions after 3 cycles in this patient (Fig. 3B), counted as a RECIST stable disease since a 3rd lesion showed less shrinkage (for further details of this case report see Suppl. Information).

At the dose level of 0.5 mg/m^2^ of tTF-NGR given at days 2 and 3 no DLT was observed in 6 patients treated with more than 2 cycles and up to 14 cycles, thus the objective of the safety run-in cohort was reached.

**Fig. 4:**
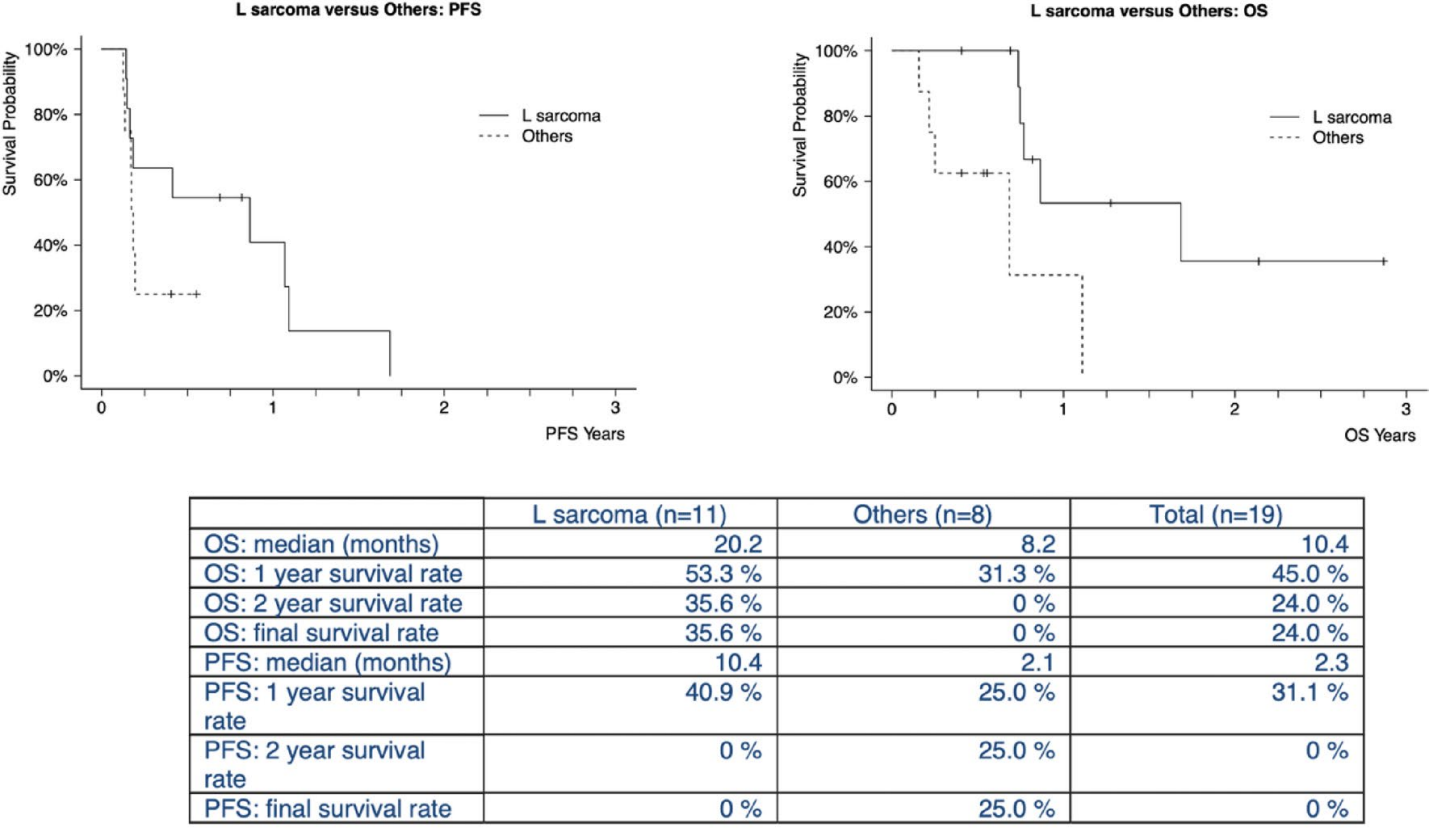
Survival parameters for the 19 safety run-in cohort patients. PFS, progression free survival. OS, overall survival. L sarcoma, liposarcoma and leiomyosarcoma. Others, for histology see Table 1.

All 19 patients who received at least one dose of tTF-NGR in the combination regimen have reached TP1 restaging and were included in the efficacy analysis. Ten out of 19 had (iU)PD, 7 (6 L-sarcomas, 1 myxoid sarcoma) had SD with measurable shrinkage of target lesions in all of them, and 1 synovial sarcoma and 1 leiomyosarcoma, respectively, showed PR. Further, 7 out of 11 patients treated with L sarcomas had SD or PR with measurable tumor shrinkage in the iRECIST evaluation after 9 weeks (see Table 1), giving a disease control rate (DCR) of 63.6% for L sarcomas. Interestingly, patients treated with 1 mg/m^2^ tTF-NGR or lower obtained a higher number of combination cycles with some holding SD after even more than 1 year. At the time of this report 10 out of 19 patients had died, all due to progressive disease. Figure 4 depicts survival parameters of the 19 patients.

### Laboratory examinations accompanying the safety run-in cohort

**Fig. 5:**
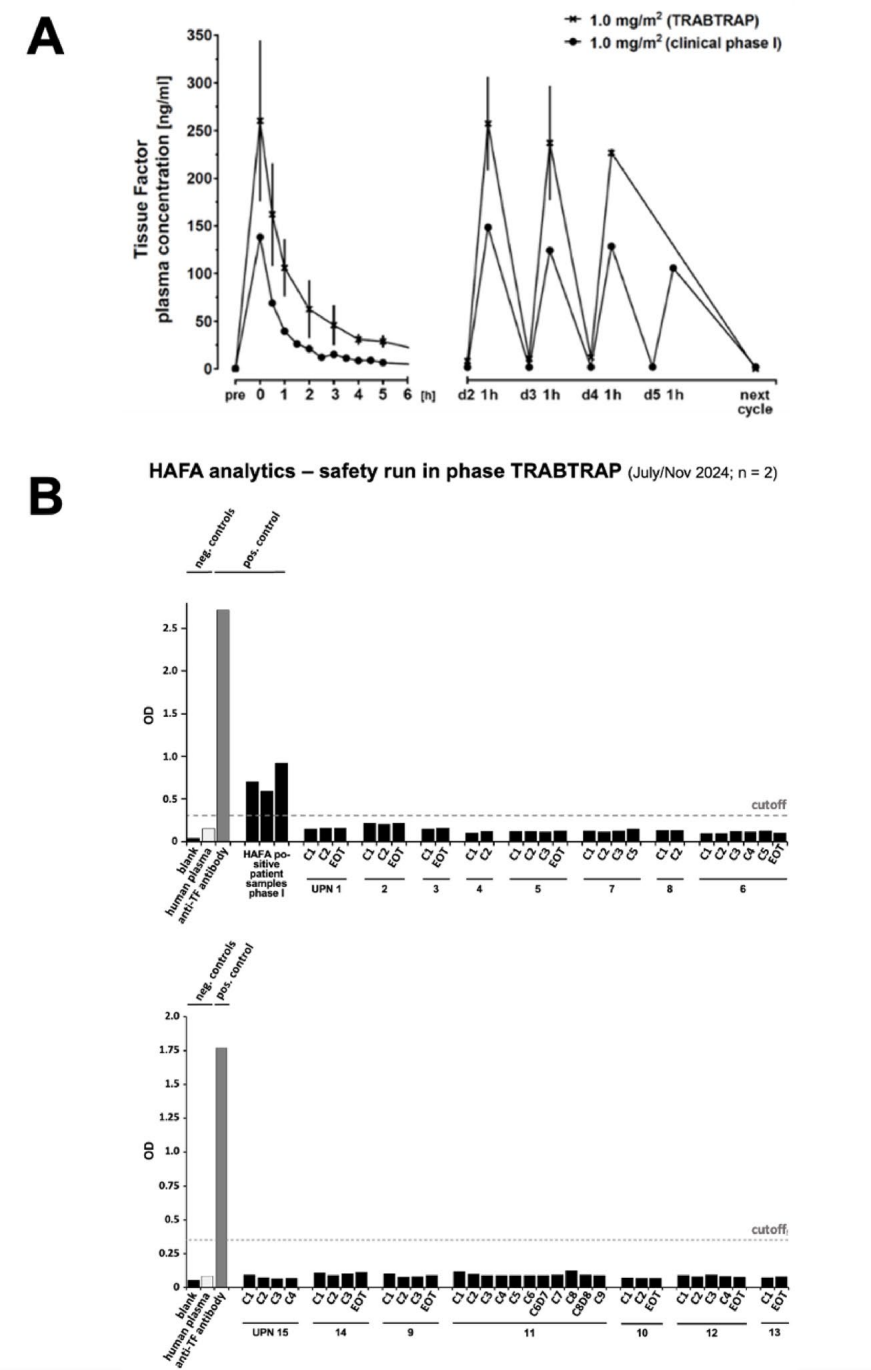
Pharmacokinetics (PK) and HAFA antibody formation. (**A**) PK with TF-plasma concentration–time curves for 1 mg/m^2^ tTF-NGR when given in combination with trabectedin for up to 3 cycles (TRABTRAP (n = 9); day 1: C_max_ = 260.21 ng/mL, AUC = 895.43 ng/h*mL; for details, see Suppl. Table 2), or when given as monotherapy (clinical phase I (n = 1); day 1: C_max_ = 138.10 ng/mL, AUC = 285.54 ng/h*mL; see^10^, respectively. Error bars of the TRABTRAP cohort indicate standard deviations. (**B**) Detection of human anti-fusion protein (tTF-NGR) IgG antibodies (HAFA) in the patients after trabectedin-tTF-NGR combination therapy (TRABTRAP) in comparison to plasma from patients treated by tTF-NGR monotherapy (updated from phase I^10^ assayed as controls (upper figure)). Patients are listed with arbitrary Unique Patient Numbers (UPN) identical with Table 1; results of two different test sets (above and below) are shown (each n = 2). HAFA-positive patient samples were only used once in the first test set (above); *OD* optical density, *C* cycle, *D* day, *EOT* end of treatment (for details see Supporting Information).

We have evaluated the *pharmacokinetics* (PK) of tTF-NGR in the patients treated within this safety cohort and compared the results with the PK studies of the preceding phase I monotherapy trial. Figure 5 shows one representative example at the dose level of 1 mg/m^2^ tTF-NGR. Considerably higher Cmax values at 1 mg/m^2^ and AUC values at every dose level tested were observed for tTF-NGR when given after trabectedin as compared to single application. Thus, the comparison of the global values for t1/2_alpha_, t1/2_term_ and the comparison of the elimination constant k_el_ indicated considerably delayed elimination of tTF-NGR when given after trabectedin (Suppl. Tables 2–5).

In contrast to the results obtained in phase I after tTF-NGR monotherapy, none of the 16 patients tested so far in TRABTRAP developed *human anti-tTF-NGR antibodies (HAFA)* within an observation period of up to 9 cycles when compared with the positive controls (anti-TF antibody) and HAFA-positive patient samples from phase I monotherapy, respectively (Fig. 5B). Possible interference of trabectedin with B-cells and antibody production should be further studied.

To obtain further information on hypothetical long-term presence of active tTF-NGR within CD13-positive tissues, beyond standard blood pharmacokinetics, we have tested for cellular internalization of the tTF-NGR:CD13 complex after binding by CD13-positive endothelial cells. This would ultimately destroy tTF-NGR e.g. by intracellular proteasomes. Approx. 58% of the tTF-NGR:CD13 complex was internalized by untreated HUVEC after 3 h of incubation (Suppl. Fig. 1). After preincubation of HUVECs with trabectedin, the presence of CD13 on HUVECs was only slightly reduced (Suppl. Fig. 2), but there was measurable reduction of binding of tTF-NGR to CD13, and internalization of the complex tTF-NGR:CD13 into HUVECs was considerably reduced upon the in vitro combination of trabectedin incubation followed by tTF-NGR (Suppl. Figure 1D). Thus, fast and effective cellular internalization of the tTF-NGR:CD13 complex upon binding to CD13-positive cells contributes to the disappearance of the molecule in CD13-positive tissues, when tTF-NGR is given alone. In contrast, this way of elimination of the molecule is delayed by trabectedin which explains longer presence of the tTF-NGR molecule in the circulation after application of the combination TRABTRAP protocol. Further, as half-life of tTF-NGR was prolonged by previous application of trabectedin to the patients, we observed a slight accumulation of tTF-NGR in the combination protocol within one cycle in contrast to the monotherapy phase I (Suppl. Fig. 3).

Finally, we compared functionality of tTF-NGR in the plasma of patients from phase I versus TRABTRAP (Suppl. Fig. 4). On the basis of identical protein amounts and without influence of confounders such as storage, the ability of Factor VIIa:tTF-NGR to activate Factor X to Xa was higher when trabectedin was given to the patients before. As this was dependent from time in plasma this indicated a higher degree of molecular integrity of tTF-NGR, possibly due to decreased liver protease levels after trabectedin, to lack of functional blockage by neutralizing antibodies (HAFA), and/or release of endogenous TF from endothelial cells damaged by trabectedin.

This together results in longer presence of tTF-NGR in the blood circulation when given after trabectedin (overview in Suppl. Fig. 5).

## Discussion

Pre-clinical experiments supported a combinatorial therapeutic anti-tumor effect for a sequence of trabectedin application followed by tTF-NGR, as reported previously with another organic cytotoxic compound doxorubicin^13^. One mechanistic explanation is the enrichment of PS in the outer leaflet of the cellular membrane of growing endothelial cells, such as in the tumor vasculature, by the pro-apoptotic effect of trabectedin as with doxorubicin on those cells and the subsequent increase of pro-coagulatory effects by tTF-NGR. These pro-coagulatory effects are caused by the cell-bound FVIIa:tTF-NGR:CD13 complex activating FX to FXa and inducing the extrinsic coagulation pathway. The tumor-entrapping effect for cytotxic compounds by vascular occlusion induced by tTF-NGR subsequently increasing the tumor exposure to trabectedin and its anti-tumor efficacy vice versa, as observed for a sequential doxorubicin-tTF-NGR application^13^, could not be exactly proven since all methods to measure intratumoral trabectedin concentrations failed in the lytic tumor material obtained from the animals treated with the combination and the experiment could not be repeated due to animal protection legislation. However, the entrapment mechanism by vascular occlusion should be similar for all organic cytotoxics. Giving a sequence of both drugs increased the therapeutic efficacy against STS xenografts. Trabectedin was chosen as a combination partner for tTF-NGR in this study since it has less cardiotoxicity than doxorubicin and since tTF-NGR had a cardiovascular risk profile in phase I.

The observed combinatorial efficacy is the rationale for the TRABTRAP trial currently active in 11 German sarcoma centers. The safety cohort of TRABTRAP presented here had the main objective to establish a safe dose of the combination. Dose levels higher than 1.0 mg/m^2^ of tTF-NGR consistently led to dose-limiting toxicity (DLT) always in the first patient treated. To this end, the parallel occurrence of a steep increase of troponin T hs shortly after the application of tTF-NGR together with terminal T-wave inversion in V3–V4 of the ECG in the patient on the 2 mg/m^2^ dose level, interpreted as N-STEMI although without symptoms or further functional or imaging signs of myocardial infarction, retrospectively supports steep and early troponin T hs increase as being an important biological safety marker. CTCAE 5.0 and also previous versions of CTCAE do not mention high sensitivity troponin assays. On the other hand, increase of high sensitivity troponin upon application of anti-cancer drugs belonging to multiple classes, such as anthracyclines, trabectedin, trastuzumab, sunitinib, and nivolumab, is frequently observed but usually just registered and not interpreted as DLT^17–21^. In particular, slow increase of troponin T hs beyond the short half-life of tTF-NGR may indicate non-vascular myocardial damage as seen in some cases under different anticancer drugs such as doxorubicin or trabectedin. The DLTs led to a further stepwise reduction of tTF-NGR dose. Five patients could be treated on a dose level of 1 mg/m^2^ × 4, q day 22 of tTF-NGR for > 2 cycles without DLT. The 5th patient on this dose level had a DLT in cycle 2 which resolved. Subsequently, further 5 patients have been treated for > 2 cycles on a dose level of 1 mg/m^2^ × 2, q day 22 of tTF-NGR without DLT. Again, the 5th patient on this dose level had a DLT as a subsegmental pulmonary embolism. When evaluating safety in all 10 patients treated on the dose level of 1 mg/m^2^ given either for 4 or for 2 days, this with a 20% rate of DLTs is the approximate maximum tolerated dose (MTD) for tTF-NGR when given after trabectedin. Remarkably, this level is only one third of the MTD for tTF-NGR in phase I, which was 3 mg/m^2^ allowing for 1 DLT in 6 patients treated^10^. No grade 4 or grade 5 toxicity was observed in the patients treated.

The protocol requested a DLT percentage of ≤ 10% for safety reasons before randomization could start (63 patients planned in the combination arm). Thus, subsequently, 6 patients were treated on a dose level of 0.5 mg/m^2^ tTF-NGR given at days 2 and 3 of a cycle. Since no DLT occurred, we estimated the chance to observe DLT in the randomized phase of TRABTRAP as being ≤ 10% and recommend to start randomization on this starting dose level.

The occurrence of 7 patients with venous thromboembolic events (VTE: 3 small pulmonary embolisms, of whom 1 also had a DVT in the leg, 1 DVT after cycle 14 in continuing PR, and 3 asymptomatic Port-associated thrombi with 1 of those only suspected) occurring in 19 patients treated in this safety cohort, with respect to the short observation interval of the study, is higher than background data reported for sarcoma patients^22–25^. However, none of these events was life-threatening, only 3 were symptomatic, and all of them recovered/resolved after stop of tTF-NGR and anticoagulation. It should be further emphasized that all DLTs observed at higher doses occurred early and that the fact that several patients at the dose levels of 1 and 0.5 mg/m^2^ tTF-NGR continued to be treated for almost 1 year, with due caution argues against a relevant risk of additional chronic toxicity during long term application of this regimen.

The explanations why 1 mg/m^2^, i.e. 1/3 of the single drug MTD (3 mg/m^2^ tTF-NGR) is the approx. MTD level of this combination are based on striking differences in the pharmacokinetics of tTF-NGR when monotherapy is compared with the trabectedin combination. All PK studies characterizing the elimination of tTF-NGR (K_el_, t1/2, cellular internalization) showed delayed elimination of tTF-NGR. The terminal half-life of 1 mg/m^2^ tTF-NGR after trabectedin equals the terminal half-life of 3 mg/m^2^ tTF-NGR in the monotherapy. How can trabectedin lead to this altered PK? Besides binding to other molecules, proteins of an approx. size of 30 kDa are eliminated via the kidneys, by proteases, or if designed as tTF-NGR also by internalization into CD13-positive cells. The renal elimination of tTF-NGR was not measured in this part of the study, but trabectedin renal toxicity was not impressive. In contrast, trabectedin showed liver toxicity as known before, and with increase of transaminases we also observed decrease of liver synthesis parameters under trabectedin such as total protein, albumin and plasma cholinesterase (PCHE; often below 50% of the lower value normal = 5320 U/L), some of these values even roughly correlating with PK values. This could lead to changes in functional integrity of the molecule, which is supported by our experiments showing higher pro-coagulatory activity of tTF-NGR from plasma of TRABTRAP patients versus from phase I patients on the basis of identical protein amounts detected by the ELISA. Another reason for this observation could be the lack of HAFA observed in the TRABTRAP combination. This observation in addition may indicate an interference of trabectedin with B-cells and antibody production and should be further studied as it might have broader implications for immunogenicity in protein-based combination therapies. Further, in vitro experiments show inhibition of binding and internalization of the tTF-NGR:CD13 complex by endothelial cells by preincubation of the cells with trabectedin. Experiments with growing endothelial cells suggest, that trabectedin itself increases PS on the endothelial cell surface of areas with vascular remodeling also outside a tumor vasculature which could increase systemic pro-coagulatory activity of tTF-NGR. In addition, trabectedin was described as upregulating the production of multifunctional thrombospondin-1 in cell lines^26^ which also could play a role. On the other hand, inhibition of tumor vascular perfusion upon 1 mg/m^2^ of tTF-NGR was more pronounced than under the same dose given as monotherapy^10,15,16^. Thus, selective action of tTF-NGR on tumor endothelial cells is demasked only at lower dose when such a combination is applied and the dose used in the randomized phase of the trial bears no general disadvantages.

A standard dose of trabectedin of 1.5 mg/m^2^ given over 24 h on day 1 and dose-adjusted according to standard of care (SmPC and institutional guidelines), combined with 0.5 mg/m^2^ of tTF-NGR given over 1 h on days 2 and 3, q day 22, as a starting dose, with some further adjustment according to protocol for > 2 cycles is safely applicable to patients. This is chosen as starting combination dose for the randomized phase III part of the TRABTRAP study.

Limitations of this study concentrate on efficacy. With the small number of only 19 patients (11 L-sarcomas, 8 non L-sarcomas) treated within this cohort and safety being the main objective of the run-in cohort, anti-sarcoma efficacy of the combination of trabectedin and tTF-NGR cannot yet be judged. However, preliminary efficacy analysis of this combination in the patient cohort which was heavily pretreated and late stage, supports further evaluation in the randomized part of the trial.

The results reported recommend the conduct of the randomized phase III part of the TRABTRAP trial. Results show manageable toxicity of the combination of trabectedin and tTF-NGR and preliminary signs of efficacy also within the recommended dose level in an advanced malignant disease relapsed after or refractory to preceding lines of therapy. The randomized part of the TRABTRAP study continues enrollment.

## Supplementary Information

Below is the link to the electronic supplementary material.

Supplementary Material 1

## Data Availability

All data are presented in the manuscript and the Supplementary Information.
