# Supplementary material for: Trabectedin plus CD13-targeted tissue factor tTF-NGR against advanced relapsed or refractory soft tissue sarcoma: translational data, clinical safety and efficacy
Source: Sci Rep. 2026 Feb 19;16:7389. doi: 10.1038/s41598-026-40362-4 (PMC12923852; doi:10.1038/s41598-026-40362-4)
Supplement: Supplementary file 1 — Supplementary Material 1 [file 41598_2026_40362_MOESM1_ESM.pdf]

APPENDIX A

SUPPORTING INFORMATION

Trabectedin plus CD13-targeted Tissue Factor tTF-NGR Against Advanced Relapsed or Refractory Soft Tissue Sarcoma: Translational Data, Clinical Safety and Efficacy

Hessling K, Brand C, et al. 2025

Supplementary Materials

Preclinical and translational materials and methods

tTF-NGR and trabectedin

The Investigational Medicinal Product (IMP) batches of the fusion protein tTF-NGR used were produced in our Good Manufacturing Production (GMP) facility under the manufacturer´s authorisation number DE_NW_05_MIA_2021_0009/24.05.03-034 and its predecessors. The protein was dissolved in PBS for use.

Trabectedin was taken from clinical batches (Yondelis®, Pharmamar, Madrid, Spain) and applied in-label according to standard of care.

Cells

We have used primary human umbilical vein endothelial cells (HUVEC; HUVEC-c single donor, product number C-12200, Lot 433Z035.1) from PromoCell (Heidelberg, Germany) in passage 1 and at low passage numbers. HUVEC were cultured in MCDB 131 medium supplemented with 20% fetal calf serum (FCS), 2 mM glutamine (Gibco, Eggenstein, Germany), 50 µg/ml endothelial cell growth supplement (ECGS; Sigma, Taufkirchen, Germany), 5 U/ml heparin (Sigma, Taufkirchen, Germany), and maintained at 37°C in 5% CO 2 and high humidity. Cell culture dishes were coated with 0.2% gelatine. The human HT1080 fibrosarcoma cell line (RRID:CVCL_0317) was from ATCC (Manassas, VA, USA), cultured in Dulbecco´s medium (Gibco) supplemented with 10% FCS. Cell line identity was authenticated and confirmed by short tandem repeat (STR) profiling.

Animals

Procedures on animals were performed in agreement with German regulations (Tierversuchsgesetz §8, Abs. 2) and specifically approved in form of a project license.

CD-1® nude mice (RRID:IMSR_CRL:088) were from Charles River Laboratories (Sulzfeld, Germany) and acclimated to our animal-experiment facility for at least 1 week before any experimentation. Mice were maintained in individually-ventilated cages (IVC) on a 12:12 h light:dark cycle in a low-stress environment (22°C, 50% humidity, low noise) and given food and water ad libitum. Mouse experiments were done under strict 3R (Replace, Reduce, Refine) principles. Thus, in view of the therapeutic data with combinations of doxorubicin and tTF-NGR, the experiment with the sequence of trabectedin and tTF-NGR was performed once.

Preclinical and translational methodology

Phosphatidylserine (PS) staining of endothelial and tumor cells by flow cytometry, Factor X activation by tTF-NGR in the presence of cells with different amounts of phosphatidylserine (PS) on the surface and tumor xenograft experiments were essentially performed as previously described [1-6]. Factor X activation by tTF-NGR without HUVEC was assayed as validated with regard to accuracy, precision (repeatability and intermediate precision) in our laboratory (details in IMPD on file).

Corticosteroid influence on sensitizing HUVEC for tTF-NGR pro-coagulatory activity

Cells were seeded at a density of $2.4x10^4$ cells/cm^2 and grown for 24 h before starting of treatments. Trabectedin (Yondelis®, Pharmamar, Madrid, Spain) was purchased in residual amounts from the local dispensary and upon treatment diluted with complete cell culture medium to the appropriate concentration. Dexamethasone (Fortecortin®, Merck, Darmstadt, Germany) was purchased in residual amounts from the local dispensary and upon treatment diluted with complete cell culture medium to the appropriate concentration. Combination treatment of trabectedin and dexamethasone was carried out either simultaneously or in sequence, according to different time schedules.

Annexin V- and propidium iodide staining and flow cytometry analysis

Annexin V- and propidium iodide staining is widely used as a standard assay for cellular (early) apoptosis, as increase of phosphatidylserine (PS) in the outer leaflet of the phospholipid bilayer of a cellular membrane (detected by annexin V) is observed to be directly connected with early apoptosis of the cells. Briefly, after the defined post-incubation time of trabectedin- and/or corticosteroid-treatment, cells were harvested, washed in phosphate-buffered saline (PBS) and each $1x10^5$ cells were resuspended in 500 µl binding buffer [4]. To measure the surface concentration of PS on early apoptotic cells, samples were stained with annexin V fluorescein isothiocyanate (FITC) at a final concentration of 2.5 µl / $1x10^5$ cells in 100 µl (FITC annexin V Apoptosis Detection Kit I, RRID:AB_2869082, 556547, Becton Dickinson, Franklin Lakes, NJ, USA) according to the manufacturer's instructions. To distinguish between early apoptotic cells with intact cellular membranes and necrotic or late-apoptotic cells with cellular membranes destroyed and thus permeable for intracellular material such as nucleic acids, 2.5 µl of the nucleic acid-binding propidium iodide (PI, RRID:AB_2869082) were added to each sample. The cells were subsequently incubated for 15 minutes at room temperature in the dark. For cytometry analysis, we used a FACS Celesta flow cytometer (RRID:SCR_019597, Becton Dickinson); cells were washed with ice-cold PBS and finally resuspended in 500 µl ice-cold binding

buffer before analysis. For each measurement, 1×10^4 cells were counted and results were analyzed with the FACS Diva software (Becton Dickinson).

Factor X activation by tTF-NGR in the presence of cells with different amounts of phosphatidylserine (PS) on the cell surface

The ability of tTF-NGR to enhance the specific proteolytic activation of factor (F) X by FVIIa was measured by FX activation analysis as basically described by Ruf et al. [1]. In order to assess the differences in the pro-coagulatory efficacy of tTF-NGR upon binding to HUVEC and tumor cells (HT1080) with different amounts of PS on their surfaces, we have modified the assay as follows: briefly, 20 µl of the following components were added to each well in a microtiter plate: (a) 50 nM recombinant FVIIa (Novo-Nordisc, Bagsværd, Denmark) in Tris-buffered saline (TBS) containing 0.1 % bovine serum albumin (BSA); (b) 750 pM tTF-NGR in TBS-BSA; (c) 25 mM $CaCl_2$, and in place of phospholipids (d) 10,000 trabectedin-treated or control cells. To inhibit PS on cell surfaces, cells were incubated with 10 µg/10^5 cells of recombinant human annexin V (RRID:AB_2869069, Becton Dickinson, San Jose, CA, USA) for 15 min before any reagents were added. After 10 min at room temperature, the substrate FX (Enzyme Research Laboratories, Swansea, UK) was added (final concentration 1 µM). After further 10 min, the reaction was stopped in 100 mM EDTA and Spectrozyme FXa (American Diagnostica, Greenwich, USA; final concentration 0.7 mM) was added immediately prior to analysis on a microplate reader (Victor X3, PerkinElmer, Shelton, CT, USA). The rates of FXa generation were monitored by the development of color at 405 nm, the FX activation by tTF-NGR on cell surfaces was calculated by increase in extinction. Pro-coagulatory activity within the assay without addition of trabectedin and/or annexin V was set as 100 %.

Later, as for the experiments with dexamethasone, tTF-NGR pro-coagulation activity tests have been performed with a modified assay procedere with the commercially available ACTICHROME® TF-activity assay kit (Ref. 846, Bio-Medica Diagnostics, distribution via Loxo, Dossenheim, Germany) which was modified as follows: briefly, the following components were added to each well in a microtiter plate: (a) in place of phospholipids: 10 µl suspension of 10,000-30,000 trabectedin-(and dexamethasone)-treated or control cells; (b) 40 µl assay buffer; (c) 25 µl of 0.6 to 6.0 pM tTF-NGR in assay buffer; (d) 25 µl human factor VIIa; (e) 25 µl human factor X. As a control, a prefabricated phospholipid-emulsion (Rossix AB, Mölndal, Schweden) was applied. After 15 min of incubation at 37°C and slightly agitation (200 rpm), 25 µl of the substrate Spectrozyme FXa were added and incubated for further 30 min at 37°C and 200 rpm. The enzyme/substrate reaction starts upon addition of the Spectrozyme FXa, turning the solution yellow over time. Finally, the reaction was stopped by adding 50 µl of glacial acetic acid and the absorbance of the solutions was read in a microplate reader (Victor X3, PerkinElmer, Shelton, CT, USA) at a wavelength of 405 nm. The rates of FXa generation were monitored by the development of color at 405 nm, the FX activation by tTF-NGR on cell surfaces was calculated by increase in extinction. Pro-coagulatory activity within the assay without addition of trabectedin and/or annexin V was set as 100 %.

Internalization of tTF-NGR upon binding to CD13

tTF-NGR

Cloning, expression and purification of His_{tag}-tTF-NGR_{1-218}-GNGRAHA has been described in detail before [2, 3, 6]. Experiments were carried out with clinical grade tTF-NGR material from our GMP facility [5].

Flow cytometry analysis of internalization of the complex tTF-NGR:CD13 into HUVEC cells

HUVEC were harvested, washed with PBS/10 % FCS and adjusted to a final concentration of $1x10^5$ cells in 100 µl PBS. After re-centrifugation, cells were directly resuspended in a solution of tTF-NGR (15 µM) and anti-His-Alexa488-antibody (1.25 µg/ml; RRID:AB_877447, Bio-Rad), which was mixed and incubated for 10 min at room temperature in the dark before allowing to generate the [tTF-NGR-anti-His-Alexa488]-complex. This mixture of HUVEC and [tTF-NGR-anti-His-Alexa488]-complex incubated for 3 h at 37°C and 5 % CO_2. For isotype control, solely the anti-His-Alexa488-antibody (RRID:AB_877447) was applied. Subsequently, cells were washed twice with PBS/10 % FCS and resuspended in either 500 µl ice-cold PBS/10 % FCS ("w/o trypan blue") or 500 µl ice-old PBS/10 % FCS with 0.02 % trypan blue ("w/ trypan blue"). By adding trypan blue, all external fluorescence is quenched, revealing only internally bound/internalized fluorescence. For cytometry analysis, we used a FACS Celesta flow cytometer (Becton Dickinson); for each measurement, $1x10^4$ cells were counted and results were analyzed with the FACS Diva software (Becton Dickinson) and with the Flowjo Software (Becton Dickinson).

To evaluate the effect of trabectedin on CD13-expression by HUVEC, the tTF-NGR binding to CD13, and tTF-NGR:CD13 internalization into HUVEC, HUVEC were seeded at a density of $2.4x10^4$ cells/cm^2 and grown for 24-48 h before starting the treatment with trabectedin. The preincubation of the cells was performed with 10 nM trabectedin for 24 h at 37°C. The flow cytometric analysis of trabectedin-treated cells and untreated control cells was carried out as described in the section above; more precisely by incubation with the [tTF-NGR-anti-His-Alexa488]-complex to assay tTF-NGR binding and by addition of trypan blue dye to identify the ratio of internalized tTF-NGR. To determine the effect of trabectedin on CD13 expression by HUVEC, 2 µl of the Abcam anti-CD13-PE antibody (RRID:AB_1209272) was used per $1x10^5$ preincubated cells for 30 min at 4° C. The ab91357 (RRID:AB_2888649) was used as isotype control. Cytometry analysis was performed using a FACS Celesta flow cytometer (Becton Dickinson). For each measurement, $1x10^4$ cells were counted and results were analyzed with both the FACS Diva software (Becton Dickinson) and with the Flowjo Software (Becton Dickinson).

Microscopic analysis of tTF-NGR:CD13 internalization into HUVEC on coverslips

HUVEC were seeded in a 24-well plate at $2.4x10^4$ cells/cm² and cultivated on gelatine-coated coverslips overnight. Immediately before internalization experiments, tTF-NGR was directly fluorescence-labeled with Alexa-488 dye according to the protocol of the Abcam Alexa Fluor 488 conjugation kit for 100 µg protein (Abcam, product number: ab236553). Briefly, 100 µg of tTF-NGR was mixed carefully with 10 µl of Modifier Reagent and directly added to one vial of the lyophilized Alexa Fluor 488 material (100 µg-Conjugation-Mix) and resuspended by pipetting up and down for several times. After incubating for 15 min at room temperature in the dark, 10 µl Quenching Reagent

was added, mixed and the solution was ready for use after further 5 minutes. Directly Alexa488-labeled tTF-NGR was then diluted to a concentration of 15 µM with serum-free medium and incubated on HUVEC for 3 h at 37°C, 5 % CO_2. Subsequently, cells were washed with phosphate-buffered saline (PBS), fixed with ice-cold 4 % paraformaldehyde (PFA), stained with Hoechst33342, mounted with Dako fluorescence mounting medium and photographed both on a *Zeiss Axioskop* (processing software: Zeiss Axiovision) and on a *Leica SP8* confocal microscope with *HC PL APO CS2 363/1.40 oil* objective lens (processing software: Leica LAS X Lightning).

Pharmacokinetics (PK) and human anti-fusion protein antibodies (HAFA)

An enzyme-linked immunosorbent assay (ELISA) was used for quantitative determination of tTF-NGR levels in human plasma in a modification of a method described [5]. The analytical method using a commercially available IMUBIND® Tissue Factor ELISA Kit (BioMedica Diagnostics, Windsor, Canada; product number 845) was validated with regard to accuracy, precision (repeatability and intermediate precision), as well as long-term and freeze-thaw stability (full validation report in the IMPD on file).

Comparison of the tTF-NGR plasma levels with the pro-coagulatory efficacy of tTF-NGR studying serum samples from patients of the phase I study and from the safety cohort of TRABTRAP

The validated IMUBIND® Tissue Factor ELISA Kit (BioMedica Diagnostics) has been used to determine the tTF-NGR levels in the study-patients´ plasma at various time points. Subsequently, identical amounts of tTF-NGR in sera from phase I and TRABTRAP patients were applied in a TF-activation assay to determine the residual TF activity 24 h after the first tTF-NGR application of patients which received between 0.5 and 3 mg/m² tTF-NGR within the phase I study (tTF-NGR monotherapy) and the safety cohort of TRABTRAP, respectively. The TF-activation assay is a validated in-house assay on the basis of the commercially available Human Tissue Factor Activity Kit (ACTICHROME® TF; Biomedica Diagnostics), and phospholipids (Rossix AB). It is used within our IMP release procedure to assess the identity and potency of the drug product tTF-NGR. Evaluation is done via the linear regression between absorption [OD/sec] (endpoint determination after incubation for 30 min) and the concentration (pM; TF concentration range between 0 and 6 pM); the slope of the regression line m is the absolute dimensionless numeric value for the TF activity. The residual TF activity 24 h after the first tTF-NGR application was determined in % where the 0-hour value was set to 100 % (n=7, tTF-NGR monotherapy; n=9, TRABTRAP study).

HAFA testing

Human-Anti-Fusionprotein Antibody (HAFA) testing was performed exactly according to the ELISA method described for the phase I study [5]. HAFA positive sera from phase I patients were always tested as positive assay controls in parallel to the sera from TRABTRAP patients.

Statistics

For statistical significances of differences between the different groups we used the t-test or Mann-Whitney rank sum test for independent groups. Two-tailed P values lower than 0.05 were considered as indicating significant differences. Data are presented in means with SEM, or standard deviation (SD), respectively as indicated.

Suppl. References

1. Ruf W, Rehemtulla A, Morrissey JH, Edgington TS. Phospholipid-independent and -dependent interactions required for tissue factor receptor and cofactor function. J Biol Chem. 1991; 266:2158–2166, (erratum J Biol Chem 1991; 266:16256).
2. Bieker R, Kessler T, Schwöppe C, Padró T, Persigehl T, Bremer C, Dreischalück J, Kolkmeyer A, Heindel W, Mesters RM, Berdel WE. Infarction of tumor vessels by NGR-peptide directed targeting of tissue factor. Experimental results and first-in-man experience. Blood. 2009; 113:5019–5027.
3. Schwöppe C, Kessler T, Persigehl T, Liersch R, Hintelmann H, Dreischalück J, Ring J, Bremer C, Heindel W, Mesters RM, Berdel WE. Tissue-factor fusion proteins induce occlusion of tumor vessels. Thrombosis Res. 2010; 125: S143–S150.
4. Schellenberger EA, Bogdanov A, Petrovsky A, Ntziachristos V, Weissleder R, Josephson L. Optical Imaging of Apoptosis as a Biomarker of Tumor Response to Chemotherapy. Neoplasia. 2003; 5:187–192.
5. Schliemann C, Gerwing M, Heinzow H.S, Harrach S, Schwöppe C, Wildgruber M, Hansmeier AA, Angenendt L, Berdel AF, Stalmann U, Berning B, Kratz-Albers K, Middelberg-Bisping K, Wiebe S, Albring J, Wilms C, Hartmann W, Wardelmann E, Krähling T, Heindel W, Gerss J, Bormann E, Schmidt H, Lenz G, Kessler T, Mesters RM, Berdel WE. First-in-class CD13-targeted tissue factor tTF-NGR in patients with recurrent or refractory malignant tumors: results of a phase I dose-escalation study. Cancers 2020; 12, 1488. https://doi.org/10.3390/cancers12061488.
6. Stucke-Ring J, Ronnacker J, Brand C, Höltke C, Schliemann C, Kessler T, Schmidt LH, Harrach S, Mantke V, Hintelmann H, Hartmann W, Wardelmann E, Lenz G, Wünsch B, Müller-Tidow C, Mesters RM, Schwöppe C, Berdel WE. Combinatorial effects of doxorubicin and retargeted tissue factor by intratumoral entrapment of doxorubicin and proapoptotic increase of tumor vascular infarction. Oncotarget 2016; 7, 82458–82472. DOI: 10.18632/oncotarget.12559.

Study design and flow chart of patients - TRABTRAP Safety Cohort

A

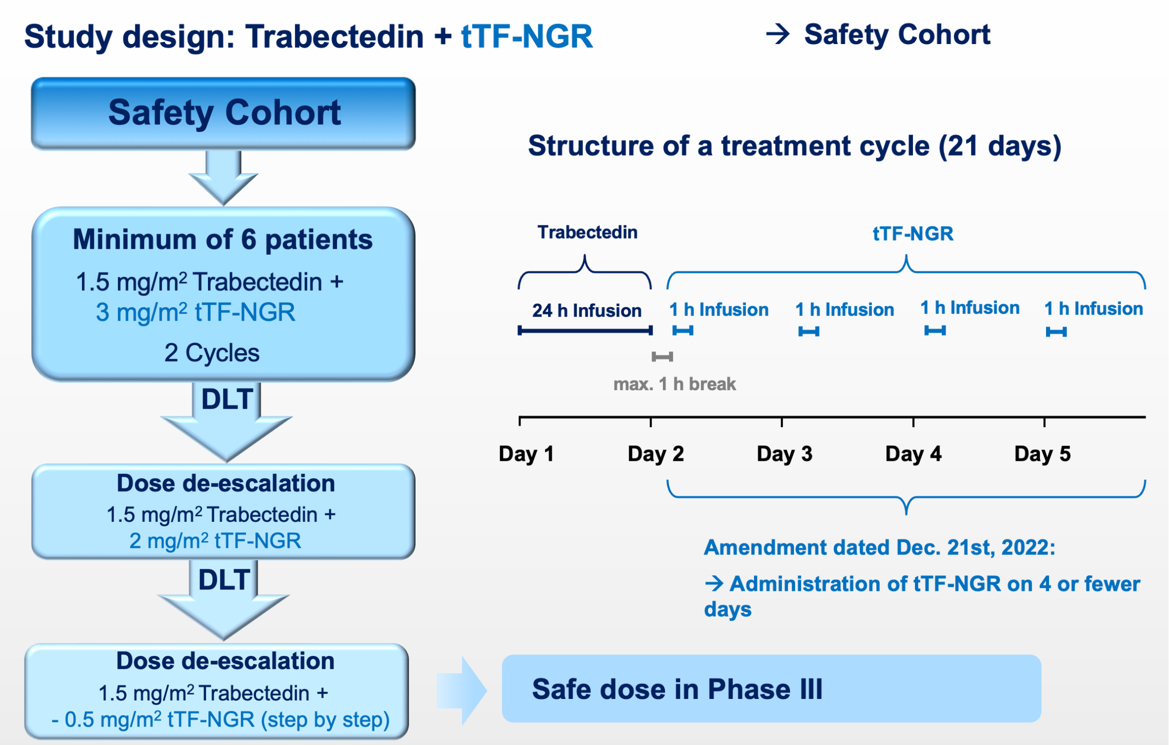

B

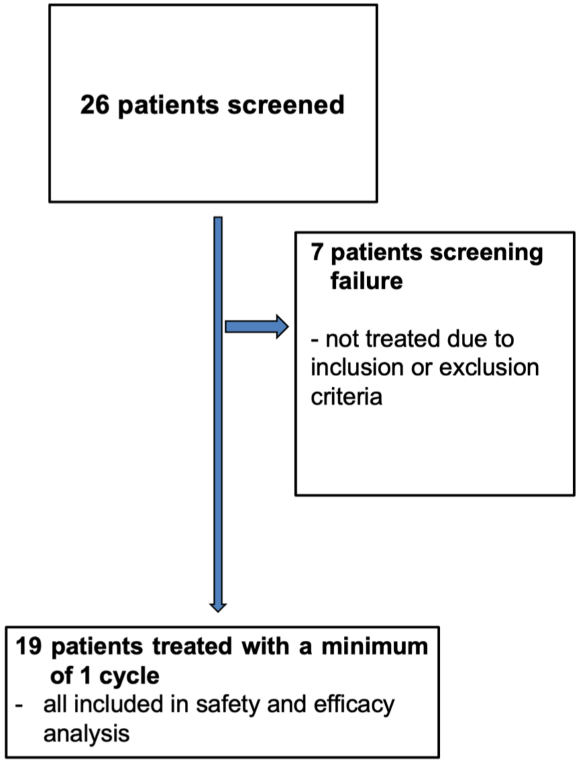

A, Study design of the TRABTRAP safety cohort; **B**, Flow chart diagram of patients in the safety cohort

Supplementary Clinical and pharmacokinetic results

Case Reports

In the following we summarize *brief case reports* (date 2025 09 27) focusing on safety aspects for the patients going by tTF-NGR dose level. The patient numbers represent those given in Table 1.

Patient number **1)** After cycle 2 with trabectedin and tTF-NGR at a dose level of **3.0 mg/m²**, a male patient's (table 1: **synovial sarcoma, m/21**) CT-scan showed evidence of a pulmonary artery embolism at the segmental level in the left upper and lower lobes. The patient did not have concomitant symptomatology. The IMP was withdrawn and anticoagulation with enoxaparin was started. On a CT scan 3 months later, embolism was no longer detectable. The investigator suspected a reasonable causal relationship with tTF-NGR, not with trabectedin. Pulmonary embolism according to clinically observed Reference Safety Information (RSI) was unexpected for tTF-NGR, the case was reported as SUSAR, grade 3. As per protocol and upon DSMB consultation, the dose level of tTF-NGR was reduced for further patients to 2.0 mg/m^2. In retrospect, inclusion criteria were violated since the patient had experienced a subclavian vein thrombosis a year before entry on trial. The patient later died due to progressive disease.

2) After cycle 2 with trabectedin and tTF-NGR at a dose level of **2.0 mg/m²**, a male patient (**leiomyosarcoma, m/59**) presented to the out-patient day clinic with no complaints, but elevated troponin T hs in the lab-report and ECG showing T-wave inversion in V3-V4. Subsequent cardiological consultation and admission via the emergency department for overnight monitoring followed. Echocardiography and a cardiac MRT did not show specific pathology. The patient received acetylsalicylic acid and remained free of any symptoms. This was formally considered as non-ST-elevation myocardial infarction (N-STEMI), possibly related to tTF-NGR. 'Acute myocardial infarction' and 'Electrocardigram T wave inversion' are unexpected for tTF-NGR, while 'troponin T increased' is already listed in the RSI as expected. The IMP was withdrawn and under anticoagulation with acetylsalicylic acid the findings resolved within a few days. In the next cycle of therapy trabectedin monotherapy was given. With terminal negative T-waves in ECG and one pathological biomarker (troponin T hs) occurring also after this trabectedin monotherapy, formal criteria for a transient N-STEMI were stated also upon trabectedin monotherapy, which allows the hypothesis, that trabectedin had a role in the development of the transient N-STEMI event observed after the TRABTRAP combination. This event again after a few days was resolved completely, but did not change the decision of the investigators upon DSMB consultation to further reduce the tTF-NGR dose in the following patients of the study to 1.5 mg/m². The patient later died due to progressive disease.

3) At a tTF-NGR dose level of **1.5 mg/m²**, a female patient (**leiomyosarcoma, f/61**) developed an isolated troponin T hs value increase to a maximum of 92.9 ng/L (lower pathological limit 50 ng/L) after two applications of tTF-NGR in cycle 1. The substance according to protocol (version 16) was not given on days 3 and 4. The troponin T hs increase was not followed by any clinical symptoms or other parameters of clinical significance. The value decreased to below the pathological limit on day 9 of the cycle (resolved/recovered). Two short term applications of dexamethasone (4

mg orally) showed an immediate decrease of the troponin T hs values. According to protocol version 16 this was reported as SAR. The second cycle was given with trabectedin without tTF-NGR. No relevant increase of troponin T hs values was observed. As per protocol, the dose level of tTF-NGR for further patients was reduced to 1.0 mg/m^2.

4) A male patient (**liposarcoma, m/65**) treated at the tTF-NGR dose level of **1.0 mg/m^2** tolerated 2 cycles well. On day 18 of the 2nd cycle troponin T hs increased to 283 ng/L (pathological limit >50 ng/L) with no further sequelae and subsequent decrease. There was no reasonable relation to tTF-NGR stated as the event evolved more than 7 days after the last tTF-NGR application in cycle 2 (detected on day 13 after last tTF-NGR application) and the terminal half-life of tTF-NGR is approx. 9 hours, which means that the troponin T hs increase occurred after more than 18 terminal half-lifes, which together with the internalization kinetics of the tTF-NGR:CD13 complex into endothelial cells observed excluded any presence of the IMP in the organism. Accordingly, in all previous patients with troponin T hs increase within the phase I study and this safety cohort the time of increase was during the application or considerably sooner after the last application. Possible relation to trabectedin was discussed as the terminal half-life of trabectedin is approx. 7.5 days and similar cardiac troponin elevations have been observed before. This constellation did not define a DLT according to protocol. The patient later died due to progressive disease.

5) A 2nd patient (**synovial sarcoma, m/51**) treated at the dose level of **1.0 mg/m^2** tTF-NGR tolerated 3 cycles of the combination without DLT, SUSAR or SAE, but was then taken off study due to iPD. The patient later died due to progressive disease.

6) A 3rd patient (**liposarcoma, m/52**) tolerated 3 cycles (tTF-NGR **1.0 mg/m^2**) without DLT, but showed in the CT after 3 cycles a small PORT-associated thrombus, which did not obstruct the superior vena cava and was asymptomatic. This was reported as SAR grade 2, reasonably related to the catheter and also the IMP. The patient received low molecular heparin followed by apixaban. The 4th cycle was given as trabectedin monotherapy since the patient had iSD with minor tumor lesion shrinkage. Follow-up CT showed size decrease of the asymptomatic thrombus. In addition, there were small peripheral pulmonary lesions with undulating size, which could not be clearly categorized by the roentgenologist during the observation. During the period of observation values for troponin T hs correlated well with those of troponin I hs (details not shown). The patient died 6 months after EoT due to tumor progression.

7) A 4th patient (**leiomyosarcoma, f/40**) tolerated 5 cycles (tTF-NGR **1.0 mg/m^2**) of the combination without side effects. A selective inhibition of tumor perfusion was measured in the phase I study at dose levels higher than, but also at a dose of 1.0 mg/m^2 of tTF-NGR. To test this again for the dose level of 1.0 mg/m^2 tTF-NGR we have repeated our dynamic multiparameter magnetic resonance imaging (MRI) protocol as published in the phase I report. Imaging confirmed selective inhibition of tumor blood perfusion as observed before (Fig. 1 A in the main publication). Figure 1 further shows the size reduction of target liver and kidney lesions after 3 cycles in this patient (Fig. 1 B), counted as a RECIST stable disease since a 3rd lesion showed less shrinkage. Two further cycles of the combination were applied without problems, but with larger intervals according to the personal plans of the patient. The 6th cycle had to be postponed due to elevated transaminases according to standard of care using trabectedin. Subsequently, restaging of the tumor lesions after 2 weeks of postponement of cycle 6 showed SD in RECIST staging but clinically symptomatic tumor progression (abdominal metastasis) which led to hospitalization of the patient and was reported as SAE, non-related to the IMP but to tumor progression.

8) A 5th patient (**leiomyosarcoma, m/53**) with a long list of preceding therapies (Table 1), treated with the combination (tTF-NGR **1.0 mg/m²**), showed an increase of troponin T hs from 12.6 ng/L (cycle 1, day 1) to 16.6 ng/L (cycle 1, day 3) and 33.4 ng/L (cycle 1, day 4). Clinical symptoms (nausea, fatigue, headache, chest heaviness) were non-specific and ECG was normal. Although troponin T hs value did not reach pathological range (>50 ng/L) this increase into the grey zone of the marker (14 - 50 ng/L), according to amended protocol version 17 (chapter 6.1.4), was interpreted by the investigator as a safety biomarker and it was decided to pause tTF-NGR on days 4 and 5 of the first cycle. Acetylsalicylic acid (500 mg) was given on day 4 and Ibuprofen 2x400 mg/day for the following days. The maximum troponin T hs value was 35 ng/L and decreased before cycle 2. This was reported as AE. A DSMB meeting was held and among other topics this case was discussed and it was decided to re-expose the patient at the dose level of 0.5 mg/m² in the 2nd cycle provided the troponin T hs levels further decreased. With a troponin T hs level of 21.6 ng/L on day 1 of cycle 2, tTF-NGR was restarted at a dose of 0.5 mg/m². IMP exposure for 3 days were without major events, but daily clinical symptoms of fatigue and "heavy chest" as described above for the first cycle. On day 5 of cycle 2 the last dose of tTF-NGR was withheld since the troponin T hs values steeply increased to 122 ng/L. Clinically, the patient reported symptoms of fatigue, a "heavy chest" and slight pain in the thorax when inhaling. Clinically auscultation showed signs of bronchial obstruction with other clinical findings and an ECG being normal and unchanged. For safety reasons a "triple exclusion" CT-scan was performed. This showed iSD according to iRECIST with slight size increase of pulmonary metastasis, the known intrapulmonary scars after 2 episodes of pulmonary resection of multiple lung metastases (total > 70 lesions surgically removed, the majority from the right lower lobe), and also showed a segmental pulmonary arterial embolism in the right lower lobe. Coronaries and other visible blood vessels including the aorta were normal. The patient was anticoagulated with one dose of acetylsalicylic acid (500 mg) and daily therapeutic doses of enoxaparin. He remained cardiopulmonary stable, the peripheral capillary SaO_2 always being >90%. The event, despite extensive previous surgery in this area, was reported as SUSAR, since pulmonary embolism according to Reference Safety Information (RSI) was formally still unexpected for tTF-NGR. The first report stated grade 2, since medical intervention was necessary but not urgent. Due to the symptoms and the prolonged time of hospitalization this was later upgraded to grade 3, which defined a DLT. Highest troponin T hs level was 224 ng/L upon which the values decreased. ECG remained normal, symptoms improved and a sonography revealed an asymptomatic DVT in the right leg without complete occlusion of vessels as possible source of the embolism. During observation however, the patient suffered from progressive disease manifested as occlusion of the left main bronchus by hilar metastasis, which had to be re-opened bronchoscopically. A follow-up CT showed no remaining pulmonary embolism (resolved/recovered). A DSMB convened on which the study results on the dose level of 1 mg/m² tTF-NGR were discussed. The patient later died due to progressive disease.

9) Upon decision of the investigators and the DSMB the next patient (**malignant peripheral nerve sheath tumor, m/44**) in this safety cohort was treated as the 1st with trabectedin plus tTF-NGR at **1.0 mg/m² for 2 days** (on days 2 and 3 of the regimen). The patient tolerated this protocol without DLT. Restaging after 3 cycles of therapy showed a slight increase of the target lesion. Thus, the patient was taken off study. In retrospect, the patient developed a slight increase of troponin T hs values into the "grey zone" (maximum value 23 ng/L) without further tendency to increase and also a minimal decrease of cardiac ejection fraction (LVEF = 47%) without loss of normal physical and working activity. This was reported as AE grade 1. As troponin T hs elevations in all 3 cycles occurred between day 1 and day 2 of the cycle under trabectedin monotherapy with some

recovery under tTF-NGR, and as troponin T hs increases and even the development of symptomatic cardiac dysfunction is known for trabectedin monotherapy as reported in the SmPC, any relation to tTF-NGR remains questionable.

10) A 2nd patient (**synovial sarcoma, f/38**) was treated with trabectedin plus tTF-NGR at **1.0 mg/m^2 for 2 days** (on days 2 and 3 of the regimen). The patient tolerated this protocol without DLT. However, in cycle 2 the patient had to be briefly admitted to day hospital with a clinical and roentgenological tumor and metastasis progress. This was reported as SAE. A minor troponin T hs increase to 78 ng/L under clinical deterioration, which was normalized at the next control and had no clinical sequelae was interpreted by the investigator as due to the tumor progress with deterioration of patient´s performance and not as DLT. The patient died after 2 cycles due to fast tumor progression.

11) A 3rd patient (**myxoid liposarcoma, m/34**) was treated with trabectedin plus tTF-NGR at **1.0 mg/m^2 for 2 days** (on days 2 and 3 of the regimen). The patient so far tolerated 12 cycles of this protocol without DLT. However, the following cycles had to be postponed due to trabectedin-induced thrombocytopenia and was given with reduced trabectedin dose (1.2 mg/m^2 and 1.0 mg/m^2). All restagings including after 45 weeks revealed SD with some tumor shrinkage.

12) A 4th patient (**synovial sarcoma, f/30**) was treated with trabectedin plus tTF-NGR at **1.0 mg/m^2 for 2 days** (on days 2 and 3 of the regimen) and tolerated >2 cycles without DLT. Restaging after 9 and 18 weeks showed a good partial remission. The last day of tTF-NGR in cycle 4 was omitted, as the patient developed a febrile episode, which was treated as out-patient with antibiotics. Further, since the PORT aspiration was blocked after cycle 4, the patient was anticoagulated for an assumed PORT outlet thrombus and cycles 5 and 6 were administered without tTF-NGR. However, in follow-up CTs no PORT thrombus could be detected. This was anyway reported as SAE grade 2 and reasonably related to the IMP. After 6 cycles the patient remained in PR but left the study for personal reasons.

13) A 5th patient (**leiomyosarcoma, m/60**) was treated with trabectedin plus tTF-NGR at **1.0 mg/m^2 for 2 days** (on days 2 and 3 of the regimen). Clinical tolerability was without problems. On day 5 the troponin T hs increased to 38 ng/L. The patient was given acetylsalicylic acid for few days and reported back on day 10 without symptoms. Laboratory values on this day were unreliable because of delayed testing, in other examinations and clinically there were no major pathologic findings. On day 16, ECG showed atrial fibrillation with a central heart frequency of 155/min, a peripheral pulse of 90/min and a decreased RR of 90/60. Although the patient reported only slight shortness of breath at higher levels of physical activity, he was admitted and a CT scan was performed. CT showed a massive tumor progression including the lung metastasis, with subtotal occlusion of the mediastinal superior vena cava and tumor infiltration of the right upper pulmonary artery. CT also revealed a subsegmental arterial pulmonary embolism and moderate signs of right heart strain. The patient was hemodynamically stable and always had SaO_2 levels above 92% at room air. Shortly after, the ECG showed sinus rhythm and the patient decided to leave the hospital under apixaban treatment. The DSMB was convened and DSMB members as well as investigators reported this event as SUSAR grade 3 and DLT. A further dose reduction to 0.5 mg/m^2 tTF-NGR given on days 2 and 3 was decided. In follow up CT examinations the pulmonary embolism was completely resolved/recovered. The patient later died due to progressive disease.

14) A 1st patient (**synovial sarcoma, f/34**) was treated with trabectedin plus tTF-NGR at **0.5 mg/m^2 for 2 days** (on days 2 and 3 of the regimen) without problems for 3 cycles. Restaging after 9 weeks showed an iUPD in imaging. Although Hounsfield units indicated liquid material in some of the progressive metastatic lesions, clinical progression was obvious. The patient was further treated with best supportive care. The patient died after 2 cycles due to fast tumor progression.

15) A 2nd patient (**leiomyosarcoma, f/53**) was treated with trabectedin plus tTF-NGR **at 0.5 mg/m^2 for 2 days** (on days 2 and 3 of the regimen) without problems for 2 cycles. Restaging after 9 weeks showed iUPD. According to protocol therapy was continued for a total of 6 cycles with SAEs (grade 2 anemia) reported in cycles 3, 4 and 6. The investigator reported the anemia as being related to both tTF-NGR and trabectedin. As the patient was transfused in-house, it was reported as SUSAR grade 2. Restaging after 18 weeks and 6 cycles showed iPD and the patient was taken off study.

16) A 3rd patient (**uterine leiomyosarcoma, f/53**) was treated with trabectedin plus tTF-NGR at **0.5 mg/m^2 for 2 days** (on days 2 and 3 of the regimen) without problems for 2 cycles. Restaging after 9 and 18 weeks showed SD with some tumor shrinkage. The patient has obtained 14 cycles and was taken off study with iPD from nadir of the RECIST measurements after 45 weeks of treatment.

17) A 4th patient (**leiomyosarcoma, f/64**) was included into the trial although extensive previous lines of therapy (cumulative doxorubicin dose 450 mg/m^2 bsa) led to cardiomyopathy with reduced left ventricular ejection fraction, but recovering during observation. The first cycle (**tTF-NGR at 0.5 mg/m^2 for 2 days**) was tolerated without clinical problems, however the troponin I hs values upon falling tendency before trabectedin (to 2.1 pg/mL) started slowly increasing after trabectedin and before tTF-NGR (3.2 pg/mL) with slow further increase and crossing the ULN (24 pg/mL) with 29.7 pg/mL 12 days after end of tTF-NGR application. With further slow increase during the rest period (highest value 45.8 pg/mL) without any clinical symptoms and other signs of a coronary event and with the half-life times for tTF-NGR and trabectedin as described above, this was interpreted as non-coronary myocardial impairment related to trabectedin and together with the elevation of transaminases led to a prolongation of observation between cycles 1 and 2 of 1 week by the investigators decision according to protocol. The second cycle was then given with reduced dose of 1.2 mg/m^2 trabectedin and 0.5 mg/m^2 of tTF-NGR on days 2 and 3. The normalized troponin I hs at the start of the cycle showed a slow increase during the next days with 39.4 pg/mL on day 8 and normal CK. On day 15 the patient presented with pretibial edemas (grade 1) as only clinical symptom and with an elevated troponin T hs of 480 pg/mL. In addition, CK was increased from normal values to 837 U/L (ULN 170 U/L), however with CK MB of 35 U/L (CK/CK MB percentage of 4.5% (< 6%). The patient obtained acetylsalicylic acid and was admitted for further observation. A cardiology consult revealed an ECG with right bundle branch block (preexisting) without significant changes to the screening ECG and a normal cardiac ultrasound examination. AngioCT excluded pulmonary embolism. On the next morning troponin I was slowly further increasing to 563 pg/mL. CK with peak of 1137 U/L (CK MB 59 U/L and CK/CK MB percentage of 5.1%) decreased to 527 U/L with CK MB to 28 U/L (5.3%). Although the troponin I increase was more pronounced than in cycle 1, but slow and several half-lifes after end of tTF-NGR application and because of the peripheral edemas and a NT-proBNP on day 16 with 4004 pg/mL (ULN <125 pg/mL), the events were interpreted by the center cardiologists and the investigators as non-specific myocardial impairment (cardiomyopathy) reasonably related to and described for trabectedin. Accordingly,

cardiac MRT showed discretely elevated relaxation times. Due to overnight admission, the troponin I hs increase was reported as SAE grade 3 related to trabectedin, not related to tTF-NGR and not as DLT. TP1 tumor control after 9 weeks showed a partial remission (PR) in all lesions. Thus, further cycles with a trabectedin dose further reduced to 1.0 mg/m^2 were given. These cycles have also been tolerated without DLT and with considerably less deviations of the laboratory values (3rd cycle peak values: CK 26 U/L normal, NT-pro-BNP 3399 pg/mL then decreasing, troponin I hs 8.0 pg/mL normal). This underlines that the observed laboratory deviations in cycle 2 were related to trabectedin and not to any vascular events caused by tTF-NGR and that interpretation of troponin value increase must include previous case history, steepness and time course of the increase. Finally, it is interesting that the D-dimer values increased in each cycle with a peak in cycle 2 of 13 mg/L FEU (ULN 0.5 mg/L FEU) with decreasing peaks in cycle 3 (peak 4.92 mg/L FEU). D-dimer is not offered as value in CTCAE 5 and could be interpreted as fibrinolysis marker of intratumoral coagulation and not simply as danger sign for pulmonary embolism (excluded by CT). Observation of further patients will show, whether D-dimer could be used as a predictive marker for response to this combination. At week 27 after 8 cycles the CT showed further decreasing tumor lesions, at week 45 after 14 cycles the patient remained in an ongoing PR. The patient at that time had to be taken off study, since she had to be anticoagulated due to a grade 2 deep vein thrombosis, but remained under trabectedin monoptherapy.

18) A 5th patient (**myxoid sarcoma, f/62**) was treated with trabectedin plus tTF-NGR at **0.5 mg/m^2 for 2 days** (on days 2 and 3 of the regimen) without problems for >2 cycles. Restaging after 9 and 18 weeks showed a stable disease with tumor shrinkage. The patient remained under therapy without disease progression until week 36. The patient then had to be taken off study, since she had to be anticoagulated due to a grade 2 vein thrombosis on the site of the vascular access detected by CT before start of cycle 13.

19) A 6th patient (**fibrosarcoma, m/58**) was accepted into the trial although the last doxorubicin had caused troponin T hs levels of > 25 ng/L with decreasing tendency. The patient tolerated the first 2 cycles with trabectedin plus tTF-NGR at **0.5 mg/m^2 for 2 days** (on days 2 and 3 of the regimen) without DLT. Two SAEs were reported, which were not reasonably related to tTF-NGR but related to trabectedin (Table 1). After cycle 2 the patient showed progressive disease and was taken off study. The patient later died due to progressive disease.

UPN	MedDRA PT	MedDRA SOC	Start Date of AE	End Date of AE	Ongoing?	Serious (Yes/ No)	CTCAE Grading (v5.0)	AE considered as DLT	Relationship to tTF-NGR	Relationship to Trabectedin	Action taken with tTF-NGR	Action taken with Trabectedin	Outcome
1	Transaminases increased	Investigations	2021-11-24	2022-01-14	.	No	Grade 3 (Severe)	No	No reasonable possibility/ Not applicable	Reasonable possibility	Dose not Changed	Unknown	Recovered/resolved
1	Headache	Nervous system disorders	2021-11-23	2021-11-26	.	No	Grade 1 (Mild)	No	No reasonable possibility/ Not applicable	No reasonable possibility/ Not applicable	Dose not Changed	Drug Interrupted	Recovered/resolved
1	Visual impairment	Eye disorders	2021-11-24	2021-11-26	.	No	Grade 1 (Mild)	No	No reasonable possibility/ Not applicable	No reasonable possibility/ Not applicable	Dose not Changed	Not Applicable	Recovered/resolved
1	Constipation	Gastrointestinal disorders	2021-11-28	2021-11-29	.	No	Grade 2 (Moderate)	No	No reasonable possibility/ Not applicable	No reasonable possibility/ Not applicable	Not Applicable	Not Applicable	Recovered/resolved
1	Pulmonary embolism	Respiratory, thoracic and mediastinal disorders	2021-12-28	2022-03-23	.	Yes	Grade 3 (Severe)	Yes	Reasonable possibility	No reasonable possibility/ Not applicable	Drug Withdrawn	Drug Withdrawn	Recovered/resolved
2	Transaminases increased	Investigations	2022-03-23	2022-05-04	.	No	Grade 3 (Severe)	No	No reasonable possibility/ Not applicable	Reasonable possibility	Drug Interrupted	Unknown	Recovered/resolved
2	Troponin T increased	Investigations	2022-03-29	2023-10-26	.	Yes	Grade 3 (Severe)	Yes	Reasonable possibility	Reasonable possibility	Drug Withdrawn	Dose not Changed	Recovering/resolving
2	Hyperuricaemia	Metabolism and nutrition disorders	2022-04-14	2022-08-22	.	No	Grade 1 (Mild)	No	No reasonable possibility/ Not applicable	No reasonable possibility/ Not applicable	Dose not Changed	Dose not Changed	Recovered/resolved
2	Acute myocardial infarction	Cardiac disorders	2022-03-29	2022-04-11	.	Yes	Grade 3 (Severe)	Yes	Reasonable possibility	Reasonable possibility	Drug Withdrawn	Drug Withdrawn	Recovered/resolved
2	Electrocardiogram T wave inversion	Investigations	2022-03-29	2022-05-10	.	No	Grade 2 (Moderate)	No	Reasonable possibility	No reasonable possibility/ Not applicable	Dose not Changed	Dose not Changed	Recovered/resolved
3	Troponin T increased	Investigations	2022-08-18	2022-09-12	.	Yes	Grade 3 (Severe)	Yes	Reasonable possibility	Reasonable possibility	Drug Interrupted	Dose not Changed	Recovering/resolving
3	Neutrophil count decreased	Investigations	2022-09-14	2022-09-26	.	No	Grade 3 (Severe)	No	No reasonable possibility/ Not applicable	Reasonable possibility	Not Applicable	Dose not Changed	Recovered/resolved
3	Platelet count decreased	Investigations	2022-09-12	2022-09-26	.	No	Grade 2 (Moderate)	No	No reasonable possibility/ Not applicable	Reasonable possibility	Not Applicable	Dose not Changed	Recovered/resolved
3	Abdominal pain upper	Gastrointestinal disorders	2022-09-14	2022-09-23	.	No	Grade 2 (Moderate)	No	No reasonable possibility/ Not applicable	No reasonable possibility/ Not applicable	Dose not Changed	Dose not Changed	Recovered/resolved
3	Diarrhoea	Gastrointestinal disorders	2022-09-12	2022-09-14	.	No	Grade 2 (Moderate)	No	No reasonable possibility/ Not applicable	No reasonable possibility/ Not applicable	Dose not Changed	Dose not Changed	Recovered/resolved
3	Hypertension	Vascular disorders	2022-09-26	2022-10-04	.	No	Grade 3 (Severe)	No	No reasonable possibility/ Not applicable	No reasonable possibility/ Not applicable	Dose not Changed	Dose not Changed	Recovered/resolved
3	Neutrophil count decreased	Investigations	2022-10-04	2022-10-17	.	No	Grade 2 (Moderate)	No	No reasonable possibility/ Not applicable	Reasonable possibility	Dose not Changed	Dose not Changed	Recovered/resolved
3	Platelet count decreased	Investigations	2022-09-16	2022-09-26	.	No	Grade 3 (Severe)	No	No reasonable possibility/ Not applicable	Reasonable possibility	Dose not Changed	Dose not Changed	Recovered/resolved
3	Neutrophil count decreased	Investigations	2022-09-23	2022-09-26	.	No	Grade 2 (Moderate)	No	No reasonable possibility/ Not applicable	Reasonable possibility	Dose not Changed	Dose not Changed	Recovered/resolved

3	Platelet count decreased	Investigations	2022-11-03	.	Ongoing	No	Grade 1 (Mild)	No	No reasonable possibility/ Not applicable	Reasonable possibility	Dose not Changed	Dose not Changed	Not recovered/ not resolved
4	Alanine aminotransferase increased	Investigations	2022-09-28	2022-11-10	.	No	Grade 4 (Life-threatening)	No	No reasonable possibility/ Not applicable	Reasonable possibility	Drug Interrupted	Dose not Changed	Recovered/resolved
4	Aspartate aminotransferase increased	Investigations	2022-09-28	2022-11-14	.	No	Grade 4 (Life-threatening)	No	No reasonable possibility/ Not applicable	Reasonable possibility	Drug Interrupted	Dose not Changed	Recovered/resolved
4	Pyrexia	General disorders and administration site conditions	2022-09-27	2022-09-27	.	No	Grade 1 (Mild)	No	Reasonable possibility	No reasonable possibility/ Not applicable	Dose not Changed	Dose not Changed	Recovered/resolved
4	Chills	General disorders and administration site conditions	2022-09-27	2022-09-27	.	No	Grade 1 (Mild)	No	Reasonable possibility	No reasonable possibility/ Not applicable	Dose not Changed	Dose not Changed	Recovered/resolved
4	Gamma-glutamyltransferase increased	Investigations	2022-09-30	2022-10-21	.	No	Grade 1 (Mild)	No	No reasonable possibility/ Not applicable	Reasonable possibility	Dose not Changed	Dose not Changed	Not recovered/ not resolved
4	Anaemia	Blood and lymphatic system disorders	2022-11-22	.	Ongoing	No	Grade 2 (Moderate)	No	No reasonable possibility/ Not applicable	Reasonable possibility	Dose not Changed	Dose not Changed	Recovering/resolving
4	Troponin T increased	Investigations	2022-11-03	.	Ongoing	Yes	Grade 3 (Severe)	No	No reasonable possibility/ Not applicable	Reasonable possibility	Drug Interrupted	Dose Reduced	Not recovered/ not resolved
4	Nausea	Gastrointestinal disorders	2022-10-19	2022-10-27	.	No	Grade 2 (Moderate)	No	No reasonable possibility/ Not applicable	Reasonable possibility	Dose not Changed	Dose Reduced	Recovered/resolved
4	Gamma-glutamyltransferase increased	Investigations	2022-10-27	2022-11-15	.	No	Grade 2 (Moderate)	No	No reasonable possibility/ Not applicable	Reasonable possibility	Dose not Changed	Dose not Changed	Not recovered/ not resolved
4	Gamma-glutamyltransferase increased	Investigations	2022-11-22	.	Ongoing	No	Grade 3 (Severe)	No	No reasonable possibility/ Not applicable	Reasonable possibility	Dose not Changed	Dose not Changed	Not recovered/ not resolved
5	Nausea	Gastrointestinal disorders	2023-03-21	2023-03-22	.	No	Grade 1 (Mild)	No	No reasonable possibility/ Not applicable	Reasonable possibility	Dose not Changed	Dose not Changed	Recovered/resolved
5	Aspartate aminotransferase increased	Investigations	2023-03-22	2023-05-16	.	No	Grade 2 (Moderate)	No	No reasonable possibility/ Not applicable	Reasonable possibility	Dose not Changed	Dose not Changed	Recovered/resolved
5	Alanine aminotransferase increased	Investigations	2023-03-23	2023-05-22	.	No	Grade 3 (Severe)	No	No reasonable possibility/ Not applicable	Reasonable possibility	Dose not Changed	Dose not Changed	Recovered/resolved
5	White blood cell count decreased	Investigations	2023-04-14	2023-05-03	.	No	Grade 3 (Severe)	No	No reasonable possibility/ Not applicable	Reasonable possibility	Dose not Changed	Dose not Changed	Recovering/resolving
7	Nausea	Gastrointestinal disorders	2023-05-31	2023-06-03	.	No	Grade 2 (Moderate)	No	No reasonable possibility/ Not applicable	Reasonable possibility	Dose not Changed	Dose not Changed	Recovered/resolved
7	Aspartate aminotransferase increased	Investigations	2023-06-03	2023-06-22	.	No	Grade 2 (Moderate)	No	No reasonable possibility/ Not applicable	Reasonable possibility	Dose not Changed	Dose not Changed	Not recovered/ not resolved
7	Alanine aminotransferase increased	Investigations	2023-06-02	2023-06-21	.	No	Grade 2 (Moderate)	No	No reasonable possibility/ Not applicable	Reasonable possibility	Dose not Changed	Dose not Changed	Not recovered/ not resolved

7	Gamma-glutamyltransferase increased	Investigations	2023-06-09	2023-07-14	.	No	Grade 2 (Moderate)	No	No reasonable possibility/ Not applicable	Reasonable possibility	Dose not Changed	Dose not Changed	Not recovered/ not resolved
7	Neutrophil count decreased	Investigations	2023-07-25	2023-08-10	.	No	Grade 3 (Severe)	No	No reasonable possibility/ Not applicable	Reasonable possibility	Dose not Changed	Dose not Changed	Recovered/resolved
7	Blood alkaline phosphatase increased	Investigations	2023-05-30	.	Ongoing	No	Grade 2 (Moderate)	No	No reasonable possibility/ Not applicable	No reasonable possibility/ Not applicable	Dose not Changed	Dose not Changed	Not recovered/ not resolved
7	Gamma-glutamyltransferase increased	Investigations	2023-07-14	2023-12-05	.	No	Grade 3 (Severe)	No	No reasonable possibility/ Not applicable	Reasonable possibility	Dose not Changed	Dose not Changed	Recovered/resolved
7	Alanine aminotransferase increased	Investigations	2023-06-21	2023-08-16	.	No	Grade 3 (Severe)	No	No reasonable possibility/ Not applicable	Reasonable possibility	Dose not Changed	Dose not Changed	Not recovered/ not resolved
7	Alanine aminotransferase increased	Investigations	2023-08-16	2023-11-17	.	No	Grade 4 (Life-threatening)	No	No reasonable possibility/ Not applicable	Reasonable possibility	Dose not Changed	Dose Reduced	Recovered/resolved
7	Aspartate aminotransferase increased	Investigations	2023-06-22	2023-12-05	.	No	Grade 3 (Severe)	No	No reasonable possibility/ Not applicable	Reasonable possibility	Dose not Changed	Dose not Changed	Recovered/resolved
7	Abdominal pain upper	Gastrointestinal disorders	2023-09-07	2023-09-07	.	No	Grade 2 (Moderate)	No	No reasonable possibility/ Not applicable	No reasonable possibility/ Not applicable	Drug Withdrawn	Not Applicable	Recovered/resolved
7	Blood bilirubin increased	Investigations	2023-09-07	2023-09-08	.	No	Grade 2 (Moderate)	No	No reasonable possibility/ Not applicable	No reasonable possibility/ Not applicable	Drug Withdrawn	Dose not Changed	Recovered/resolved
7	Abdominal pain	Gastrointestinal disorders	2023-10-03	2023-10-06	.	Yes	Grade 3 (Severe)	No	No reasonable possibility/ Not applicable	No reasonable possibility/ Not applicable	Drug Withdrawn	Drug Withdrawn	Recovered/resolved
7	Lipase increased	Investigations	2023-10-03	2023-10-23	.	No	Grade 4 (Life-threatening)	No	No reasonable possibility/ Not applicable	No reasonable possibility/ Not applicable	Drug Withdrawn	Drug Withdrawn	Recovered/resolved
8	Neutrophil count decreased	Investigations	2023-09-13	2023-09-18	.	No	Grade 4 (Life-threatening)	No	No reasonable possibility/ Not applicable	Reasonable possibility	Dose not Changed	Dose not Changed	Recovered/resolved
8	Cough	Respiratory, thoracic and mediastinal disorders	2023-09-06	.	Ongoing	No	Grade 1 (Mild)	No	No reasonable possibility/ Not applicable	No reasonable possibility/ Not applicable	Dose not Changed	Dose not Changed	Not recovered/ not resolved
8	Aspartate aminotransferase increased	Investigations	2023-09-20	2023-10-02	.	No	Grade 2 (Moderate)	No	No reasonable possibility/ Not applicable	Reasonable possibility	Dose not Changed	Dose not Changed	Recovered/resolved
8	Embolism	Vascular disorders	2023-09-22	2024-04-08	.	Yes	Grade 3 (Severe)	No	Reasonable possibility	Reasonable possibility	Drug Withdrawn	Drug Interrupted	Recovered/resolved
8	Alanine aminotransferase increased	Investigations	2023-09-20	2023-10-06	.	No	Grade 3 (Severe)	No	No reasonable possibility/ Not applicable	Reasonable possibility	Dose not Changed	Dose not Changed	Recovered/resolved
15	Alanine aminotransferase increased	Investigations	2024-08-02	2024-12-02	.	No	Grade 1 (Mild)	No	No reasonable possibility/ Not applicable	No reasonable possibility/ Not applicable	Dose not Changed	Dose not Changed	Recovered/resolved
15	Aspartate aminotransferase increased	Investigations	2024-08-02	2024-12-03	.	No	Grade 1 (Mild)	No	No reasonable possibility/ Not applicable	No reasonable possibility/ Not applicable	Dose not Changed	Dose not Changed	Recovered/resolved

15	Anaemia	Blood and lymphatic system disorders	2024-08-02	2024-08-22	.	No	Grade 1 (Mild)	No	Reasonable possibility	Reasonable possibility	Dose not Changed	Dose not Changed	Not recovered/ not resolved
15	Anaemia	Blood and lymphatic system disorders	2024-08-23	2024-09-30	.	Yes	Grade 2 (Moderate)	No	Reasonable possibility	Reasonable possibility	Dose not Changed	Dose not Changed	Not recovered/ not resolved
15	Anaemia	Blood and lymphatic system disorders	2024-09-30	2024-10-02	.	Yes	Grade 1 (Mild)	No	Reasonable possibility	Reasonable possibility	Dose not Changed	Dose not Changed	Not recovered/ not resolved
15	Anaemia	Blood and lymphatic system disorders	2024-10-02	2024-10-21	.	Yes	Grade 2 (Moderate)	No	Reasonable possibility	Reasonable possibility	Dose not Changed	Dose not Changed	Not recovered/ not resolved
15	Anaemia	Blood and lymphatic system disorders	2024-10-21	2024-11-11	.	No	Grade 1 (Mild)	No	Reasonable possibility	Reasonable possibility	Dose not Changed	Dose not Changed	Not recovered/ not resolved
15	Anaemia	Blood and lymphatic system disorders	2024-11-11	2024-12-03	.	Yes	Grade 2 (Moderate)	No	Reasonable possibility	Reasonable possibility	Dose not Changed	Dose not Changed	Not recovered/ not resolved
15	Anaemia	Blood and lymphatic system disorders	2024-12-03	.	Ongoing	No	Grade 1 (Mild)	No	No reasonable possibility/ Not applicable	No reasonable possibility/ Not applicable	Dose not Changed	Dose not Changed	Not recovered/ not resolved
15	White blood cell count decreased	Investigations	2024-08-21	.	Ongoing	No	Grade 2 (Moderate)	No	No reasonable possibility/ Not applicable	No reasonable possibility/ Not applicable	Dose not Changed	Dose not Changed	Not recovered/ not resolved
15	Lethargy	Nervous system disorders	2024-09-10	.	Ongoing	No	Grade 1 (Mild)	No	No reasonable possibility/ Not applicable	No reasonable possibility/ Not applicable	Dose not Changed	Dose not Changed	Not recovered/ not resolved
15	Dysaesthesia	Nervous system disorders	.	.	Ongoing	No	Grade 1 (Mild)	No	Reasonable possibility	Reasonable possibility	Dose not Changed	Dose not Changed	Not recovered/ not resolved
15	Myalgia	Musculoskeletal and connective tissue disorders	2024-09-13	2024-09-16	.	No	Grade 1 (Mild)	No	Reasonable possibility	Reasonable possibility	Dose not Changed	Dose not Changed	Recovered/resolved
15	Gamma-glutamyltransferase increased	Investigations	2024-09-12	2024-10-21	.	No	Grade 1 (Mild)	No	Reasonable possibility	Reasonable possibility	Dose not Changed	Dose not Changed	Recovered/resolved
15	Gamma-glutamyltransferase increased	Investigations	2024-12-03	.	Ongoing	No	Grade 1 (Mild)	No	No reasonable possibility/ Not applicable	No reasonable possibility/ Not applicable	Dose not Changed	Dose not Changed	Not recovered/ not resolved
15	Myalgia	Musculoskeletal and connective tissue disorders	2024-10-03	2024-10-06	.	No	Grade 1 (Mild)	No	Reasonable possibility	Reasonable possibility	Dose not Changed	Dose not Changed	Recovered/resolved
14	Arthralgia	Musculoskeletal and connective tissue disorders	2024-07-08	2024-07-21	.	No	Grade 1 (Mild)	No	No reasonable possibility/ Not applicable	No reasonable possibility/ Not applicable	Dose not Changed	Dose not Changed	Recovered/resolved
14	Nausea	Gastrointestinal disorders	2024-07-10	2024-07-21	.	No	Grade 2 (Moderate)	No	No reasonable possibility/ Not applicable	Reasonable possibility	Dose not Changed	Dose not Changed	Recovered/resolved
14	Vomiting	Gastrointestinal disorders	2024-07-13	2024-07-16	.	No	Grade 1 (Mild)	No	No reasonable possibility/ Not applicable	Reasonable possibility	Dose not Changed	Dose not Changed	Recovered/resolved
14	Fatigue	General disorders and administration site conditions	2024-07-10	2024-07-25	.	No	Grade 1 (Mild)	No	No reasonable possibility/ Not applicable	Reasonable possibility	Dose not Changed	Dose not Changed	Recovered/resolved
14	Leukopenia	Blood and lymphatic system disorders	2024-07-22	2024-07-25	.	No	Grade 2 (Moderate)	No	No reasonable possibility/ Not applicable	Reasonable possibility	Dose not Changed	Dose not Changed	Recovered/resolved
14	Dizziness	Nervous system disorders	2024-07-22	2024-07-25	.	No	Grade 1 (Mild)	No	No reasonable possibility/ Not applicable	Reasonable possibility	Dose not Changed	Dose not Changed	Recovered/resolved

14	Abdominal pain upper	Gastrointestinal disorders	2024-07-22	2024-07-25	.	No	Grade 1 (Mild)	No	No reasonable possibility/ Not applicable	Reasonable possibility	Dose not Changed	Dose not Changed	Recovered/resolved
14	Abdominal pain upper	Gastrointestinal disorders	2024-07-26	2024-08-01	.	No	Grade 2 (Moderate)	No	No reasonable possibility/ Not applicable	No reasonable possibility/ Not applicable	Dose not Changed	Dose not Changed	Recovering/resolving
14	Abdominal pain upper	Gastrointestinal disorders	2024-08-01	2024-09-22	.	No	Grade 1 (Mild)	No	No reasonable possibility/ Not applicable	No reasonable possibility/ Not applicable	Dose not Changed	Dose not Changed	Recovered/resolved
14	Vomiting	Gastrointestinal disorders	2024-07-31	2024-07-31	.	No	Grade 1 (Mild)	No	Reasonable possibility	No reasonable possibility/ Not applicable	Dose not Changed	Dose not Changed	Recovered/resolved
14	Aspartate aminotransferase increased	Investigations	2024-08-02	2024-09-05	.	No	Grade 3 (Severe)	No	No reasonable possibility/ Not applicable	Reasonable possibility	Dose not Changed	Dose not Changed	Not recovered/ not resolved
14	Alanine aminotransferase increased	Investigations	2024-08-02	2024-09-05	.	No	Grade 3 (Severe)	No	No reasonable possibility/ Not applicable	Reasonable possibility	Dose not Changed	Dose not Changed	Not recovered/ not resolved
14	Decreased appetite	Metabolism and nutrition disorders	2024-08-05	2024-09-22	.	No	Grade 2 (Moderate)	No	No reasonable possibility/ Not applicable	No reasonable possibility/ Not applicable	Dose not Changed	Dose not Changed	Not recovered/ not resolved
14	Fatigue	General disorders and administration site conditions	2024-08-16	2024-09-22	.	No	Grade 1 (Mild)	No	No reasonable possibility/ Not applicable	Reasonable possibility	Dose not Changed	Dose not Changed	Not recovered/ not resolved
14	Vomiting	Gastrointestinal disorders	2024-08-16	2024-08-21	.	No	Grade 1 (Mild)	No	No reasonable possibility/ Not applicable	Reasonable possibility	Dose not Changed	Dose not Changed	Recovered/resolved
14	Vomiting	Gastrointestinal disorders	2024-08-22	2024-08-23	.	No	Grade 2 (Moderate)	No	No reasonable possibility/ Not applicable	Reasonable possibility	Dose not Changed	Dose not Changed	Recovered/resolved
14	C-reactive protein increased	Investigations	2024-08-30	2024-09-03	.	No	Grade 2 (Moderate)	No	No reasonable possibility/ Not applicable	No reasonable possibility/ Not applicable	Dose not Changed	Dose not Changed	Recovered/resolved
14	Pain	General disorders and administration site conditions	2024-08-27	2024-09-22	.	No	Grade 2 (Moderate)	No	No reasonable possibility/ Not applicable	No reasonable possibility/ Not applicable	Dose not Changed	Dose not Changed	Not recovered/ not resolved
14	Anaemia	Blood and lymphatic system disorders	2024-09-02	2024-09-05	.	No	Grade 3 (Severe)	No	No reasonable possibility/ Not applicable	Reasonable possibility	Dose not Changed	Dose not Changed	Recovered/resolved
14	Leukopenia	Blood and lymphatic system disorders	2024-09-02	2024-09-10	.	No	Grade 3 (Severe)	No	No reasonable possibility/ Not applicable	Reasonable possibility	Dose not Changed	Dose not Changed	Recovered/resolved
14	Vomiting	Gastrointestinal disorders	2024-08-30	2024-09-22	.	No	Grade 2 (Moderate)	No	No reasonable possibility/ Not applicable	Reasonable possibility	Dose not Changed	Dose not Changed	Not recovered/ not resolved
14	Gamma-glutamyltransferase increased	Investigations	2024-08-16	2024-08-30	.	No	Grade 1 (Mild)	No	No reasonable possibility/ Not applicable	Reasonable possibility	Dose not Changed	Dose Reduced	Recovered/resolved
14	Gamma-glutamyltransferase increased	Investigations	2024-08-30	2024-09-02	.	No	Grade 2 (Moderate)	No	No reasonable possibility/ Not applicable	Reasonable possibility	Dose Reduced	Dose not Changed	Recovered/resolved
14	Gamma-glutamyltransferase increased	Investigations	2024-09-02	2024-09-02	.	No	Grade 3 (Severe)	No	No reasonable possibility/ Not applicable	Reasonable possibility	Dose not Changed	Dose not Changed	Recovered/resolved
14	Leukopenia	Blood and lymphatic system disorders	2024-09-02	2024-09-10	.	No	Grade 3 (Severe)	No	No reasonable possibility/ Not applicable	Reasonable possibility	Dose not Changed	Dose not Changed	Recovered/resolved

17	Nausea	Gastrointestinal disorders	2024-10-22	2024-10-23	.	No	Grade 2 (Moderate)	No	No reasonable possibility/ Not applicable	Reasonable possibility	Dose not Changed	Dose not Changed	Recovered/resolved
17	Alanine aminotransferase increased	Investigations	2024-10-23	2024-11-14	.	No	Grade 3 (Severe)	No	No reasonable possibility/ Not applicable	Reasonable possibility	Dose not Changed	Dose not Changed	Recovered/resolved
17	Aspartate aminotransferase increased	Investigations	2024-10-23	2024-10-28	.	No	Grade 3 (Severe)	No	No reasonable possibility/ Not applicable	Reasonable possibility	Dose not Changed	Dose not Changed	Recovered/resolved
17	Gamma-glutamyltransferase increased	Investigations	2024-10-23	2024-10-28	.	No	Grade 2 (Moderate)	No	No reasonable possibility/ Not applicable	Reasonable possibility	Dose not Changed	Dose not Changed	Recovered/resolved
17	Fibrin D dimer increased	Investigations	2024-10-23	2024-10-28	.	No	Grade 3 (Severe)	No	No reasonable possibility/ Not applicable	Reasonable possibility	Dose not Changed	Dose not Changed	Recovered/resolved
17	Aspartate aminotransferase increased	Investigations	2024-10-28	2024-11-18	.	No	Grade 2 (Moderate)	No	No reasonable possibility/ Not applicable	Reasonable possibility	Dose not Changed	Dose not Changed	Recovered/resolved
17	Fibrin D dimer increased	Investigations	2024-10-28	2024-11-19	.	No	Grade 2 (Moderate)	No	No reasonable possibility/ Not applicable	Reasonable possibility	Dose not Changed	Dose not Changed	Recovered/resolved
17	Gamma-glutamyltransferase increased	Investigations	2024-10-28	2024-11-19	.	No	Grade 3 (Severe)	No	No reasonable possibility/ Not applicable	Reasonable possibility	Dose not Changed	Dose not Changed	Recovered/resolved
17	Activated partial thromboplastin time prolonged	Investigations	2024-10-28	2024-11-04	.	No	Grade 3 (Severe)	No	No reasonable possibility/ Not applicable	Reasonable possibility	Dose not Changed	Dose not Changed	Recovered/resolved
17	Diarrhoea	Gastrointestinal disorders	2024-10-30	2024-11-02	.	No	Grade 1 (Mild)	No	Reasonable possibility	Reasonable possibility	Dose not Changed	Dose not Changed	Recovered/resolved
17	Nausea	Gastrointestinal disorders	2024-11-19	2024-11-22	.	No	Grade 2 (Moderate)	No	No reasonable possibility/ Not applicable	Reasonable possibility	Dose not Changed	Dose not Changed	Recovered/resolved
17	Fibrin D dimer increased	Investigations	2024-11-20	2024-11-25	.	No	Grade 4 (Life-threatening)	No	Reasonable possibility	No reasonable possibility/ Not applicable	Dose not Changed	Dose Reduced	Recovered/resolved
17	Fibrin D dimer increased	Investigations	2024-11-25	2024-12-02	.	No	Grade 1 (Mild)	No	Reasonable possibility	No reasonable possibility/ Not applicable	Dose not Changed	Dose not Changed	Recovered/resolved
17	Blood alkaline phosphatase increased	Investigations	2024-11-04	2024-11-25	.	No	Grade 1 (Mild)	No	Reasonable possibility	No reasonable possibility/ Not applicable	Dose not Changed	Dose not Changed	Recovered/resolved
17	Blood alkaline phosphatase increased	Investigations	2024-11-25	2024-12-18	.	No	Grade 2 (Moderate)	No	Reasonable possibility	No reasonable possibility/ Not applicable	Dose not Changed	Dose not Changed	Recovered/resolved
17	Alanine aminotransferase increased	Investigations	2024-11-20	2024-12-02	.	No	Grade 3 (Severe)	No	No reasonable possibility/ Not applicable	Reasonable possibility	Dose not Changed	Dose not Changed	Recovered/resolved
17	Alanine aminotransferase increased	Investigations	2024-12-02	2024-12-18	.	No	Grade 1 (Mild)	No	No reasonable possibility/ Not applicable	Reasonable possibility	Dose not Changed	Dose not Changed	Recovered/resolved
17	Aspartate aminotransferase increased	Investigations	2024-11-20	2024-11-25	.	No	Grade 4 (Life-threatening)	No	No reasonable possibility/ Not applicable	Reasonable possibility	Dose not Changed	Dose not Changed	Recovered/resolved

17	Aspartate aminotransferase increased	Investigations	2024-11-25	2024-12-02	.	No	Grade 3 (Severe)	No	No reasonable possibility/ Not applicable	Reasonable possibility	Dose not Changed	Dose not Changed	Recovered/resolved
17	Aspartate aminotransferase increased	Investigations	2024-12-02	2024-12-18	.	No	Grade 2 (Moderate)	No	No reasonable possibility/ Not applicable	Reasonable possibility	Dose not Changed	Dose not Changed	Recovered/resolved
17	Gamma-glutamyltransferase increased	Investigations	2024-11-20	2024-11-25	.	No	Grade 2 (Moderate)	No	No reasonable possibility/ Not applicable	Reasonable possibility	Dose not Changed	Dose not Changed	Recovered/resolved
17	Gamma-glutamyltransferase increased	Investigations	2024-11-25	.	Ongoing	No	Grade 3 (Severe)	No	No reasonable possibility/ Not applicable	Reasonable possibility	Dose not Changed	Dose not Changed	Unknown
17	Troponin I increased	Investigations	2024-11-07	2024-11-18	.	No	Grade 1 (Mild)	No	No reasonable possibility/ Not applicable	Reasonable possibility	Dose not Changed	Dose not Changed	Recovered/resolved
17	Troponin I increased	Investigations	2024-11-20	2024-12-02	.	No	Grade 1 (Mild)	No	Reasonable possibility	Reasonable possibility	Dose not Changed	Dose not Changed	Recovered/resolved
17	Fibrin D dimer increased	Investigations	2024-11-20	2024-11-25	.	No	Grade 4 (Life-threatening)	No	No reasonable possibility/ Not applicable	Reasonable possibility	Dose not Changed	Dose not Changed	Recovered/resolved
17	Fibrin D dimer increased	Investigations	2024-11-25	2024-12-02	.	No	Grade 1 (Mild)	No	No reasonable possibility/ Not applicable	Reasonable possibility	Dose not Changed	Dose not Changed	Recovered/resolved
17	Fibrin D dimer increased	Investigations	2024-12-02	2025-01-06	.	No	Grade 2 (Moderate)	No	No reasonable possibility/ Not applicable	Reasonable possibility	Dose not Changed	Dose not Changed	Recovered/resolved
17	Fibrin D dimer increased	Investigations	2025-01-08	2025-01-13	.	No	Grade 3 (Severe)	No	No reasonable possibility/ Not applicable	Reasonable possibility	Dose not Changed	Dose not Changed	Recovered/resolved
17	Leukopenia	Blood and lymphatic system disorders	2024-11-04	2024-11-07	.	No	Grade 3 (Severe)	No	No reasonable possibility/ Not applicable	Reasonable possibility	Dose not Changed	Dose Reduced	Recovered/resolved
17	Troponin I increased	Investigations	2024-12-02	2024-12-09	.	Yes	Grade 3 (Severe)	No	No reasonable possibility/ Not applicable	Reasonable possibility	Drug Interrupted	Drug Interrupted	Recovered/resolved
17	Troponin I increased	Investigations	2024-12-09	2025-01-06	.	No	Grade 2 (Moderate)	No	No reasonable possibility/ Not applicable	Reasonable possibility	Dose not Changed	Dose not Changed	Recovered/resolved
17	Blood creatine phosphokinase increased	Investigations	2024-12-02	2024-12-03	.	No	Grade 3 (Severe)	No	No reasonable possibility/ Not applicable	Reasonable possibility	Drug Interrupted	Drug Interrupted	Recovered/resolved
17	Blood creatine phosphokinase increased	Investigations	2024-12-03	2024-12-18	.	No	Grade 2 (Moderate)	No	No reasonable possibility/ Not applicable	Reasonable possibility	Dose not Changed	Dose not Changed	Recovered/resolved
17	N-terminal prohormone brain natriuretic peptide increased	Investigations	2024-12-02	2024-12-09	.	No	Grade 3 (Severe)	No	No reasonable possibility/ Not applicable	Reasonable possibility	Dose not Changed	Dose Reduced	Recovered/resolved
17	N-terminal prohormone brain natriuretic peptide increased	Investigations	2024-12-09	2025-01-13	.	No	Grade 2 (Moderate)	No	No reasonable possibility/ Not applicable	Reasonable possibility	Dose not Changed	Dose not Changed	Recovered/resolved
17	N-terminal prohormone brain	Investigations	2025-01-13	.	Ongoing	No	Grade 3 (Severe)	No	No reasonable possibility/ Not applicable	Reasonable possibility	Dose not Changed	Dose not Changed	Recovered/resolved

	natriuretic peptide increased												
17	Oedema peripheral	General disorders and administration site conditions	2024-11-25	2024-12-17	.	No	Grade 2 (Moderate)	No	No reasonable possibility/ Not applicable	Reasonable possibility	Dose not Changed	Dose not Changed	Recovered/resolved
17	Oedema peripheral	General disorders and administration site conditions	2024-12-18	.	Ongoing	No	Grade 1 (Mild)	No	No reasonable possibility/ Not applicable	Reasonable possibility	Dose not Changed	Dose not Changed	Recovered/resolved
17	Blood alkaline phosphatase increased	Investigations	2025-01-06	.	Ongoing	No	Grade 2 (Moderate)	No	Reasonable possibility	No reasonable possibility/ Not applicable	Dose not Changed	Dose not Changed	Unknown
17	Aspartate aminotransferase increased	Investigations	2025-01-08	2025-01-27	.	No	Grade 1 (Mild)	No	No reasonable possibility/ Not applicable	Reasonable possibility	Dose not Changed	Dose not Changed	Unknown
17	Leukopenia	Blood and lymphatic system disorders	2025-01-27	2025-02-03	.	No	Grade 2 (Moderate)	No	No reasonable possibility/ Not applicable	Reasonable possibility	Drug Interrupted	Drug Interrupted	Recovered/resolved
6	Device related thrombosis	General disorders and administration site conditions	2023-06-15	.	Ongoing	Yes	Grade 2 (Moderate)	No	Reasonable possibility	Reasonable possibility	Not Applicable	Dose not Changed	Recovering/resolving
6	Arrhythmia	Cardiac disorders	2023-05-12	2023-05-12	.	No	Grade 1 (Mild)	No	Reasonable possibility	Reasonable possibility	Dose not Changed	Dose not Changed	Recovered/resolved
6	Gamma-glutamyltransferase increased	Investigations	2023-04-21	.	Ongoing	No	Grade 1 (Mild)	No	Reasonable possibility	Reasonable possibility	Dose not Changed	Dose not Changed	Recovering/resolving
6	Gamma-glutamyltransferase increased	Investigations	2023-04-22	.	Ongoing	No	Grade 2 (Moderate)	No	Reasonable possibility	Reasonable possibility	Dose not Changed	Dose not Changed	Recovering/resolving
6	Aspartate aminotransferase increased	Investigations	2023-04-19	2023-05-08	.	No	Grade 4 (Life-threatening)	No	No reasonable possibility/ Not applicable	Reasonable possibility	Drug Interrupted	Dose Reduced	Recovered/resolved
6	Alanine aminotransferase increased	Investigations	2023-04-19	2023-05-08	.	No	Grade 4 (Life-threatening)	No	No reasonable possibility/ Not applicable	Reasonable possibility	Drug Interrupted	Dose Reduced	Recovered/resolved
6	Blood lactate dehydrogenase increased	Investigations	2023-04-19	2023-05-08	.	No	Grade 1 (Mild)	No	Reasonable possibility	Reasonable possibility	Drug Interrupted	Dose Reduced	Recovered/resolved
9	Neutrophil count decreased	Investigations	2023-10-30	2023-11-06	.	No	Grade 3 (Severe)	No	No reasonable possibility/ Not applicable	No reasonable possibility/ Not applicable	Dose not Changed	Dose not Changed	Recovered/resolved
9	Platelet count decreased	Investigations	2023-11-20	2023-11-28	.	No	Grade 3 (Severe)	No	No reasonable possibility/ Not applicable	Reasonable possibility	Dose not Changed	Dose not Changed	Recovered/resolved
9	Anaemia	Blood and lymphatic system disorders	2023-11-27	.	Ongoing	No	Grade 3 (Severe)	No	No reasonable possibility/ Not applicable	Reasonable possibility	Dose not Changed	Dose not Changed	Recovering/resolving
9	Ejection fraction decreased	Investigations	2024-01-09	.	Ongoing	No	Grade 2 (Moderate)	No	No reasonable possibility/ Not applicable	No reasonable possibility/ Not applicable	Not Applicable	Not Applicable	Unknown
11	Platelet count decreased	Investigations	2024-01-30	2024-02-20	.	No	Grade 3 (Severe)	No	No reasonable possibility/ Not applicable	Reasonable possibility	Dose not Changed	Dose Reduced	Recovering/resolving

11	Platelet count decreased	Investigations	2024-03-05	2024-03-26	.	No	Grade 4 (Life-threatening)	No	No reasonable possibility/ Not applicable	Reasonable possibility	Dose not Changed	Dose Reduced	Recovering/resolving
11	Gastrooesophageal reflux disease	Gastrointestinal disorders	2024-02-25	2024-02-25	.	No	Grade 1 (Mild)	No	No reasonable possibility/ Not applicable	No reasonable possibility/ Not applicable	Not Applicable	Not Applicable	Recovered/resolved
11	Gamma-glutamyltransferase increased	Investigations	2024-02-02	2024-02-20	.	No	Grade 2 (Moderate)	No	No reasonable possibility/ Not applicable	Reasonable possibility	Dose not Changed	Dose Reduced	Recovered/resolved
11	Gamma-glutamyltransferase increased	Investigations	2024-01-23	2024-01-30	.	No	Grade 3 (Severe)	No	No reasonable possibility/ Not applicable	Reasonable possibility	Dose not Changed	Dose Reduced	Recovered/resolved
11	Platelet count decreased	Investigations	2024-04-09	2024-05-06	.	No	Grade 3 (Severe)	No	No reasonable possibility/ Not applicable	Reasonable possibility	Dose not Changed	Dose not Changed	Recovering/resolving
11	Platelet count decreased	Investigations	2024-05-21	2024-06-18	.	No	Grade 2 (Moderate)	No	No reasonable possibility/ Not applicable	Reasonable possibility	Dose not Changed	Dose not Changed	Recovering/resolving
11	Gamma-glutamyltransferase increased	Investigations	2024-02-27	2024-03-26	.	No	Grade 3 (Severe)	No	No reasonable possibility/ Not applicable	Reasonable possibility	Dose not Changed	Dose Reduced	Recovered/resolved
11	Gamma-glutamyltransferase increased	Investigations	2024-04-02	2024-05-06	.	No	Grade 1 (Mild)	No	No reasonable possibility/ Not applicable	Reasonable possibility	Dose not Changed	Dose not Changed	Recovered/resolved
11	Neutrophil count decreased	Investigations	2024-01-30	2024-02-07	.	No	Grade 4 (Life-threatening)	No	No reasonable possibility/ Not applicable	Reasonable possibility	Dose not Changed	Dose not Changed	Recovered/resolved
11	Neutrophil count decreased	Investigations	2024-03-05	2024-03-19	.	No	Grade 3 (Severe)	No	No reasonable possibility/ Not applicable	Reasonable possibility	Dose not Changed	Dose not Changed	Recovered/resolved
11	Neutrophil count decreased	Investigations	2024-04-09	2024-05-06	.	No	Grade 3 (Severe)	No	Reasonable possibility	Reasonable possibility	Dose not Changed	Dose not Changed	Recovered/resolved
11	Neutrophil count decreased	Investigations	2024-05-21	2024-06-18	.	No	Grade 2 (Moderate)	No	No reasonable possibility/ Not applicable	Reasonable possibility	Dose not Changed	Dose not Changed	Recovered/resolved
11	Alanine aminotransferase increased	Investigations	2024-02-27	2024-03-05	.	No	Grade 2 (Moderate)	No	No reasonable possibility/ Not applicable	Reasonable possibility	Dose not Changed	Dose not Changed	Recovering/resolving
11	Back pain	Musculoskeletal and connective tissue disorders	2024-05-25	2024-05-25	.	No	Grade 2 (Moderate)	No	No reasonable possibility/ Not applicable	No reasonable possibility/ Not applicable	Dose not Changed	Dose not Changed	Recovered/resolved
11	Platelet count decreased	Investigations	2024-07-09	2024-07-15	.	No	Grade 1 (Mild)	No	No reasonable possibility/ Not applicable	Reasonable possibility	Not Applicable	Not Applicable	Recovering/resolving
11	Lyme disease	Infections and infestations	2024-07-20	.	Ongoing	No	Grade 2 (Moderate)	No	No reasonable possibility/ Not applicable	No reasonable possibility/ Not applicable	Not Applicable	Not Applicable	Recovering/resolving
11	Platelet count decreased	Investigations	2024-07-30	2024-08-20	.	No	Grade 2 (Moderate)	No	No reasonable possibility/ Not applicable	Reasonable possibility	Not Applicable	Not Applicable	Recovering/resolving
11	Platelet count decreased	Investigations	2024-10-01	2024-10-14	.	No	Grade 2 (Moderate)	No	No reasonable possibility/ Not applicable	Reasonable possibility	Not Applicable	Not Applicable	Recovering/resolving
11	Platelet count decreased	Investigations	2024-10-29	2024-11-11	.	No	Grade 2 (Moderate)	No	No reasonable possibility/ Not applicable	Reasonable possibility	Not Applicable	Not Applicable	Recovering/resolving

11	Platelet count decreased	Investigations	2024-11-11	2024-11-26	.	No	Grade 1 (Mild)	No	No reasonable possibility/ Not applicable	Reasonable possibility	Not Applicable	Not Applicable	Recovering/resolving
11	Gastrooesophageal reflux disease	Gastrointestinal disorders	2024-11-15	2024-11-15	.	No	Grade 1 (Mild)	No	No reasonable possibility/ Not applicable	No reasonable possibility/ Not applicable	Dose not Changed	Dose not Changed	Recovered/resolved
11	Platelet count decreased	Investigations	2024-11-26	2024-12-09	.	No	Grade 2 (Moderate)	No	No reasonable possibility/ Not applicable	Reasonable possibility	Not Applicable	Not Applicable	Recovering/resolving
11	Platelet count decreased	Investigations	2024-12-09	.	Ongoing	No	Grade 1 (Mild)	No	No reasonable possibility/ Not applicable	Reasonable possibility	Not Applicable	Not Applicable	Recovering/resolving
11	Rhinorrhoea	Respiratory, thoracic and mediastinal disorders	2024-11-23	2024-11-26	.	No	Grade 1 (Mild)	No	No reasonable possibility/ Not applicable	No reasonable possibility/ Not applicable	Not Applicable	Not Applicable	Recovered/resolved
16	Nausea	Gastrointestinal disorders	2024-08-23	2024-08-28	.	No	Grade 2 (Moderate)	No	No reasonable possibility/ Not applicable	Reasonable possibility	Dose not Changed	Dose not Changed	Recovered/resolved
16	Fatigue	General disorders and administration site conditions	2024-08-21	.	Ongoing	No	Grade 1 (Mild)	No	No reasonable possibility/ Not applicable	Reasonable possibility	Dose not Changed	Dose not Changed	Not recovered/ not resolved
16	Muscle tightness	Musculoskeletal and connective tissue disorders	2024-09-02	.	Ongoing	No	Grade 1 (Mild)	No	No reasonable possibility/ Not applicable	No reasonable possibility/ Not applicable	Dose not Changed	Dose not Changed	Not recovered/ not resolved
16	Nausea	Gastrointestinal disorders	2024-09-12	2024-10-13	.	No	Grade 1 (Mild)	No	No reasonable possibility/ Not applicable	Reasonable possibility	Dose not Changed	Dose not Changed	Recovered/resolved
16	Chest discomfort	General disorders and administration site conditions	2024-10-04	2024-10-05	.	No	Grade 1 (Mild)	No	No reasonable possibility/ Not applicable	No reasonable possibility/ Not applicable	Dose not Changed	Dose not Changed	Recovered/resolved
16	White blood cell count decreased	Investigations	2024-09-26	2024-10-03	.	No	Grade 3 (Severe)	No	No reasonable possibility/ Not applicable	Reasonable possibility	Dose not Changed	Dose not Changed	Recovering/resolving
16	White blood cell count decreased	Investigations	2024-10-04	2024-10-07	.	No	Grade 2 (Moderate)	No	No reasonable possibility/ Not applicable	Reasonable possibility	Dose not Changed	Dose not Changed	Recovered/resolved
16	Neutrophil count decreased	Investigations	2024-09-26	2024-10-03	.	No	Grade 3 (Severe)	No	No reasonable possibility/ Not applicable	Reasonable possibility	Dose not Changed	Dose not Changed	Recovering/resolving
16	Neutrophil count decreased	Investigations	2024-10-04	2024-10-07	.	No	Grade 2 (Moderate)	No	No reasonable possibility/ Not applicable	Reasonable possibility	Dose not Changed	Dose not Changed	Recovered/resolved
16	Nausea	Gastrointestinal disorders	2024-10-08	2024-10-08	.	No	Grade 1 (Mild)	No	No reasonable possibility/ Not applicable	Reasonable possibility	Dose not Changed	Dose not Changed	Recovered/resolved
16	Nausea	Gastrointestinal disorders	2024-10-30	2024-11-13	.	No	Grade 1 (Mild)	No	No reasonable possibility/ Not applicable	Reasonable possibility	Dose not Changed	Dose not Changed	Recovered/resolved
16	Oedema peripheral	General disorders and administration site conditions	2024-11-25	2025-01-06	.	No	Grade 1 (Mild)	No	No reasonable possibility/ Not applicable	No reasonable possibility/ Not applicable	Dose not Changed	Dose not Changed	Recovered/resolved
16	Nausea	Gastrointestinal disorders	2024-11-26	2024-12-03	.	No	Grade 1 (Mild)	No	No reasonable possibility/ Not applicable	Reasonable possibility	Dose not Changed	Dose not Changed	Recovered/resolved
16	Nausea	Gastrointestinal disorders	2024-12-16	2024-12-23	.	No	Grade 1 (Mild)	No	No reasonable possibility/ Not applicable	Reasonable possibility	Dose not Changed	Dose not Changed	Recovered/resolved
16	Catheter site infection	Infections and infestations	2024-12-27	2025-01-03	.	No	Grade 2 (Moderate)	No	No reasonable possibility/ Not applicable	No reasonable possibility/ Not applicable	Dose not Changed	Dose not Changed	Recovered/resolved

16	Influenza	Infections and infestations	2024-12-29	2025-01-13	.		No	Grade 1 (Mild)	No	No reasonable possibility/ Not applicable	No reasonable possibility/ Not applicable	Dose not Changed	Dose not Changed	Recovered/resolved
16	Bladder irritation	Renal and urinary disorders	2025-01-27	2025-01-31	.		No	Grade 1 (Mild)	No	No reasonable possibility/ Not applicable	No reasonable possibility/ Not applicable	Dose not Changed	Dose not Changed	Recovered/resolved
18	Alanine aminotransferase increased	Investigations	2024-12-11	2024-12-17	.		No	Grade 2 (Moderate)	No	No reasonable possibility/ Not applicable	Reasonable possibility	Dose not Changed	Dose not Changed	Not recovered/ not resolved
18	Aspartate aminotransferase increased	Investigations	2024-12-11	2024-12-27	.		No	Grade 2 (Moderate)	No	No reasonable possibility/ Not applicable	Reasonable possibility	Dose not Changed	Dose not Changed	Recovered/resolved
18	Alanine aminotransferase increased	Investigations	2024-12-18	2024-12-27	.		No	Grade 3 (Severe)	No	No reasonable possibility/ Not applicable	Reasonable possibility	Dose not Changed	Dose not Changed	Recovered/resolved
18	Neutrophil count decreased	Investigations	2024-12-27	2024-12-30	.		No	Grade 3 (Severe)	No	No reasonable possibility/ Not applicable	Reasonable possibility	Dose not Changed	Dose not Changed	Recovered/resolved
18	Headache	Nervous system disorders	2024-12-27	2025-01-15	.		No	Grade 1 (Mild)	No	No reasonable possibility/ Not applicable	No reasonable possibility/ Not applicable	Dose not Changed	Dose not Changed	Not recovered/ not resolved
18	Alanine aminotransferase increased	Investigations	2025-01-08	2025-01-12	.		No	Grade 3 (Severe)	No	No reasonable possibility/ Not applicable	Reasonable possibility	Dose not Changed	Dose not Changed	Recovering/resolving
18	Aspartate aminotransferase increased	Investigations	2025-01-08	2025-01-13	.		No	Grade 3 (Severe)	No	No reasonable possibility/ Not applicable	Reasonable possibility	Dose not Changed	Dose not Changed	Recovered/resolved
18	Alanine aminotransferase increased	Investigations	2025-01-13	2025-01-21	.		No	Grade 2 (Moderate)	No	No reasonable possibility/ Not applicable	Reasonable possibility	Dose not Changed	Dose not Changed	Recovered/resolved
18	Neutrophil count decreased	Investigations	2025-01-21	2025-01-23	.		Yes	Grade 4 (Life-threatening)	No	No reasonable possibility/ Not applicable	Reasonable possibility	Not Applicable	Not Applicable	Recovered/resolved
18	Platelet count decreased	Investigations	2025-01-21	2025-01-23	.		Yes	Grade 4 (Life-threatening)	No	No reasonable possibility/ Not applicable	Reasonable possibility	Unknown	Unknown	Recovering/resolving
18	Platelet count decreased	Investigations	2025-01-24	2025-02-02	.		Yes	Grade 3 (Severe)	No	No reasonable possibility/ Not applicable	Reasonable possibility	Unknown	Unknown	Recovering/resolving
18	Infusion related reaction	Injury, poisoning and procedural complications	2025-01-24	2025-01-24	.		Yes	Grade 2 (Moderate)	No	No reasonable possibility/ Not applicable	No reasonable possibility/ Not applicable	Dose not Changed	Dose not Changed	Recovered/resolved
18	Oedema peripheral	General disorders and administration site conditions	2025-01-18	.		Ongoing	No	Grade 2 (Moderate)	No	No reasonable possibility/ Not applicable	No reasonable possibility/ Not applicable	Dose not Changed	Dose not Changed	Not recovered/ not resolved
18	Dyspnoea	Respiratory, thoracic and mediastinal disorders	2025-01-13	2025-01-16	.		No	Grade 3 (Severe)	No	No reasonable possibility/ Not applicable	No reasonable possibility/ Not applicable	Dose not Changed	Dose not Changed	Not recovered/ not resolved
18	Pleural effusion	Respiratory, thoracic and mediastinal disorders	2025-01-16	.		Ongoing	No	Grade 2 (Moderate)	No	No reasonable possibility/ Not applicable	No reasonable possibility/ Not applicable	Dose not Changed	Dose not Changed	Not recovered/ not resolved
18	Pain	General disorders and administration site conditions	2025-01-16	.		Ongoing	No	Grade 2 (Moderate)	No	No reasonable possibility/ Not applicable	No reasonable possibility/ Not applicable	Dose not Changed	Dose not Changed	Not recovered/ not resolved

18	Vertigo	Ear and labyrinth disorders	2025-01-16	.	Ongoing	No	Grade 1 (Mild)	No	No reasonable possibility/ Not applicable	No reasonable possibility/ Not applicable	Dose not Changed	Dose not Changed	Not recovered/ not resolved
18	Body temperature increased	Investigations	2025-01-16	.	Ongoing	No	Grade 1 (Mild)	No	No reasonable possibility/ Not applicable	No reasonable possibility/ Not applicable	Dose not Changed	Dose not Changed	Not recovered/ not resolved
18	Nausea	Gastrointestinal disorders	2025-01-16	2025-02-11	.	No	Grade 1 (Mild)	No	No reasonable possibility/ Not applicable	Reasonable possibility	Dose not Changed	Dose not Changed	Recovered/resolved
18	Anaemia	Blood and lymphatic system disorders	2025-01-13	.	Ongoing	No	Grade 1 (Mild)	No	No reasonable possibility/ Not applicable	Reasonable possibility	Dose not Changed	Dose not Changed	Not recovered/ not resolved
18	Influenza	Infections and infestations	2025-01-27	2025-02-03	.	No	Grade 2 (Moderate)	No	No reasonable possibility/ Not applicable	No reasonable possibility/ Not applicable	Dose not Changed	Dose not Changed	Recovered/resolved
10	Hypokalaemia	Metabolism and nutrition disorders	2023-11-07	2023-11-09	.	No	Grade 1 (Mild)	No	No reasonable possibility/ Not applicable	No reasonable possibility/ Not applicable	Dose not Changed	Dose not Changed	Recovered/resolved
10	Diarrhoea	Gastrointestinal disorders	2023-12-01	2023-12-04	.	No	Grade 2 (Moderate)	No	Reasonable possibility	Reasonable possibility	Dose not Changed	Dose not Changed	Recovered/resolved
10	Troponin increased	Investigations	2023-11-22	.	Ongoing	No	Grade 2 (Moderate)	No	Reasonable possibility	Reasonable possibility	Dose not Changed	Dose not Changed	Not recovered/ not resolved
10	Disease progression	General disorders and administration site conditions	2023-12-05	.	Ongoing	Yes	Grade 3 (Severe)	No	No reasonable possibility/ Not applicable	No reasonable possibility/ Not applicable	Not Applicable	Not Applicable	Not recovered/ not resolved
10	Pyrexia	General disorders and administration site conditions	2023-11-24	2023-11-27	.	No	Grade 1 (Mild)	No	No reasonable possibility/ Not applicable	No reasonable possibility/ Not applicable	Dose not Changed	Dose not Changed	Recovered/resolved
12	Hepatic enzyme increased	Investigations	2024-04-24	.	Ongoing	No	Grade 3 (Severe)	No	No reasonable possibility/ Not applicable	Reasonable possibility	Dose not Changed	Dose not Changed	Not recovered/ not resolved
12	Nausea	Gastrointestinal disorders	2024-04-24	2024-04-26	.	No	Grade 1 (Mild)	No	No reasonable possibility/ Not applicable	Reasonable possibility	Dose not Changed	Dose not Changed	Recovered/resolved
12	Neutropenia	Blood and lymphatic system disorders	2024-06-10	2024-06-17	.	No	Grade 3 (Severe)	No	Reasonable possibility	Reasonable possibility	Dose not Changed	Dose not Changed	Recovered/resolved
12	Pyrexia	General disorders and administration site conditions	2024-07-10	2024-07-16	.	No	Grade 1 (Mild)	No	No reasonable possibility/ Not applicable	No reasonable possibility/ Not applicable	Dose not Changed	Dose not Changed	Recovered/resolved
12	Pneumonia	Infections and infestations	2024-07-10	2024-08-01	.	No	Grade 1 (Mild)	No	No reasonable possibility/ Not applicable	No reasonable possibility/ Not applicable	Dose not Changed	Dose not Changed	Recovered/resolved
12	Catheter site thrombosis	General disorders and administration site conditions	2024-07-29	2024-09-04	.	Yes	Grade 2 (Moderate)	No	No reasonable possibility/ Not applicable	No reasonable possibility/ Not applicable	Drug Interrupted	Dose not Changed	Recovered/resolved
12	Neutropenia	Blood and lymphatic system disorders	2024-08-19	2024-08-23	.	No	Grade 2 (Moderate)	No	Reasonable possibility	Reasonable possibility	Dose not Changed	Dose not Changed	Recovered/resolved
12	Nausea	Gastrointestinal disorders	2024-09-05	.	Ongoing	No	Grade 1 (Mild)	No	No reasonable possibility/ Not applicable	Reasonable possibility	Not Applicable	Not Applicable	Unknown
13	Atrial fibrillation	Cardiac disorders	2024-05-28	2024-05-28	.	Yes	Grade 2 (Moderate)	No	Reasonable possibility	Reasonable possibility	Drug Withdrawn	Dose not Changed	Recovered/resolved
13	Pulmonary embolism	Respiratory, thoracic and mediastinal disorders	2024-05-28	2024-08-01	.	Yes	Grade 3 (Severe)	Yes	Reasonable possibility	Reasonable possibility	Drug Interrupted	Drug Interrupted	Recovered/resolved

13	Troponin T increased	Investigations	2024-05-17	.	Ongoing	No	Grade 2 (Moderate)	No	Reasonable possibility	Reasonable possibility	Dose not Changed	Dose not Changed	Not recovered/ not resolved
13	Hepatic enzyme increased	Investigations	2024-05-15	2024-06-28	.	No	Grade 2 (Moderate)	No	No reasonable possibility/ Not applicable	Reasonable possibility	Dose not Changed	Dose not Changed	Recovered/resolved

Supplementary Table 1: AE listings as of 2025 01 27. MedDRA versions used were 27.0 eng and 27.1 eng. UPN is identical to patient numbers in table 1.

Additional pharmacokinetics data

The explanations why 1 mg/m^2, i.e. 1/3 of the single drug MTD (3 mg/m^2 tTF-NGR) is the approx. MTD level of this combination are based on striking differences in the pharmacokinetics of tTF-NGR when monotherapy is compared with the trabectedin combination. The molecular and functional integrity of the molecule and the binding and internalization kinetics of tTF-NGR:CD13 complex by activated endothelial cells (HUVEC) with presence of trabectedin are very different from the previous monotherapy data.

Particularly all values characterizing the elimination of tTF-NGR (K_{el} and t1/2) showed delayed elimination of the IMP **(Suppl. Tables 2-5)**. The terminal half-life of 1 mg/m^2 tTF-NGR after trabectedin equals the terminal half-life of 3 mg/m^2 tTF-NGR in the monotherapy **(Suppl. Table 2, 5)**. Considerably higher AUC values were measured for identical doses of tTF-NGR after trabectedin over single IMP application **(Suppl. Table 2, 3)**. Within one cycle this also leads to a higher starting concentration of tTF-NGR before the 2nd and following applications, hinting to some accumulation of tTF-NGR and/or endogenous TF released by trabectedin-damaged endothelial cells within one cycle **(Suppl. Fig. 3)**.

Non-compartment analysis of tTF-NGR, test day 1 (values up to 24 hours after start of 1st infusion)										
**Dose [mg/m²]**	**subject ID**	**C_{max}# [ng/ml]**	**t_{max}# [h]**	**$t_{1/2\ (alpha)}$ [h; 1-3 h]**	**$t_{1/2\ (alpha)}$ [h; 1-4 h]**	**$t_{1/2\ (term.)}$ [h]**	**K_{el} [1/h]**	**$AUC_{0\text{-}t\ last}$ [ng*h/ml]**	**time intervall for $t_{1/2}$ determination [h]#**	**$AUC_{0\text{-}t\ last}$/dose [h*m²*ng/ml/mg]**
**0.5**	**8/C2**	151.93	1.0	1.18	1.66	*approx. 7-10*	*0.07-0.09*	*approx. 500(-700)*	*3.0 – 24.0+*	
	**mean**									
**1.0**	**4/C1**	226.6	1.0	0.945	1.10	10.20	0.068	976.88	3.0 – 48.0*	976.88
	**4/C2**	210.5	1.0	1.05	1.38	8.76	0.079	839.17	3.0 – 24.0	839.17
	**5/C1**	294.6	1.0	0.926	2.80	6.76	0.103	931.61	3.0 – 24.0	931.61
	**5/C2**	213.8	1.0	1.19	1.46	7.97	0.087	882.78	3.0 – 24.0§	882.78
	**5/C3**	279.4	1.0	1.16	1.47	7.96	0.087	816.92	3.0 – 24.0	816.92
	**7/C1**	265.9	1.0	0.924	1.34	7.23	0.096	723.41	3.0 – 24.0	723.41
	**7/C2**	194.3	1.0	0.704	1.01	12.86	0.054	756.67	3.0 – 24.0	756.67
	**7/C3**	194.5	1.0	0.94	1.21	10.57	0.066	687.55	3.0 – 24.0	687.55
	**8/C1**	462.3	1.0	1.13	1.41	8.28	0.084	1443.91	3.0 – 24.0§	1443.91
	**mean**	**260.21**	**1.0**	**0.997**	**1.46**	**8.95**	**0.080**	**895.43**	–	**895.43**
**1.5**	**3/C1**	377.8	1.0	0.888	1.41	7.52	0.076	1033.87	3.0 – 24.0	689.25
	**mean**	**377.8**	**1.0**	**0.888**	**1.41**	**7.52**	**0.076**	**1033.87**	–	**689.25**
**2.0**	**2/C1**	541.6	1.0	0.84	0.968	9.18	0.057	1822.08	3.0 – 24.0	911.04
	**2/C2**	490.1	1.0	0.954	1.32	11.28	0.061	1926.36	3.0 – 24.0	963.18
	**mean**	**515.85**	**1.0**	**0.897**	**1.144**	**10.23**	**0.059**	**1874.22**	–	**937.11**
**3.0**	**1/C1**	883.7	1.0	1.18	1.70	9.26	0.065	2915.60	3.0 – 24.0	971.87
	**1/C2**	812.6	1.0	0.95	1.39	9.70	0.064	3054.10	3.0 – 24.0	1018.03
	**mean**	**848.15**	**1.0**	**1.065**	**1.545**	**9.48**	**0.065**	**2984.85**	–	**994.95**

Suppl. Table 2: Non-compartment analysis of tTF-NGR with a dosis of 0.5, 1.0, 1.5, 2.0, and 3.0 mg/m²,
respectively, administered to cancer patients treated with trabectedin 24 h before (TRABTRAP). Subject number identical with table 1.
[#]values obtained from plasma analysis, all other values calculated by toxicokinetic analysis; *up to 48 h/no 24-h value; [§]no 6-h value;
[+]only hypothetical 5- and 6-h values due to missing data points

	C_{max}:		AUC:	
1 mg/m^2:	Phase I: 138.10*,**	TRABTRAP: 260.21*	Phase I: 285.54*,**	TRABTRAP: 846.78*
1.5 mg/m^2	Phase I: 261.70	TRABTRAP: 377.80	Phase I: 534.69	TRABTRAP: 1033.87
2 mg/m^2:	Phase I: 393.14	TRABTRAP: 515.80	Phase I: 960.18	TRABTRAP: 1874.22$
3 mg/m^2:	Phase I: 685.24	TRABTRAP: 848.15	Phase I: 2569.71	TRABTRAP: 2984.85

Suppl. Table 3: C_{max} and AUC comparison between monotherapy with tTF-NGR at dose levels between 1 and 3 mg^2 in the phase I study with the TRABTRAP combination safety run-in cohort of a combination between trabectedin and subsequent tTF-NGR at identical dose levels. Mean values for C_{max} (ng/mL) and AUC (ng*h/ml) as measured on day 1 of a cycle.

*C_{max}: ng/mL, there were not enough values in the phase I study to make a statistical comparison for the 1 mg/m^2 dose level on day 1 (as shown). However, when comparing C_{max} values from all days measured in the multiple days protocol for the 1 mg/m^2 dose level, there were significant differences between phase I and TRABTRAP with 2-tailed p=0.0002; AUC: ng*h/ml; **mean values from phase I study updated and taken from Schliemann C. et al.: Cancers 2020, 12, 1488, doi:10.3390/cancers12061488; $TRABTRAP versus phase I gave enough values at the dose level of 2 mg/m^2 to statistically compare (2-tailed p=0.03). All other comparisons carried not enough single values in the phase I study for statistical comparison of AUC on a single dose level.

Kel (elimination constant) *,**

1 mg/m^2:	Phase I: 0.094*,**	TRABTRAP: 0.080*
1.5 mg/m^2	Phase I: 0.119	TRABTRAP: 0.076
2 mg/m^2:	Phase I: 0.139	TRABTRAP: 0.059$
3 mg/m^2:	Phase I: 0.082	TRABTRAP: 0.065

Suppl. Table 4: Kel (elimination constant) comparison between monotherapy with tTF-NGR at dose levels between 1 and 3 mg^2 in the phase I study with the TRABTRAP combination safety run-in cohort of a combination between trabectedin and subsequent tTF-NGR at identical dose levels. Mean values as measured on day 1 of a cycle.

* [1/h], **mean values from the phase I study updated and taken from Schliemann C. et al.: Cancers 2020, 12, 1488, doi:10.3390/cancers12061488; $significant difference when comparing the 2 mg/m^2 values from phase I with those from TRABTRAP (2-tailed p=0.0031), otherwise not enough values for statistical comparison.

	$t_{1/2\ alpha}$*,**		$t_{1/2\ term}$*,**	
1 mg/m^2:	Phase I: 0.73	TRABTRAP: 0.997*	Phase I: 7.34	TRABTRAP: 8.95*
1.5 mg/m^2	Phase I: 0.789	TRABTRAP: 0.888	Phase I: 5.82	TRABTRAP: 7.52
2 mg/m^2:	Phase I: 0.859	TRABTRAP: 0.897	Phase I: 5.01	TRABTRAP: 10.23$
3 mg/m^2:	Phase I: 1.06	TRABTRAP: 1.065	Phase I: 8.73	TRABTRAP: 9.48

Suppl. Table 5: $t_{1/2alpha}$ and $t_{1/2term}$ (half-life) comparison between monotherapy with tTF-NGR at dose levels between 1 and 3 mg/m^2 in the phase I study with the TRABTRAP combination safety run-in cohort of a combination between trabectedin and subsequent tTF-NGR at identical dose levels. Mean values as measured at 3 h on day 1 of a cycle.

* t1/2 [h], **mean values updated and taken from Schliemann C. et al.: Cancers 2020, 12, 1488; doi:10.3390/cancers12061488; $significant difference when comparing the 2 mg/m^2 values from phase I with those from TRABTRAP (2-tailed p=0.0031), otherwise not enough values for statistical comparison.

We subsequently have tested for cellular internalization of the tTF-NGR:CD13 complex after binding by CD13-positive endothelial cells. This would ultimately destroy tTF-NGR e.g. by intracellular proteasomes. Approx. 58% of the tTF-NGR:CD13 complex was internalized by untreated HUVEC after 3 h of incubation **(Suppl. Fig. 1 A, B, C)**. After preincubation of HUVECs with trabectedin, the presence of CD13 on HUVECs was only slightly reduced (**Suppl. Fig. 2**), but there was measurable reduction of binding of tTF-NGR to CD13, and internalization of the complex tTF-NGR:CD13 into HUVECs was considerably reduced upon the in vitro combination of trabectedin incubation followed by tTF-NGR **(Suppl. Fig. 1 D)**. This results in longer presence of tTF-NGR in the blood circulation.

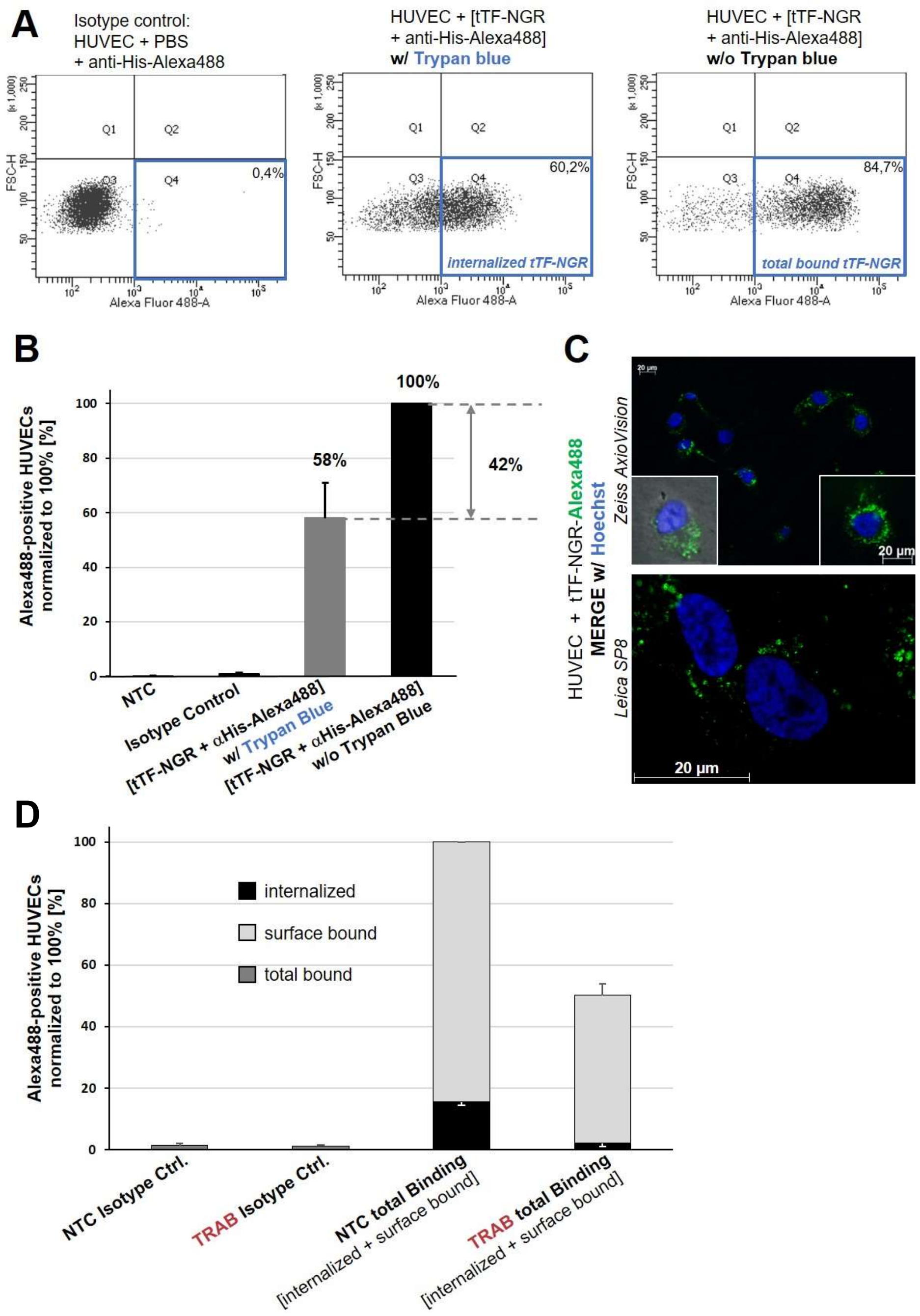
A
Isotype control:
HUVEC + PBS
+ anti-His-Alexa488
HUVEC + [tTF-NGR
+ anti-His-Alexa488]
w/ Trypan blue
HUVEC + [tTF-NGR
+ anti-His-Alexa488]
w/o Trypan blue
Q1
Q2
Q3
Q4
0,4%
60,2%
84,7%
internalized tTF-NGR
total bound tTF-NGR
FSC-H
Alexa Fluor 488-A
B
Alexa488-positive HUVECs
normalized to 100% [%]
100%
58%
42%
NTC
Isotype Control
[tTF-NGR + αHis-Alexa488]
w/ Trypan Blue
[tTF-NGR + αHis-Alexa488]
w/o Trypan Blue
C
HUVEC + tTF-NGR-Alexa488
MERGE w/ Hoechst
Zeiss AxioVision
Leica SP8
20 µm
D
Alexa488-positive HUVECs
normalized to 100% [%]
internalized
surface bound
total bound
NTC Isotype Ctrl.
TRAB Isotype Ctrl.
NTC total Binding
[internalized + surface bound]
TRAB total Binding
[internalized + surface bound]

Suppl. Fig. 1 Internalization of tTF-NGR upon binding to HUVECs and PK overview. (A) Dynamics of tTF-NGR internalization to HUVECs upon binding to CD13 in flow cytometric analysis. After 3 h-incubation of a [tTF-NGR-anti-His-Alexa488]-complex at 37°C, the ratio of bound and internalized protein was analyzed. Externally bound tTF-NGR-Alexa488 is masked when trypan blue is added, revealing the amount of internalized tTF-NGR (middle panel). Results of 2 independent experiments, normalised to 100%, are shown in **(B)** 58% of a 100% Alexa488-positive signal on HUVEC is attributed to internalized tTF-NGR (revealed by trypan blue masking) and the remaining 42% is assigned to externally bound tTF-NGR. **(C)** Directly Alexa488-labeled tTF-NGR (Abcam Alexa Fluor 488 conjugation kit [ab236553]) was incubated on HUVEC, grown on coverslips, for 3 h at 37°C. Cells where PFA-fixed, staining of the nucleus was performed by Hoechst 33342. Fluorescence microscopic captures (Zeiss Axioskop) show the localization/internalization of tTF-NGR in the cytoplasm of HUVEC cells (upper panel). A series of confocal captures (Leica SP8) confirmed the localization of tTF-NGR in the cytoplasm of HUVEC, here shown as a representative picture (lower panel). **(D)** Decrease of tTF-NGR binding to CD13 on HUVECs and tTF-NGR:CD13 internalization into HUVECs after preincubation of the cells without or with trabectedin at 10 nM for 24 h (2-tailed t-test: NTC surface bound vs. TRAB surface bound: $p = 0.0172$; NTC internalized vs. TRAB internalized: $p = 0.01$; error bars indicate standard deviations). Absolute values in A-C cannot be compared with the values in D as the experimental design of these studies and the incubation conditions were different. NTC, no treatment control.

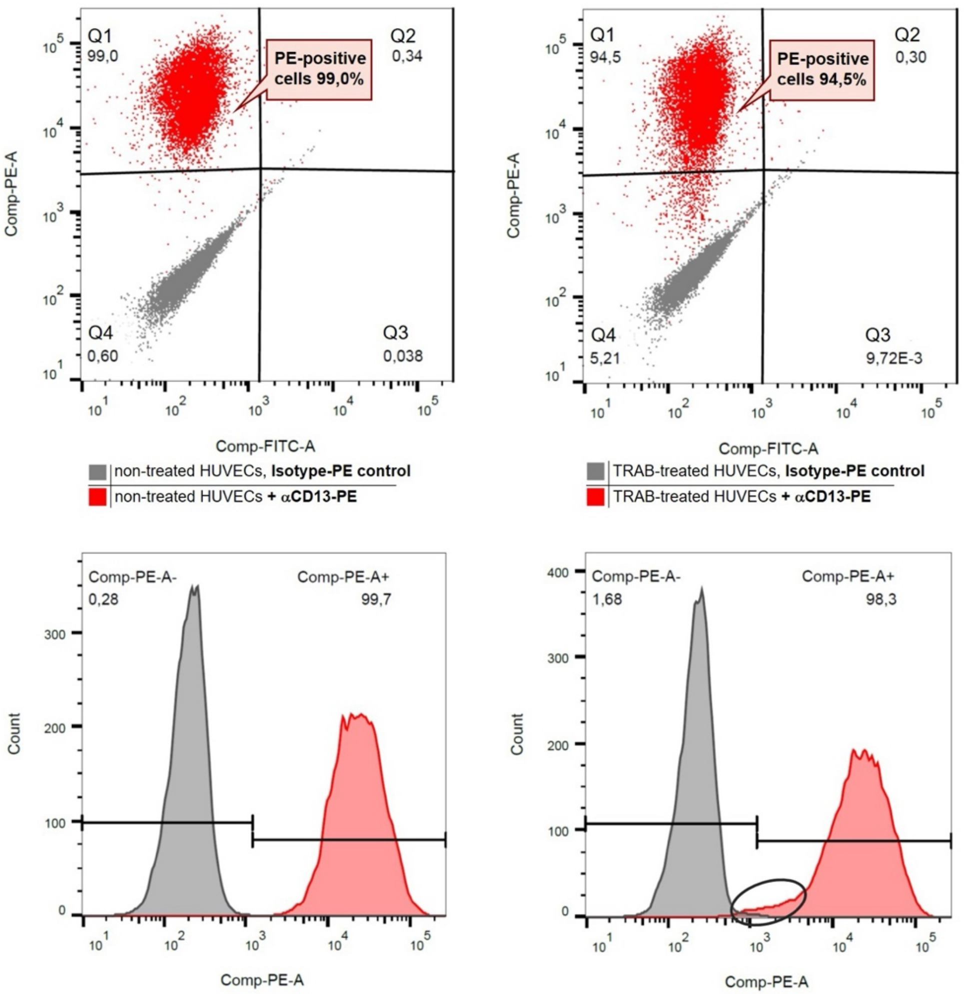

Suppl. Fig. 2 Presence of CD13 on HUCECs without or with preincubation with 10 nM trabectedin (TRAB) for 24 h, shown as dot plot (upper panel) and as histogram (lower panel). Preincubation with trabectedin resulted a slightly reduced number of CD13 positive

HUVECs and also in a reduced staining intensity of some cells (oval circle, lower panel) when compared to untreated cells. Grey represents isotype controls.

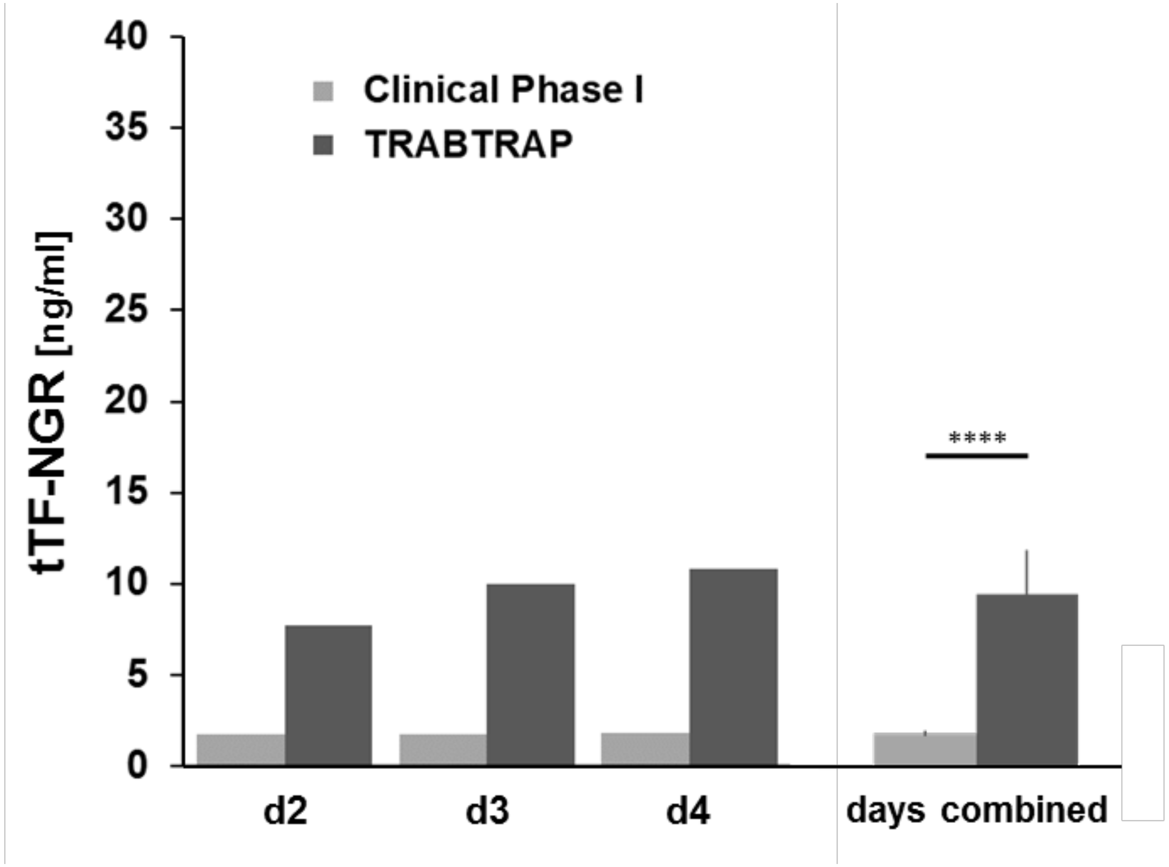

Suppl. Fig. 3 tTF-NGR concentrations before the next tTF-NGR application on days 2, 3, and 4 (cycle 1) as compared between phase I monotherapy (Ph. I) and application of tTF-NGR after trabectedin (TRABTRAP).

tTF-NGR concentrations before the following tTF-NGR application (1 mg/m^2) on the next days respectively (left, d2-d4). Whereas there was no accumulation of tTF-NGR as measured by the concentrations directly before subsequent applications in monotherapy (phase I, light grey), slightly elevated values indicated some accumulation within one cycle when tTF-NGR was applied after trabectedin (dark grey). Values of the tTF-NGR concentrations before the following tTF-NGR application on days 2, 3 and 4 combined (right, days combined; ****, $p<0.0001$, 2-tailed t-test); error bars indicate standard deviations.

Further, we compared functionality of tTF-NGR in the plasma of patients from phase I versus TRABTRAP (**Suppl. Fig. 4**). On the basis of identical protein amounts and without influence of confounders such as storage, the ability of Factor VIIa:tTF-NGR to activate Factor X to Xa was higher when trabectedin was given to the patients before. As this was dependent from time in plasma this indicated a higher degree of molecular integrity of tTF-NGR, possibly due to decreased liver protease levels after trabectedin, to lack of functional blockage by neutralizing antibodies (HAFA), and/or release of endogenous TF from endothelial cells damaged by trabectedin.

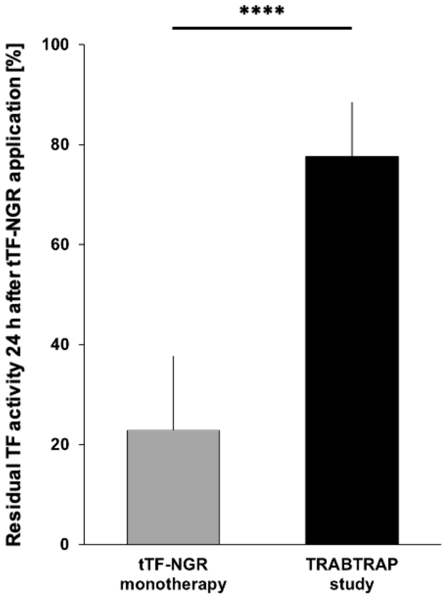

Suppl. Fig. 4 Comparison of the pro-coagulatory efficacy of tTF-NGR studying serum samples with identical amounts of tTF-NGR as observed by our validated ELISA assay for PK analysis from patients of the phase I study (tTF-NGR monotherapy) and the safety cohort of TRABTRAP, respectively, by measuring the residual TF activity 24 h after the first tTF-NGR application [%] (n=7 (tTF-NGR monotherapy), n=9 (TRABTRAP study); ****, p<0.0001, 2-tailed t-test; error bars indicate standard deviations. Mean TF-activity values from serum samples of patients which received between 0.5 and 3 mg/m² tTF-NGR.

Influence of the storage duration such as protein degradation etc. of the long-term stored patient samples of the TF monotherapy (taken from 2017 to 2019) could be excluded, since the raw TF data of both serum sample groups are in the same range (0.075 to 0.157 (monotherapy) and 0.055 to 0.160 (TRABTRAP), and even appear to be slightly higher in the monotherapy group. Thus, protein degradation processes could not be observed.

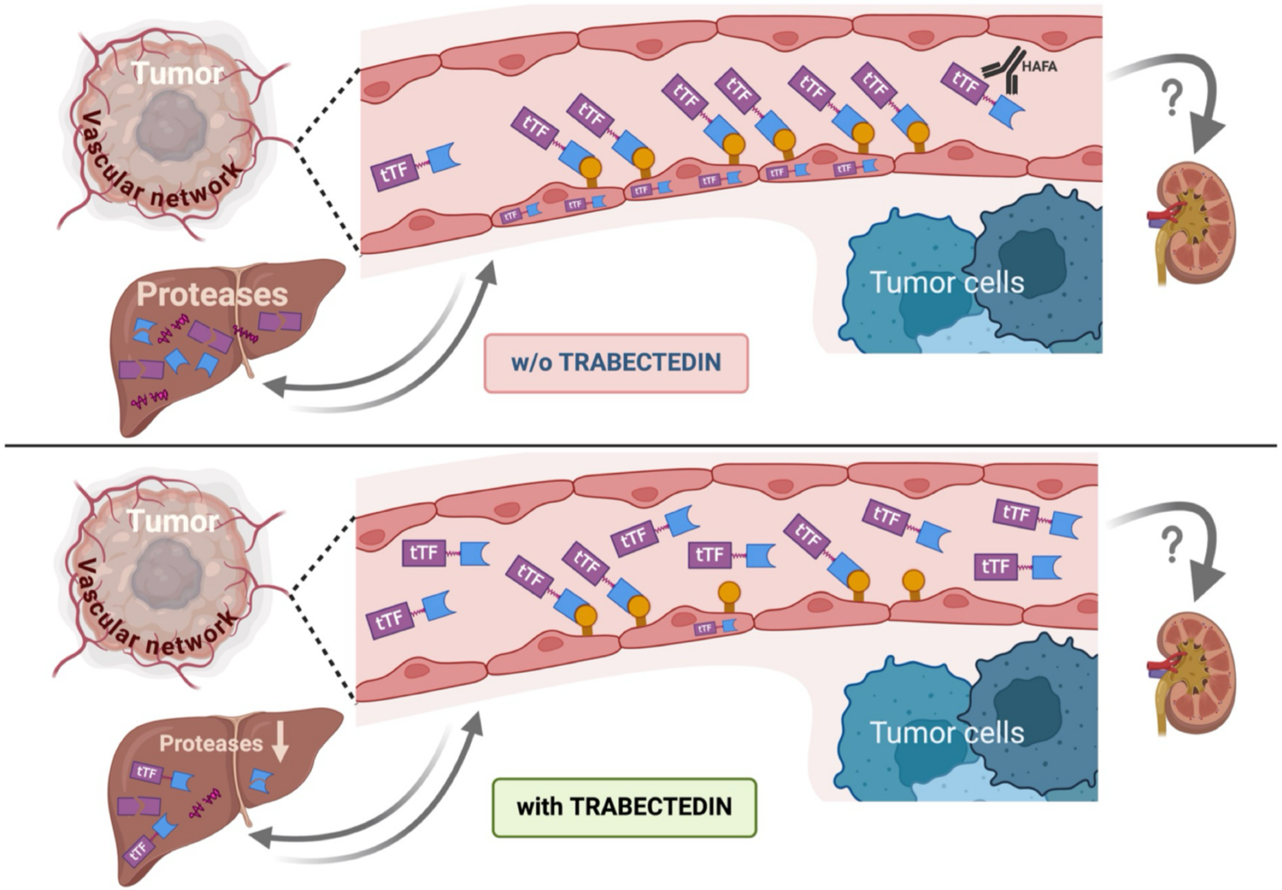

Suppl. Fig. 5 View of all observed changes in pharmacokinetics, molecular and functional integrity, binding and cellular uptake of tTF-NGR without (phase I) versus with (TRABTRAP) preceding trabectedin. HAFA, Human Anti-Fusionprotein Antibody. Created in BioRender. Berdel, A. (2025) https://BioRender.com/k55v175.

STUDY PROTOCOL

VERSION 18

01.03.2024

SPONSOR'S PROTOCOL CODE NUMBER

WWU19_0007

EUDRACT NUMBER

2020-005858-21

PHASE III STUDY COMPARING TRABECTEDIN (T) VERSUS T PLUS tTF-NGR TO ENTRAP T INSIDE THE TUMOR IN PATIENTS WITH METASTATIC AND/OR REFRACTORY SOFT TISSUE SARCOMA (STS)

Short title: **tTF-NGR Randomized Study – STS**

Acronym: TRABTRAP

Confidential

This study protocol contains intellectual property. The information in this protocol is strictly confidential. It must be only used for the conduct of the study. It must not be available to persons or institutions who are not concerned with the study. Usage for other purposes requires written approval by the Coordinating Investigator.

PHASE III STUDY COMPARING TRABECTEDIN (T) VERSUS T PLUS tTF-NGR TO ENTRAP T INSIDE THE TUMOR IN PATIENTS WITH METASTATIC AND/OR REFRACTORY SOFT TISSUE SARCOMA (STS)

Short title: tTF-NGR Randomized Study - STS

EudraCT-No.: 2020-005858-21

Sponsor:
Universität Münster, Germany
Representative: Univ.-Prof. Dr. med. Frank Ulrich Müller, Dekan
Schlossplatz 2
D-48149 Münster, Germany
Telephone[redacted]
Fax[redacted]
E-mail:[redacted]

Sponsors Study Code: WWU19_0007

Coordinating Investigator (LKP Deutschland):
Prof. Dr. med. Christoph Schliemann
University Hospital Muenster, Germany
Department of Medicine A (Hematology, Hemostaseology, Oncology)
Albert-Schweitzer-Campus 1
D-48149 Muenster, Germany
Telephone[redacted]
Fax[redacted]
E-mail[redacted]

Deputy of the Coordinating Investigator (Stellvertretender LKP Deutschland):
PD Dr. med. Torsten Kessler
University Hospital Muenster, Germany
Department of Medicine A (Hematology, Hemostaseology, Oncology)
Albert-Schweitzer-Campus 1
D-48149 Muenster, Germany
Telephone[redacted]
Fax:[redacted]
E-mail[redacted]

Protocol version 18: 2024 03 01, replacing version 17 of 2022 11 22

Study Contact: Prof. Dr. Christoph Schliemann
E-mail[redacted]
Hotline[redacted]

Signatures

Sponsor:

University Muenster represented by Univ.-Prof. Dr. med. Frank Ulrich Müller

Date: Muenster, [redacted] Signature: [redacted]

Prof. Dr. F. Müller
(Dean)

Coordinating Investigator:

Prof. Dr. med. Christoph Schliemann, Department of Medicine A, UKM

Date: Muenster, [redacted] Signature: [redacted]

Prof. Dr. C. Schliemann

Statistician:

Dr. rer. nat. Joachim Gerß, Dipl.-Stat., Institute of Biostatistics and Clinical Research, University Muenster

Date: Muenster, [redacted] Signature: [redacted]

Dr. rer. nat. J. Gerß

CONTACTS / GENERAL INFORMATION

[redacted]

[redacted]

[redacted]

[redacted]

[redacted]

Study Synopsis

**Title**	PHASE III STUDY COMPARING TRABECTEDIN (T) VERSUS T PLUS tTF-NGR TO ENTRAP T INSIDE THE TUMOR IN PATIENTS WITH METASTATIC AND/OR REFRACTORY SOFT TISSUE SARCOMA (STS)
**Short Title**	tTF-NGR Randomized Study - STS
**Acronym**	TRABTRAP
**Sponsor´s Protocol Code Number**	WWU19_0007
**EudraCT-No.**	2020-005858-21
**Rationale**	tTF-NGR targets CD13 present in tumor-associated vasculature and on tumor cells of the majority of STS tissue samples examined; preclinical data on combination of tTF-NGR with anthracyclines and trabectedin; low competition of targeted or immune therapy in soft tissue sarcoma (STS)
**Sponsor**	Universität Münster
**Coordinating Investigator**	Prof. Dr. med. Christoph Schliemann<br>Department of Medicine A (Hematology, Hemostaseology, Oncology)<br>University Hospital Muenster, Germany<br>Albert-Schweitzer-Campus 1<br>D-48149 Muenster, Germany<br>Telephone[redacted]<br>Fax:[redacted]<br>E-mail[redacted]
**Investigational Medicinal Product**	Patients will receive a dose of the Investigational Medicinal Product (IMP) tTF-NGR determined to be safe within the safety run-in cohort of the study as 1-hour rate-controlled infusion (port or central venous access, 0.9 % NaCl ad 100 mL) per day for 4 or lower number of consecutive days following each trabectedin cycle (within 1 hour interval between end of trabectedin infusion and tTF-NGR: e.g.: trabectedin on monday 8 am to tuesday 8 am followed by tTF-NGR on tuesday latest 9 am and on the following days, q d 22 x until disease progression or contraindications against further application.
**Indication**	Unresectable or metastatic STS after failure of anthracycline-containing first line treatment or with contraindications to these drugs; CD13 positivity in central histology (grade >/= 1+)
**Inclusion/exclusion criteria**	For inclusion and exclusion criteria see chapter 6.3.
**Primary objective and endpoints**	The primary objective of the trial is to evaluate whether tTF-NGR in combination with standard trabectedin chemotherapy given for unresectable or metastatic soft tissue sarcoma after failure of anthracycline-containing first line therapy or with contraindications to

	these drugs prolongs progression-free survival, as compared with trabectedin alone.<br>The following efficacy endpoint (for the randomized phase III part) will be considered:<br>- Progression-free survival (PFS) according to iRECIST (Seymour L, Lancet Oncol. 2017) as judged by central radiology in a blinded fashion
**Secondary objectives and endpoints**	The secondary objective of the trial is to evaluate the efficacy of tTF-NGR in combination with standard trabectedin chemotherapy given for unresectable or metastatic STS after failure of anthracycline-containing first line therapy or with contraindications to these drugs with respect to the response rate and overall survival as well as to assess the safety profile of tTF-NGR combined with trabectedin.<br><br>To assess the *efficacy*, the following measurements will be considered:<br>- Overall response rate (ORR, consisting of CR and PR)<br>- Disease control rate (DCR, consisting of CR, PR, and stable disease (SD) for $\geq$18 weeks)<br>- Median progression-free survival (mPFS)<br>- Median overall survival (mOS)<br>- Overall survival (OS) rate at 12 and 18 months<br><br>To assess the *safety profile* of tTF-NGR combined with trabectedin (for the safety run-in cohort and the main randomized phase III parts), the following safety endpoints will be considered:<br>- Adverse Events (AEs) assessment based on CTCAE v.5.0.<br>- Standard laboratory parameters and pharmacokinetics<br>- Physical examination findings including assessment of vital signs<br>- Patient reported outcomes (PRO)
**Study design**	Open label, randomized, controlled study in subjects with metastatic or refractory soft tissue sarcoma. Approx. 150 patients will be screened, 126 evaluable patients are enrolled and parallel assigned in a 1:1 fashion to one of two different arms, as outlined below. Randomization will be stratified into CD13+ grades 1 and 2 versus CD13+ grade 3 and number of chemotherapy regimen before entry on trial: 1 versus >1 .<br><br>*Safety run-in part:*<br>Before the randomized phase III part of the study, there will be a *safety cohort* of a minimum of 6 patients obtaining at least 2 cycles each of the combination outlined in arm 2 (see below) to confirm safety of this combination (1.5 mg/m^2 trabectedin plus a starting dose of 3 mg/m^2 tTF-NGR). The patients will be treated in-house and in sequence. In case of dose-limiting toxicity (DLT) in one patient of this cohort, a dose-modification protocol for tTF-NGR to 2 mg/m^2 is planned, and in case of further tolerability problems in one patient further deescalations in 0.5 mg/m^2 steps of tTF-NGR and/or by a reduction of application days are planned. The safe dose is then transferred to the randomized phase III part of the study. The final dose and the final number of application

	days of tTF-NGR in this combination has to be applied to 6 patients with 2 cycles each without DLT to be established as safe. The randomized part of the study will be opened after judgement of the safety in the safety cohort by the DSMB, Ethics Committee and National Competent Authority (PEI).<br><br>*Randomized Phase III part:*<br>ARM 1:<br>Trabectedin 1.5 mg/m^2 as a 24-hour central intravenous (IV) infusion on day 1, q d 22 x until disease progression or contraindications against further application.<br><br>ARM 2:<br>Patients will receive standard trabectedin according to arm 1 plus the safe dose according to safety run-in part of tTF-NGR (1-hour rate-controlled infusion, port or central venous access, 0.9 % NaCl ad 100 mL) per day for 4 or lower number of consecutive days following each trabectedin cycle (within 1 hour interval between end of trabectedin infusion and tTF-NGR: e.g.: trabectedin on monday 8 am to tuesday 8 am followed by tTF-NGR on tuesday 9 am and on the following days, q d 22 x until disease progression or contraindications against further application.<br><br>As the evaluation of the study results is based on an intention-to-treat analysis, all patients after randomization will be part of the efficacy population as evaluated by central iRECIST evaluation after end of study.<br>Therapy in both arms can be given on an out-patient basis. All patients receive best supportive care (BSC) according to institutional guidelines. Anti-cancer activity (iRECIST modification) will be assessed (clinically and imaging) at week 9 and then every 9 weeks (adjusted to cycle length of 3 weeks) until confirmed progression by iRECIST (iCPD). Decision on application of next cycle at week 9 will be clinical. Imaging and clinical based decision will follow. Transfer of pseudonymized imaging pictures (CD, DVD) and imaging results of a patient to the study center has to be performed after each imaging time point.<br><br>Safety assessment will be performed on an ongoing basis during study participation, including standard laboratory assessments. The incidence of AEs will be summarized by severity in all patients with at least one study drug intake.
**Planned study dates**	Start of study / recruitment: Initial planned date, Q4 2021<br>End of Trial: corresponds to the last visit of the last patient undergoing the trial (LPLV).<br>Tentative enrolment duration: about 60 months from the first patient of the first visit (FPFV) in the randomized phase to the last patient first visit (LPFV).

**Number of sites, countries and patients**	Multicenter, up to 14 active study sites in Germany and Austria.<br><br>At least 6 patients are treated in the safety part, 126 patients will be randomized in a 1:1 ratio to receive open label Trabectedin (Arm 1) or Trabectedin plus tTF-NGR (Arm 2) in the randomized phase III part.
**Target population**	Patients (18-75 years) with advanced or metastatic soft-tissue sarcoma after failure of anthracycline-containing first line therapy or with contraindications to these drugs.
**ARM 1**	Standard chemotherapy with Trabectidin (in-label)
**ARM 2** Test product	tTF-NGR added to standard Trabectedin
**Severity grading**	Hematological and chemical laboratory tests, and AEs will be graded based on CTCAE v. 5.0.
**Duration of Treatment**	Patients will be treated in repeated cycles until definite disease progression (iRECIST; Seymour L, Lancet Oncol. 2017) in the absence of other withdrawal criteria, and as long as neither patient nor investigator requests treatment discontinuation.
**Tumor Assessments**	Detailed tumor assessment visits (clinical and laboratory examinations) after start of therapy are performed before treatment start, at week 9 (+/- 1 week), followed by every 9 weeks (+/- 1 week, independent from cycle length of 3 weeks) thereafter. Post-study treatment (treatment with another anti-cancer agent) is according to investigator´s choice, but is recorded in the eCRF. At iCPD (confirmed at next tumor assessment after iUPD) tumor assessments will end, but time of death will be recorded for estimation of OS. In case EOT is not determined by iCPD, tumor assessments will go on after EOT until iCPD. Survival status will be collected also for patients withdrawn from the study until death. Transfer of pseudonymized imaging pictures (CD, DVD) and imaging results of a patient to the study center has to be performed after each imaging time point.
**Statistical analysis**	Safety data of the safety run-in part of the study will be evaluated using descriptive statistical methods.<br><br>In the primary statistical analysis of the randomized phase III part, the primary endpoint progression-free survival (PFS) according to iRECIST as judged by central blinded radiology will be compared between the two randomized treatment groups (Arm 1 versus Arm 2). The primary statistical analysis will include all randomized patients (full analysis set) and will be performed according to the intention-to-treat principle. A stratified log-rank test with stratification according to the randomization will be applied (two-sided significance level 5%, power 80%) that provides confirmatory statistical evidence.<br><br>The timing of the study will be event-driven. The primary statistical analysis will be performed at the time when 106 events in terms of PFS have been observed in total across both treatment groups. This required number of events results from the following sample size calculation. Based on the observed PFS in previous trials, PFS is

	expected to be exponential, and the expected median PFS is 4.6 months in the control arm (Arm 1) and 8 months in the experimental arm (Arm 2). The corresponding expected hazard ratio is HR=0.575. In a 36 months enrolment period with a rate of 3-4 patients per month, 126 patients will be recruited and randomized. The drop-out process after randomization is expected to be exponential with an up to 25% cumulative drop-out rate at month 24 (competing with the survival process). Follow-up after the last randomized patient is planned to be 12 months, after which it is expected that the required number of 106 events in terms of PFS will have occurred.<br>The statistical analysis of pre-specified secondary endpoints will be performed with descriptive and inferential statistical methods. Prespecified subgroup analyses will be performed with respect to stratification, L-sarcoma vs. others, FNCLCC grade (2 vs. 3), and ECOG performance status (ECOG PS 0 vs. PS 1). Further exploratory analyses will be performed for other relevant treatment variables (e.g. sex, age, number of treatment cycles).<br>Safety data will be evaluated and summarized descriptively.
**Withdrawal and patient replacement criteria**	The patient can withdraw consent for participation in the study at any time without disadvantages for further treatment or prejudice by the therapeutic team. The investigator can withdraw a patient if, in his or her clinical judgment, it is in the best interest of the patient or if the patient cannot comply with the protocol.

TABLE OF CONTENTS

1 LIST OF ABBREVIATIONS AND DEFINITION OF TERMS 14

2 ETHICAL AND REGULATORY REQUIREMENTS 15

2.1 Approval of Study Protocol 15

2.2 Ethical Conduct and Good Clinical Practice 16

2.3 Informed Consent of Patient and Confidentiality 16

2.3.1 Informed Consent of Patient 16

2.3.2 Patient Confidentiality 17

2.4 Liability and Insurance 17

3 INVESTIGATORS AND STUDY ADMINISTRATIVE STRUCTURE 17

4 INTRODUCTION 18

4.1 Background 18

4.1.1 Metastatic Soft tissue sarcoma 18

4.1.2 tTF-NGR: Tissue Factor targeted to CD13 in tumor vasculature 18

4.1.3 Targeted delivery of tTF-NGR and functional imaging 24

4.1.4 Clinical Experience with tTF-NGR 28

4.1.5 Trabectedin 33

4.2 Rationale for the study & benefit-risk assessment 34

5 STUDY ENDPOINTS AND OBJECTIVES 35

5.1 Primary endpoints and objectives 35

5.2 Secondary endpoints and objectives (for the phase III randomized part of the study) **35**

6 OVERALL STUDY DESIGN 36

6.1 Overall Study Design and Plan 36

6.1.1 Description 36

6.1.2 Planned Duration of Study 40

6.1.3 Premature Discontinuation of the Study 41

6.1.4 Stopping/Pausing Rules 41

6.2 Discussion of Overall Study Design 42

6.3 Selection of Study Population 42

6.3.1 Inclusion Criteria 43

6.3.2 Exclusion Criteria 44

6.3.3 Subject Withdrawal from further treatment (Early Termination) 46

6.3.4 Patient replacement criteria 46

6.3.5 Patient screening, enrollment and identification 46
6.4 IMP or Study Drug 46
6.4.1 IMP Composition 46
6.4.2 IMP Labeling, Storage and Packaging – tTF-NGR 47
6.4.3 Identity of IMP 47
6.4.5 Other concomitant therapies 48
6.4.6 IMP Compliance 49
6.5 Procedures and Variables 51
6.5.1 Measurements Assessed 51
6.5.3 Primary variables 63
6.5.4 Secondary variables 64
6.5.5 Safety variables 64
6.5.6 Efficacy variables 74
6.5.7 Other variables and procedures 76
6.6 Study Monitoring, Auditis, Inspections and Documentation 78
6.6.1 Study Monitoring 78
6.6.2 Source Data Verification 78
6.7 Data Management and DSMB 79
6.7.1 Data Handling Procedures 79
6.7.2 Record Retention 79
6.7.3 Data Protection 79
6.7.4 DSMB 80
6.8 Changes in the Conduct of the Study or Planned Analysis 80
6.9 Statistical Methods and Determination of Sample Size 80
6.9.1 Statistical Methods 80
6.9.2 Determination of Sample Size 83
6.10 Documentation of Study Findings 83
6.11 Record Retention Period 84
6.12 Use of Information and Publication 84
7 REFERENCE LIST 85
8 INVESTIGATOR AGREEMENT 89

Attachments

1 LIST OF ABBREVIATIONS AND DEFINITION OF TERMS

ADL	Activities of Daily Living
AE	Adverse Event
alpha 2 AP	Alpha2-antiplasmin
ALT	Alanine Transaminase (SGPT)
APN	aminopeptidase N (= CD13)
AR	Adverse Reaction
AST	Aspartate Transferase (SGOT)
AT III	Antithrombin III
CD13	Cluster of Differentiation 13 (= aminopeptidase N, APN)
CD51/CD61	integrin alpha$_v$beta$_3$
CEUS	Contrast-Enhanced Ultrasound
CNS	central nervous system
CR	Complete Response
CRF	Case Report Form
CTC	Common Toxicity Criteria
CTCAE	Common Terminology Criteria for Adverse Events
D	day
DIC	disseminated intravascular coagulation
DIMDI	Deutsches Institut für Medizinische Dokumentation und Information
DLT	Dose-Limiting Toxicity
DSMB	Data and safety monitoring board
DSUR	Development Safety Update Report
E.coli	*Escherichia coli*
EC	endothelial cells
ECG	Electrocardiography
eCRF	electronic Case Report Form
EDC	Electronic Data Capture
EOT	End of Study Treatment
EudraCT	EU Database on Clinical Trials
FBC	Full Blood Count
FDA	Food & Drug Administration
FPFV	First Patient First Visit
GCP	Good Clinical Practice
h	hour
HAFA	human anti-human fusionprotein (tTF-NGR) antibody
HPLC	High Performance Liquid Chromatography
IB	Investigators Brochure
ICH	International Council for Harmonisation of Technical Requirements for Pharmaceuticals for Human Use
ICU	Intensive Care Unit
IMP	investigational medical product
IMPD	Investigational Medicinal Product Dossier
ISF	Investigator Site File
iv	intravenously
LDH	Lactate dehydrogenase
LPFV	Last Patient First Visit
LPLV	Last Patient Last Visit
LKP	Leiter der Klinischen Prüfung
MRI	Magnetic Resonance Imaging
MTD	Maximum Tolerated Dose
nL	nanoliter
OTS	Other Type Sarcoma
PCHE	Pseudocholinesterase
PD	Progressive Disease
PR	Partial Response
PRO	Patient Reported Outcomes
pt	patient
PTT	Partial thromboplastin time
RECIST	Response Evaluation Criteria In Solid Tumors

SAE	Serious Adverse Event
SD	Stable Disease
SGOT	Serum Glutamic Oxaloacetic Transaminase
SGPT	Serum Glutamic-Pyruvic Transaminase
STS	Soft Tissue Sarcoma
SUSAR	Serious Unexpected Serious Adverse Reaction
TEC	tumor endothelial cells
TMF	Trial Master File
tTF-NGR	tTF: truncated tissue factor; NGR: peptide GNGRAHA
TZ	Thrombinzeit (engl.: thrombin clotting time (TCT))
UKM	University Hospital Muenster
ULN	upper normal limit
VEGF	vascular endothelial growth factor
w	week
ZKS	Zentrum für Klinische Studien

Sponsor: An individual, company, institution, or organization, which takes responsibility for the initiation, management, and/or financing of a clinical trial.

Clinical Research Associate or Study Monitor: Sponsor's designated representative responsible of managing, supervising, and monitoring the overall conduct of the trial.

2 ETHICAL AND REGULATORY REQUIREMENTS

2.1 Approval of Study Protocol

The present study will not be started before the appropriate competent Ethics committees have given a favorable opinion and approval by the competent federal authorities has been obtained. Besides, for each participating study site a favorable opinion of the respective Ethics committee is required.

The Sponsor will provide the competent Ethics Committees (EC) with all appropriate documents necessary for the approval of the trial, including the informed consent form (ICF), according to the national regulations. In addition, all obligatory documents will be submitted to the competent authorities, such as the Paul Ehrlich-Institute (PEI) in compliance with national regulations. The trial will not be initiated until appropriate EC approval of the protocol and the informed consent document. Prior to enrollment, the sponsor and the investigator must exchange written confirmation that their ethical and legal responsibilities have been observed. According to German drug law §67, the investigator is required to notify the local surveillance authority about the beginning, the end and a premature termination of the study, about any substantial amendments and if the study is on hold. Additionally, the investigator is required to notify the competent federal authority of the beginning of the study. These notifications will be performed by an appointee of the sponsor (GCP-V, §12(3)). The EC and the competent authority must be informed of protocol amendments in accordance with national legal requirements and approval obtained as necessary before implementation. Appropriate reports on the progress of the study will be made to the EC and the sponsor by the

investigator in accordance with applicable governmental regulations and in agreement with policy established by the sponsor.

2.2 Ethical Conduct and Good Clinical Practice

This trial will be conducted in accordance with the ethical principles that have their origin in the Declaration of Helsinki, and that are consistent with Good Clinical Practice (GCP), and the applicable regulatory requirement(s) for the country in which the trial is conducted according to International Conference on Harmonization (ICH) guidelines, and applicable Standard Operating Procedures (SOPs). Specifically, this trial is based on adequately performed laboratory and animal experimentation; the trial will be conducted by scientifically and medically qualified persons; the benefits of the study are in proportion to the risks; the rights and welfare of the patients will be respected; the physicians conducting the trial do not find the hazards to outweigh the potential benefits; each patient, or where applicable, each patient's legally acceptable representative(s) will give his or her written informed consent before any protocol-driven tests or evaluations are performed. A copy of the ICH GCP guidelines and of the Declaration of Helsinki will be included in the Trial Master File (TMF).

2.3 Informed Consent of Patient and Confidentiality

2.3.1 Informed Consent of Patient

The investigator is responsible to obtain informed consent in adherence to GCP and according to applicable regulations prior to entering the patient into the trial.

The information about the trial must be given orally and in an understandable form. Written information about the trial will also be provided. In addition to the explanation of the trial and of patient's legal rights the information should comprise that access to original medical records and processing of coded personal information must be authorized. The informed consent discussion must be conducted by a person who is qualified according to applicable local regulations. The patient should have the opportunity to inquire about details of the trial and to consider participation.

The informed consent form (ICF) must be signed and dated by the patient and must be countersigned by the person who conducted the informed consent discussion (according to national laws and ICH-GCP).

If a patient is unable to read or write, oral consent in the presence of an impartial witness is possible, if this is permitted by local legislation. In this case, the witness has to be present during the meeting in which the significance of the informed consent will be orally explained. After the informed consent discussion and after the patient has orally consented to participate in the clinical trial the witness should sign and personally date the consent form to attest that information concerning the clinical trial and the patient's rights was accurately explained to, and apparently understood by the patient and that informed consent was given freely.

The investigator will provide a copy of the signed informed consent to the patient and will maintain the original in the Investigator Site file (ISF).

The written ICF and any other written information to be provided to patients should be revised whenever important new information becomes available that may be relevant to the patient's consent. Any revised written informed consent form, and written information should receive EC's approval as required by national regulation, before use.

The patient should be informed in a timely manner if new information becomes available that may affect the decision to participate in the clinical trial. The communication of this information should be documented.

2.3.2 Patient Confidentiality

Patient names will not be supplied to the sponsor or third party. Only the patient numbers and patient identification codes will be recorded in the electronic Case Report Form (eCRF), and if a patient's name appears on any other document (e.g., pathologist report), it will be obliterated before the copy of the document is supplied to the sponsor. Study findings stored on a computer will be subjected to local data protection laws. The patient, or where applicable, the patient's legally acceptable representative(s) will be informed that representatives of the sponsor, EC, or regulatory authorities may inspect their medical records to verify the information collected, and that all personal information made available for inspection will be handled in the strictest confidence. The investigator or designee will maintain a site list of patient numbers and patient identification codes to enable records to be found at a later date.

2.4 Liability and Insurance

The involved parties will be insured, in accordance with applicable laws and regulations, against financial loss resulting from personal injury and/or other damages, which may arise as a consequence of this study. Appropriate test person insurance will be concluded by the Sponsor. Sponsor will maintain sufficient general liability insurance.

3 INVESTIGATORS AND STUDY ADMINISTRATIVE STRUCTURE

The trial will be managed and monitored by the Study Office and the Zentrum für Klinische Studien (ZKS) of the Faculty of Medicine at the University Muenster. Study Monitors will monitor the site on a periodic basis and perform verification of source documentation for each patient. Investigators at each study center will be responsible for the timely reporting of serious adverse events (SAEs). The sponsor's Safety Desk at the ZKS Münster will be responsible for organizing SAE management including sponsor's reporting obligations.

Prof. Dr. med. Christoph Schliemann is Coordinating Investigator for this multicenter study (LKP Deutschland), PD Dr. med. Torsten Kessler is Deputy Coordinating Investigator.

4 INTRODUCTION

4.1 Background

4.1.1 Metastatic Soft tissue sarcoma

Soft Tissue Sarcoma (STS) is a rare group of heterogeneous mesenchymal cancers originating from connective tissue. STS is made up of more than 100 distinct subtypes, which collectively account for 1% of all adult cancers and the incidence of STS in Europa sarcoma ranges between 2–5/100,000/year (1). They occur anywhere in the organism, common sites are extremities, trunk, retroperitoneum and head/neck. Exact histopathological diagnosis and grading as well as tumor staging by imaging is important, since it influences multidisciplinary therapy, best carried out in experienced reference centers. Due to the heterogeneity and the rarity of the disease, diagnosis is often late.

Surgery is the first line of treatment for early stage and localized disease. However, distant metastases occur in many patients, especially in those with high-grade tumors. For patients with unresectable STS, chemotherapy is the standard of treatment (2,3) and the therapeutic approach is multidisciplinary. In cases with metastatic disease, the therapeutic aim often is restricted to palliation and/or prolongation of progression-free survival time instead of cure. First line systemic therapy is based on doxorubicin alone or in several combinations such as doxorubicin and ifosfamide. The combination of gemcitabine and docetaxel seems to be equally effective as doxorubicin, but induces more severe hematotoxicity and is sometimes reserved for second line therapy. Besides conventional chemotherapy, trabectedin, pazopanib, and eribulin are alternatives for second- and further line therapy, eribulin only in liposarcoma. However, overall survival of patients with advanced STS in general remains poor. Thus, there is an unmet medical need for new therapeutic targets and agents in this group of diseases.

4.1.2 tTF-NGR: Tissue Factor targeted to CD13 in tumor vasculature

tTF-NGR is a recombinant fusion protein made of two moieties:

- tTF (truncated tissue factor) is the main human initiator of extrinsic coagulation lacking the transmembrane domain at the C-terminus.
- GNGRAHA is the targeting peptide binding to CD13 and fused to the C-terminal of tTF instead of the transmembrane domain.
- tTF-NGR is the fusion protein of tTF- and GNGRAHA at the C-terminus prepared as a recombinant protein in *E. coli*.

The details of the preclinical and clinical phase I and TRABTRAP safety cohort data for tTF-NGR are summarized in the Investigator Brochure (IB) accompanying this study protocol.

The *rationale* for this study and *results concerning tTF-NGR* directly pertinent to this

study are summarized in the following:

Growth and spread of tumors is dependent from intratumoral neovascularization to deliver nutrients and oxygen and remove metabolic waste products. Tumor endothelial cells (TEC) are essential for building tumor vessels and among other cell types such as fibroblasts and some myeloid and immune cells belong to the tumor-supportive stroma. TEC express novel targets absent from resting endothelial cells in the mature vasculature of the organism. Some of these TEC targets are clinically relevant for *anti-angiogenic therapy* against cancer, which led to numerous drugs approved for cancer treatment (e.g. bevacizumab (anti-VEGF moAB), aflibercept (VEGF trap), ramucirumab (anti-VEGF-R moAB), Sunitinib, Sorafenib, Pazopanib (examples for tyrosine kinase inhibitors for TEC tyrosine kinase receptors)). Therapeutic activity of these drugs is limited to few months increase of survival and occurrence of resistance limits the approach.

Conceptually different from anti-angiogenic treatment is the *anti-vascular approach*. Antivascular drugs do not only interfere with vessel formation, but aim to destroy neo-vessels already present in the tumors. In this class no drug is approved yet and few (bavituxumab (anti-phosphatidylserine moAB), ombrabulin (vascular disruption)) are in clinical studies.

Among antivascular compounds are bifunctional molecules targeting a TEC specific structure on TECs with one moiety and carrying an antitumor payload as or with the second moiety. The hope for this class of *vascular targeting* compounds is to target more than one single tumor entity via the common tumor vasculature. Analog drugs targeting tumor cells of only one histological entitiy are already approved for treatment of specific tumors, such as moABs to tumor cell associated molecules carrying toxins (brentuximab vedotin (CD30+ Hodgkins disease), trastuzumab emtansine (HER2+ breast cancer)).

We have developed a new class of bifunctional fusion proteins carrying an essential pro-coagulatory molecule – tissue factor (TF) – via C-terminal peptides into tumor vessels to induce *selective tumor vascular infarction*. To this end we have removed the non-specific membrane anchor of TF and replaced it by peptides including e.g. the CD13-binding NGR motif. CD13 is an aminopeptidase selectively occurring on stimulated and growing EC, as on TEC with only limited expression on normal tissue (http://www.proteinatlas.org/ENSG00000166825-ANPEP/tissue). In some normal tissues such as small bile ducts, expression of CD13 furthermore is present, but does not disturb application of CD13-targeted TF, since this molecule is active only in a coagulation-competent location, such as in blood vessels, and not elsewhere. With this molecule (Figure 1) we target and accumulate TF-activity to tumor vasculature and induce tumor vascular thrombosis and infarction leading to tumor cell death (Figure 2).

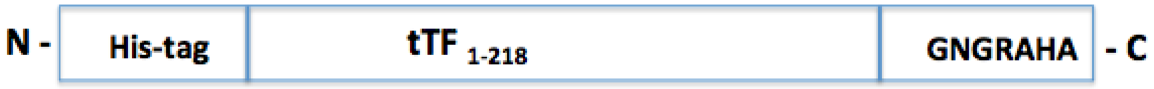

Figure 1: Schematic structure of the lead compound **tTF-NGR** with a N-terminal His-tag for detection and purification, full-length TF, and the targeting peptide with the NGR-motif at the C-terminus.

We have cloned and biotechnologically produced a series of >10 of those retargeted TFs with different target molecules on TEC and also on tumor vessel pericytes and tested them *in vitro* and *in vivo* for essential therapeutic properties (4-21), such as:

- pro-coagulatory activity in comparison to TF in a factor X/factor Xa assay,
- specific binding to their respective target molecules on stimulated EC or pericytes
- *in vivo* intratumoral accumulation with different imaging techniques,
- *in vivo* induced intratumoral activation of coagulation,
- tumor vascular occlusion and inhibition of tumor vessel blood flow,
- pharmacodynamic properties with *in vivo* therapeutic antitumor activity in xenotransplants, rodent and non-rodent toxicology.

The *lead compound*, tTF-NGR in comparison to other molecules from this series showed the most robust *in vitro* and *in vivo* activity. tTF-NGR retains complete procoagulatory activity, specifically binds to CD13 on growing EC, it leads to tumor vessel infarction and occlusion with resulting tumor growth inhibition and regression (Figures 3). Preclinical therapeutic activity of tTF-NGR was independent of tumor histology (e.g. melanoma, lung, breast, sarcoma, glioblastoma). Repeated rounds of treatment showed no resistance development. Details of these results are published (5-21). In addition, combination of tTF-NGR with cytotoxic drugs such as doxorubicin showed synergistic therapeutic activity *in vivo*, with complete abrogation of tumor growth in all animals treated for prolonged times of observation (Figure 4; ref. 17). This synergistic effect occurs partly because tumor vascular occlusion by tTF-NGR prolonged intratumoral accumulation of the cytotoxic with resulting higher apoptotic efficacy.

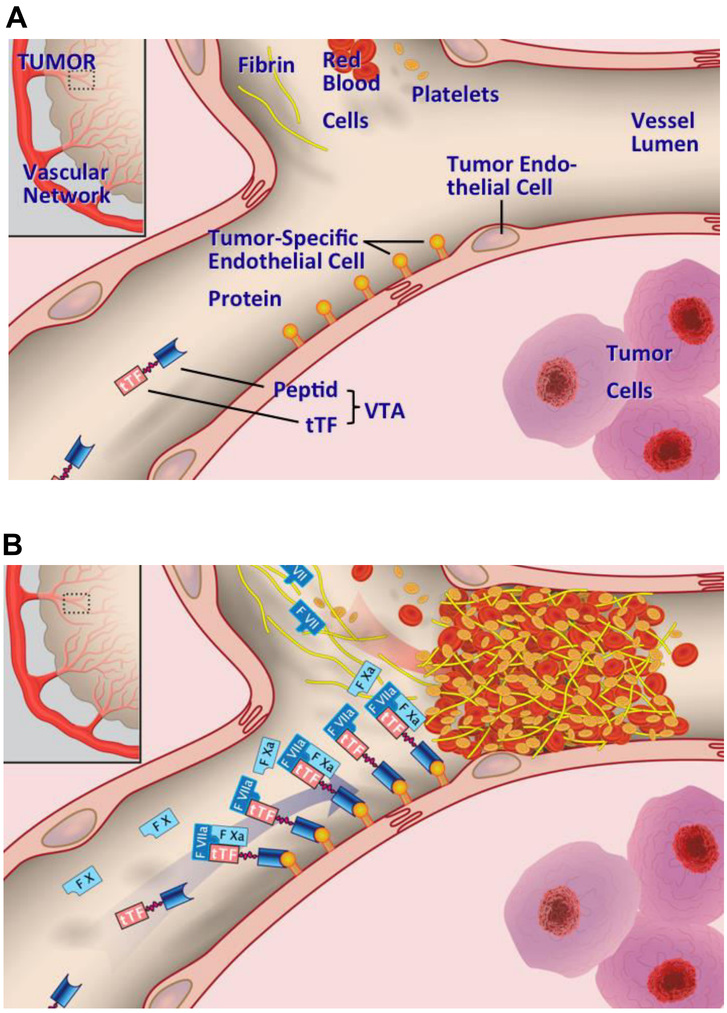

Figure 2: A, Components of the tumor vascular infarction at the onset of therapy and **B**, during tumor vascular clotting. FXa, factor X activated (light blue), FVIIa, factor VII activated (blue)

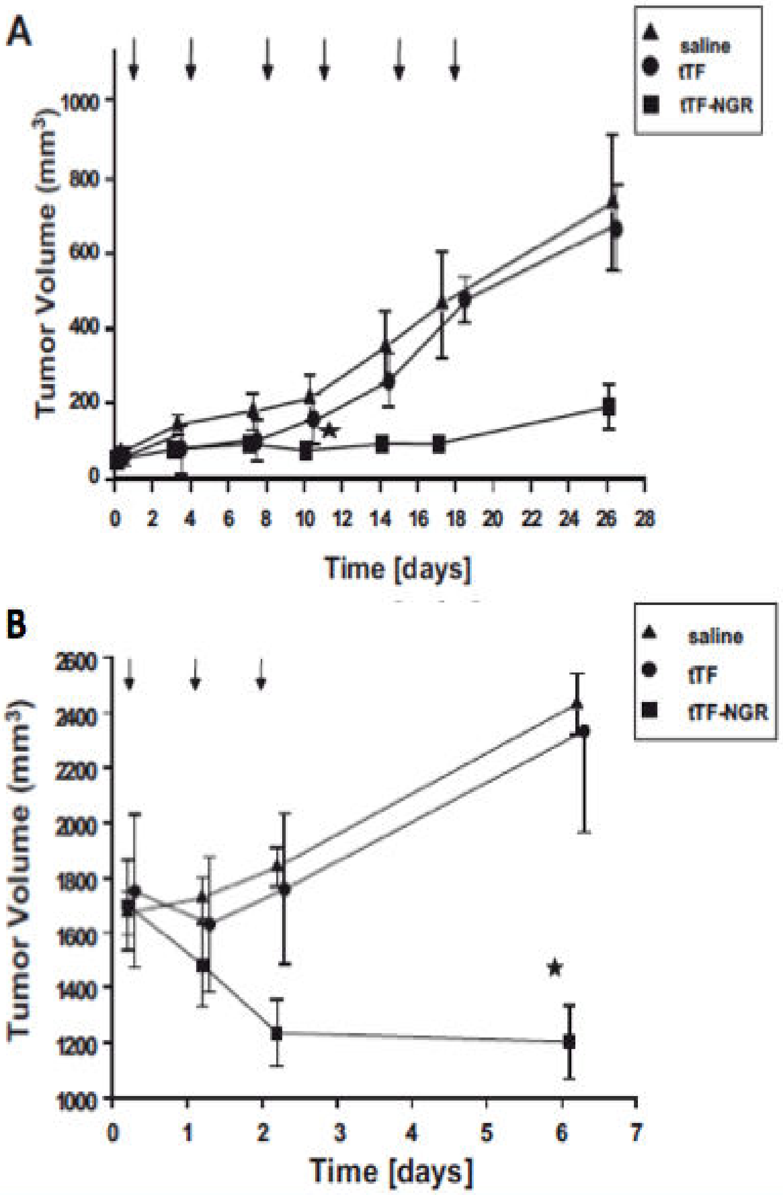

Figure 3: A. Examples of growth inhibition of human lung cancer (A549) transplanted into athymic nude mice by i.v. administration of tTF-NGR (n=11) compared to the administration of tTF (n=4) or saline (0.9% NaCl, n=10). The arrows indicate the time points of injection. Data are presented as means and standard errors. The tumor growth was significantly retarded by tTF-NGR after the 4th injection in comparison to the saline group (P=.008, Mann-Whitney Test) and after the 5th tTF-NGR injection in comparison to the tTF group (P=.007, Mann-Whitney Test). Asterisks denote first day of statistical significance between tTF-NGR and saline. **B.** Effect of tTF-NGR on the growth of fibrosarcoma xenotransplants in mice. Growth inhibition of human fibrosarcoma (HT1080) transplanted into athymic nude mice by i.v. administration of tTF-NGR (n=8) compared to the administration of tTF (n=14) or saline (0.9% NaCl, n=15). Time (days) shown represent days after first treatment. Arrows indicate the time points of injection. Data are presented as means and standard errors. The tumor growth was significantly retarded at the 6th day of the tTF-NGR treatment in comparison to the saline group (P=.023, Mann-Whitney Test) and at the 6th day in comparison to the tTF group (P=.02, Mann-Whitney Test). Asterisks denote statistical significance between tTF-NGR and saline.

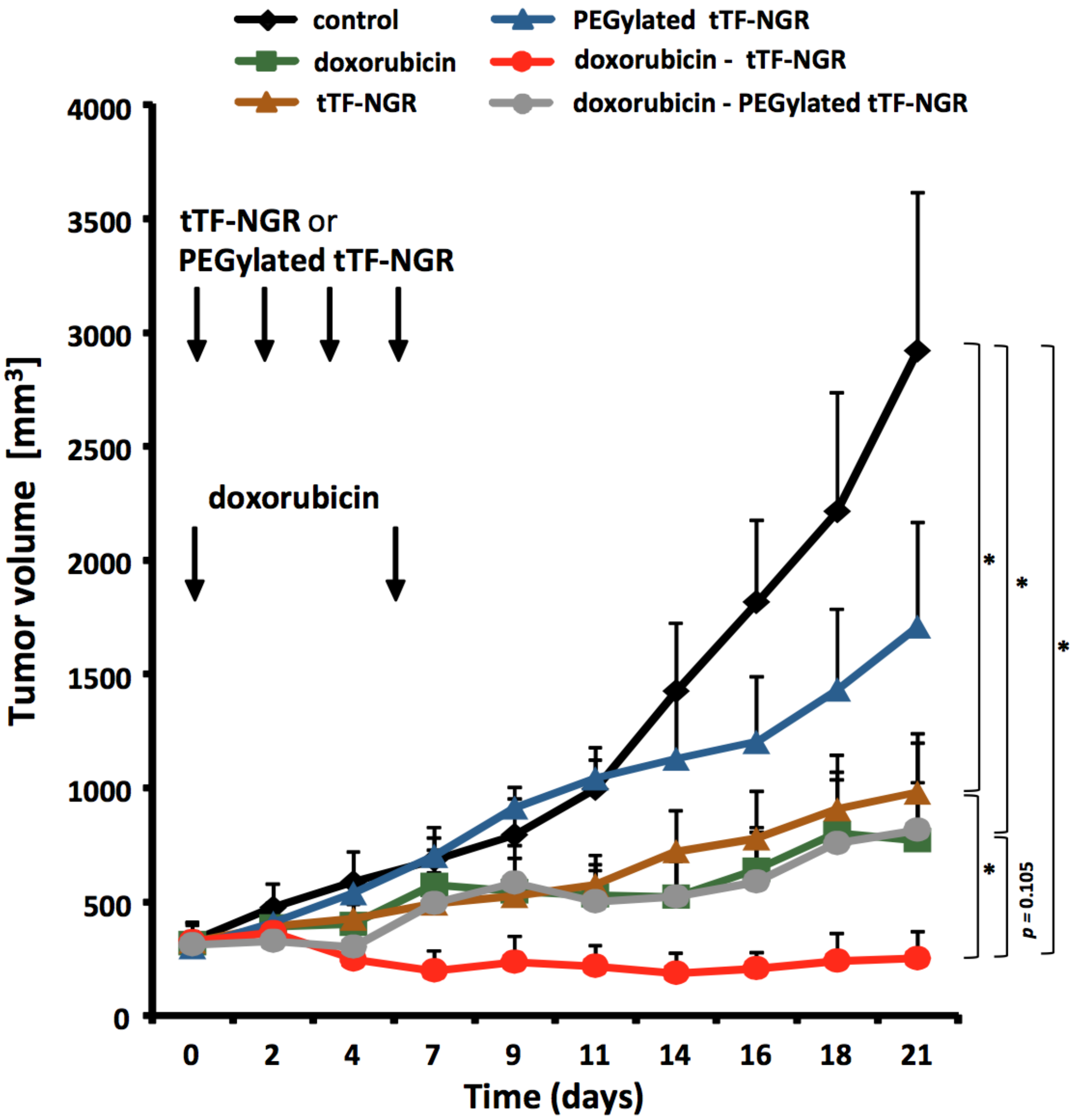

Figure 4: *In vivo* therapeutic activity against HT1080 sarcoma xenografts of combinatorial application of doxorubicin - tTF-NGR or - TMS(PEG)12 tTF-NGR. Drugs were applied intravenously at the doses and time points indicated. Data are presented as means +/- SE. Asterisks indicate statistical significance for the groups as indicated (p<0.05, *; p<0.001, **; p<0.0001; Mann-Whitney test). Therapeutic activity of doxorubicin (5 mg/kg bw, see arrow; n=5), tTF-NGR (1 mg/kg bw; n=5) or TMS(PEG)12 tTF-NGR (5 mg/kg bw; n=4), respectively (both applied every other day, see 4 arrows), or the combination of both drugs (each n=4) at identical doses using suboptimal schedules for the single drugs in a HT1080-human fibrosarcoma xenograft model. In comparison to the PBS control, the therapies with doxorubicin, doxorubicin followed by tTF-NGR, and tTF-NGR alone revealed a significant decrease in tumor volume on day 21 (p=0.0317, p=0.0286, p=0.0259, respectively); comparison of the combination schedule ‘doxorubicin followed by tTF-NGR’ versus doxorubicin alone shows a statistical trend in favour of the combination (p=0.105), while comparison of ‘doxorubicin followed by tTF-NGR’ versus tTF-NGR alone reveals statistical significance (p=0.0489).

4.1.3 Targeted delivery of tTF-NGR and functional imaging

Therapeutic activity and mode of action can be shown by different imaging techniques (Figure 5; ref. 9,13).

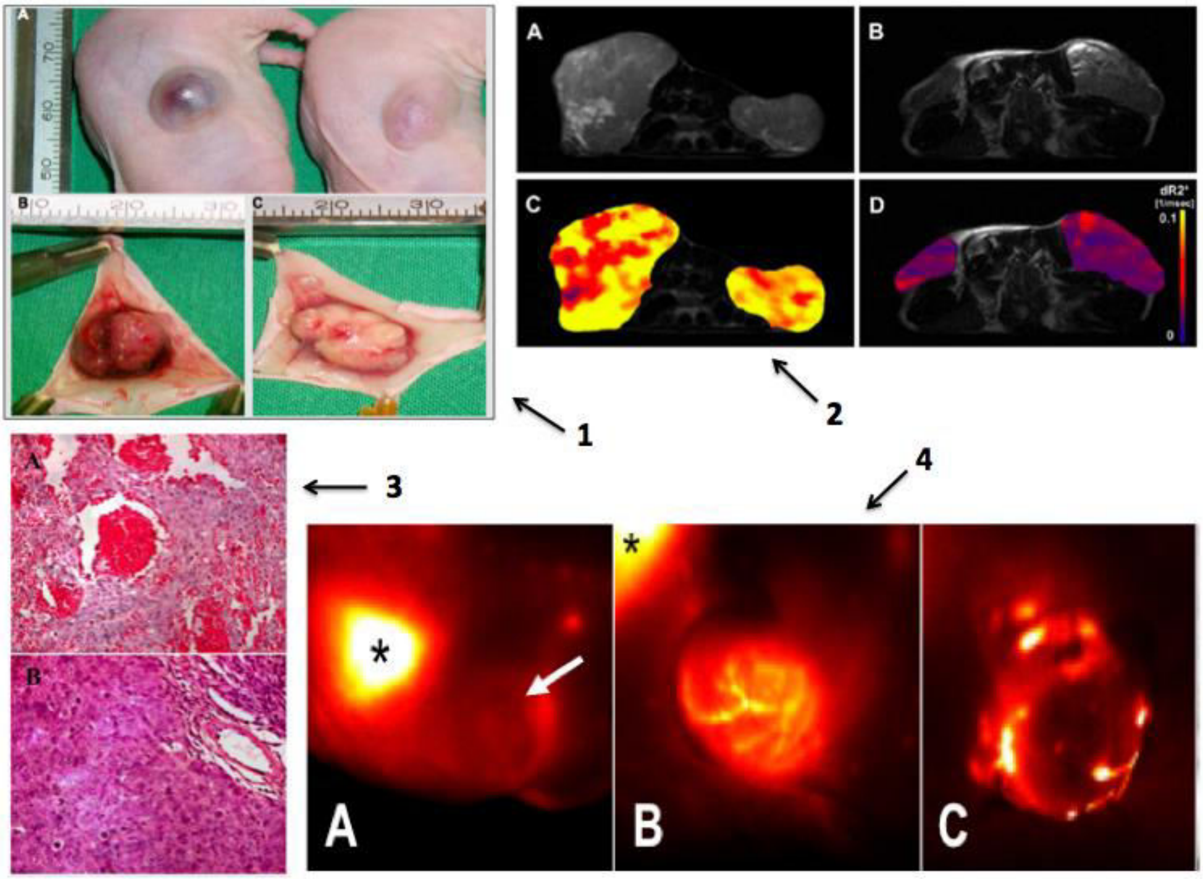

Figure 5: Imaging the mode of action of tTF-NGR. 1. Bluish coloration of the tumor (A, left: tTF-NGR. A, right: vehicle control) few hours after injection of tTF-NGR macroscopically visualizing blood pooling and vascular disruption. **2.** Intravascular contrast enhanced Magnetic Resonance Imaging (MRI) of the tumor showing anatomy of the tumors (A, B) and drastic decrease of blood flow from high blood flow (C) to low (D) few hours after tTF-NGR application. The vascular volume fraction was quantified to decrease > 1 log. **3.** H&E staining of sarcoma xenograft tissue (A, thrombosis, blood pooling and vascular disruption after tTF-NGR; B, saline control with normal vasculature). **4.** *In vivo* fluorescence reflectance imaging showing the vascular anatomy by intravascular fluorescent fibrin after tTF-NGR (B). A, before tTF-NGR; B, 1 hour after tTF-NGR; C, vascular disruption 24 hours after tTF-NGR.

4.1.3.1 tTF-NGR and Trabectedin

In addition to the observation that tTF-NGR can prolong high intratumoral drug levels of cytotoxic drugs such as doxorubicin when a sequence is given in which tTF-NGR occludes tumor vessels at the time of maximum intratumoral drug level (17), there is a second scientific basis arguing in favor of combining trabectedin with tTF-NGR: The

pro-apoptotic activity of trabectedin on human vascular endothelial cells (HUVEC) and also tumor cells significantly increases phosphatidylserine (PS) presence on the outer leaflet of the phospholipid bilayer building the cell membrane (see Fig. 6 A and B for HUVECs as an example) and by this optimized phospholipid milieu in the outer cellular membrane potentiates the pro-coagulatory efficacy of tTF-NGR within the tTF-NGR:Factor VIIa:Factor X complex on the cell membrane (Fig. 6 C). This effect is specifically dependent on the presence of PS as it can be completely abolished by masking PS via preincubation with annexin V (Fig. 6 C).

A

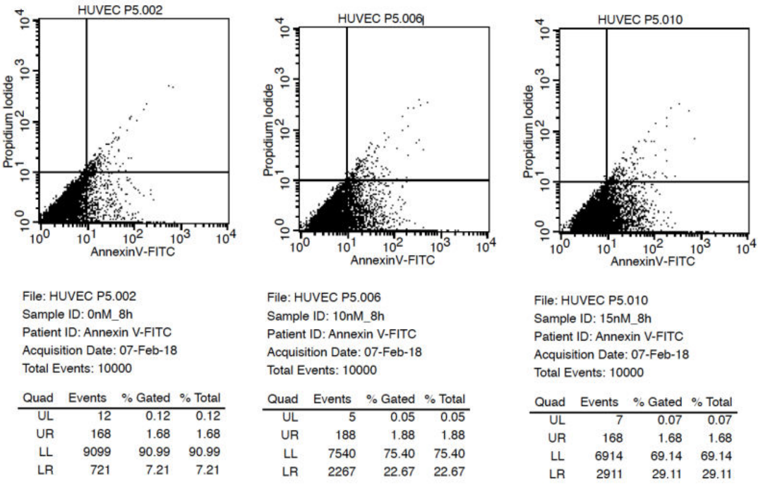

B

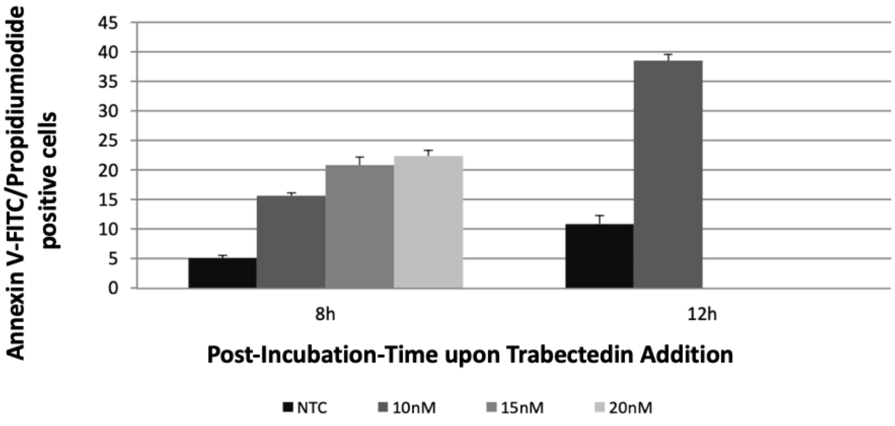

C

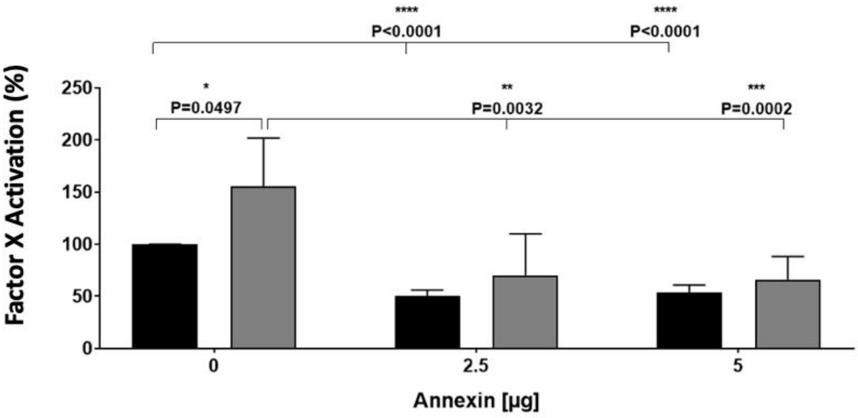

Figure 6: A) One example of a flow cytometry experiment showing PS upregulation by trabectedin on HUVEC cell membranes. PS is detected by the fluorescent PS-binding annexin V-FITC. PS-positive cells are shown in the lower right quadrant (LR) and increase from 7.21% without to 29.22% with trabectedin (15 nM, 8h incubation). **B) Evaluation of 7 different experiments** with different doses of trabectedin inducing PS upregulation on HUVECs after 8 and after 12 h by increasing doses of trabectedin (P values for all time points < 0.0001 when compared to the non-trabectedin control (NTC). Propidiumiodide was used as an internal necrosis control. **C) Trabectedin-dependent increase of procoagulatory activity of HUVECs** upon binding of tTF-NGR (black, without trabectedin, grey, with trabectedin (10 nM, 8h)) and complete abrogation of this effect by masking of PS with annexin V at different doses. Means of 3 experiments with at least 4-fold assays each + standard error and p-values.

As in tumor tissues part of the inner vascular cell layer of the neovasculature is not formed by endothelial cells, but also by tumor cells, which is termed “vascular mimicry”, we have performed analog experiments also with HT1080 human sarcoma cells instead of HUVECs (see Fig. 7).

A

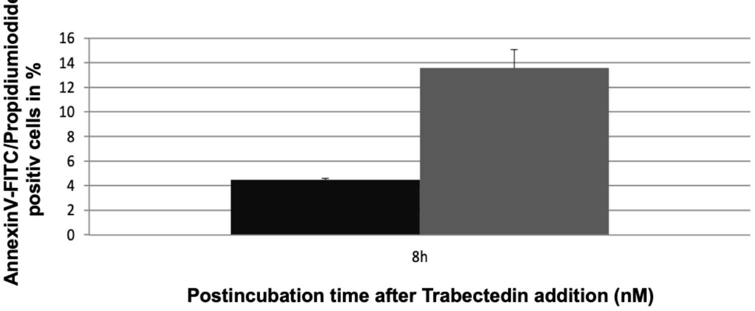

B

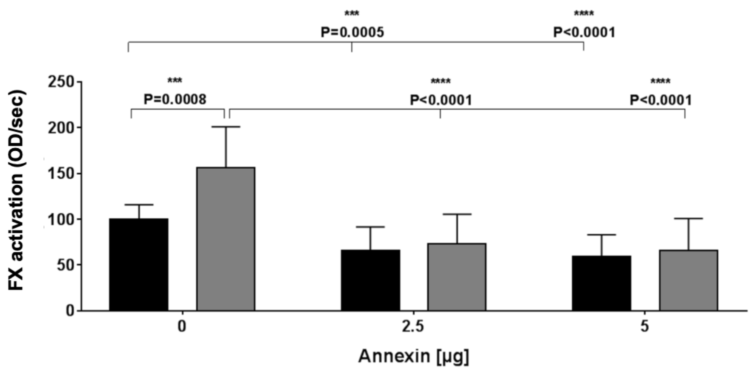

Figure 7: A) Evaluation of 6 different experiments with 10 nM of trabectedin (grey) and 8 h incubation time showing significant PS upregulation on **HT1080 sarcoma cells** when compared to the non-trabectedin control (black). Means + standard error (p value = 0.001). Propidiumiodide was used as an internal necrosis control. **B) Trabectedin-dependent increase of procoagulatory activity of HT1080 sarcoma** upon binding of tTF-NGR (black, without trabectedin, grey, with trabectedin (10 nM, 8h)) and complete abrogation of this effect by masking of PS with annexin V at different doses. Means of 4 experiments + standard error and p-values.

Thus, exploiting **synergistic mechanisms in both directions**, a *combination of trabectedin with tTF-NGR* in a pharmacokinetic approach by application of tTF-NGR approx. 5 hours after injection of trabectedin has been shown to considerably improve the therapeutic activity in comparison to the 2 single drugs in a human STS xenograft model HT1080 (Figure 8).

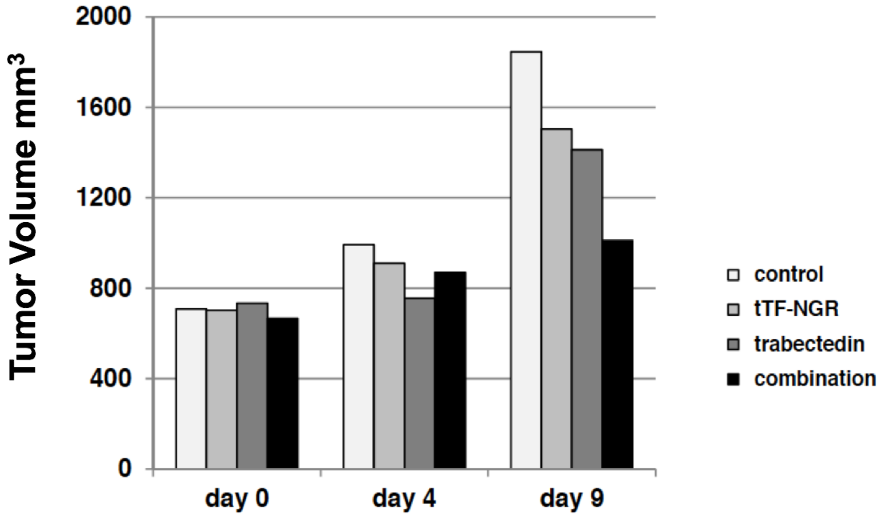

Figure 8: Combination of trabectedin with tTF-NGR. Therapeutic results in vivo in a HT1080 STS xenograft model. **Control**, PBS iv; **tTF-NGR** 1 mg/kg, day 0; **trabectedin** 0.1 mg/kg day 0; **combination**, trabectedin plus tTF-NGR (5 h after trabectedin) with identical doses and time schedule (control n=8, tTF-NGR n=9, Trabectedin n=9, combination n=11).

4.1.4 Clinical Experience with tTF-NGR

We have established a clinical-grade production process for tTF-NGR using *E. coli* and a 4-step HPLC purification (9). The production process and our GMP facility are approved by the regional government (Manufacturer´s Authorisation by Regierungs-präsidium) and the federal Paul Ehrlich-Institute (PEI). Toxicity evaluation according to EU-guidelines S6 and S9 in mouse, rat, guinea pigs, and dogs has been completed. Limiting toxicity in the most sensitive species (mouse) was pulmonary embolism, in this species tTF-NGR has a therapeutic safety window of 1:4 (therapeutic dose:LD_{10} dose=1:5). The details are presented in the IB (attached).
A list of clinical studies with tTF-NGR is presented in Table 1.

Table 1: List of clinical studies performed during the development of tTF-NGR.

Study ID	Study Design / Objectives	Population / No. of subjects	Dose Regimens
**Named patient therapy**			
	**No study design**<br>tTF-NGR via central venous access as 1-hour infusion<br>**Objective:**<br>To treat cancer patients beyond standard therapy on an individual basis	Late stage solid tumor and lymphoma patients beyond all standard treatment.<br><br>**No. of subjects:**<br>**5**	Once weekly application with intra-individual dose escalation in 0.5 mg/m^2 steps (1–4 mg/m^2, for details see summary in IB)
**EudraCT-No.:**	**2016-003042-85**		
**NCT02902237**	**tTF-NGR Phase I Study**<br>tTF-NGR via central venous access as daily 1-hour infusions for 5 days, q day 22; intra-individual dose-escalations in follow-up cycles allowed. Patients within the dose-escalation part will be treated in sequence and not in parallel.<br>**Primary Objective:**<br>To evaluate the maximum tolerated dose (MTD) and the Dose-Limiting Toxicity (DLT) of intravenously (iv) infused daily applications of tTF-NGR for 5 days every 3 weeks in patients with relapsed or refractory cancer, who had obtained all standard treatment known for their disease entity prior to entry on study.	Late stage solid tumor and lymphoma patients beyond all standard treatment.<br><br>**No. of subjects:**<br>**24**<br>24 of whom 17 were actually treated with tTF-NGR	**Starting dose:**<br>**1 mg/m^2 per day**<br><br>**DLT:**<br>Dose limiting toxicity (DLT) at **4 and 5 mg/m^2** per day<br><br>**Verification group:**<br>RPIID verification group with **3.0 mg/m^2 tTF-NGR per day (x5)**

	**Secondary Objectives:**<br>**1.** To determine the perfusion and vascular volume fraction of measurable tumor lesions vs. normal reference tissue before and after tTF-NGR application by MRI and/or CEUS as a biological surrogate parameter for biological activity of the IMP in the patients treated within the verification cohorts.<br>**2.** To obtain pharmacokinetic data.		

The named patient data are summarized in the IB (attached) and are published (8). We have a completed clinical **phase I trial** (EudraCT-No.: 2016-003042-85; NCT02902237) with tTF-NGR applied as 1-hour infusion solved in 0.9% NaCl via a central venous line in late stage cancer patients. This trial was a first-in-class trial, which was guided by repeated contrast-enhanced ultrasound (CEUS) and MRI to assess for blood flow decrease in tumor signal lesions. We used a “5-day q day 22” schedule. At 4 mg/m^2/day we have observed dose-limiting toxicity (**DLT**) as temporary troponin T has increase without any clinical sequelae. This sensitive laboratory DLT was fast reversible and allows early monitoring for subsequent dose adjustment in future trials. Further, one CTCAE grade II lower limb deep vein thrombosis (1 patient, completely resolved) was also observed at 5 mg/m^2, a central venous catheter-related venous thrombosis was observed at 4 mg/m^2 (CTCAE grade II and resolved), and a transient ischemic attack (CTCAE II resolved) in a patient with an angiosarcoma in the left atrium of the heart was observed at 3 mg/m^2, events reasonably related to, but not necessarily caused only by tTF-NGR. Thus, the trial was completed with a recommended dose for phase II (RPIID) at 3 mg/m^2/day x 5, q d 22. The investigational medical product (IMP) is given via a PORT or central venous access in 100 mL 0.9 % NaCl over a rate-controlled infusion with a 1-hour duration.

Pharmacokinetic studies of the study have indicated a terminal half life of tTF-NGR of 8-9 h. There was no accumulation with repeated cycles, as the levels before treatment were always reached before the next cycle started.

We have observed *specific inhibition of tumor blood flow* (intratumoral blood circulation) in the patients measurable with CEUS and MRI without blood flow decrease in normal organs (see IB and ref. 22). The tumor blood flow inhibition was up to >1-log-step. Further, in some metastases we have observed fast development of areas with MRI, interpreted as intratumoral bleeding and necrotic areas, similar to the observations in the xenograft models. In none of the patients treated so far a Complete Response (CR) or a Partial Response (PR) could be observed. Two patients however

had stable disease (SD) for several months after treatment. Human anti-fusion protein antibodies (HAFA) were detected in few patients without evidence of clinical symptoms (anaphylactic or anaphylactoid reactions) or neutralizing capacity when correlated with the tumor blood flow inhibition in MRI or CEUS.

In conclusion, tTF-NGR can be applied safely and observations in phase I represent proof-of-principle for tumor blood flow inhibition in the clinical situation and a promising therapeutic range. For possible side effects we have an early sensitive laboratory biomarker, and there are multiple effective methods to antagonize theoretically occurring systemic toxicity (heparin, COX-inhibitors, dual platelet inhibition with aspirin and P2Y12 inhibitors, fibrinolysis).

The detailed results of the phase I trial are presented in the IB (attached).

Clinical assessment of the *presence of CD13 in soft tissue sarcoma (STS) tissues* as the main target for tTF-NGR was done in cooperation with the Gerhard Domagk Institute for Pathology (GDI, Prof. W. Hartmann and Prof. E. Wardelmann) by performing immunohistochemistry studies on tumor slides from 97 patients with STS recently treated in our hospital (IB and ref. 20). Evaluation of CD13 expression was performed through a semiquantitative score assessing CD13 staining intensity, differentiating between expression by tumor cells and/or the vascular/perivascular compartment. Table 2 depicts the distribution and staining intensity for CD13 in these STS tissue samples.

Table 2: CD13 immunohistochemistry staining intensity and distribution according to histology (20).

Staining intensity	0	1	2	3
**Histology, n (%)**				
Undifferentiated sarcoma				
- Tumor cells	10 (20.0 %)	2 (4.0 %)	7 (14.0 %)	31 (62.0 %)
- Vascular	1 (2.0 %)	7 (14.0 %)	17 (34.0 %)	25 (50.0 %)
- Composite*	1 (2.0 %)	5 (10.0 %)	11 (22.0 %)	33 (66.0 %)
Myxofibrosarcoma				
- Tumor cells	1 (10 %)	0 (0 %)	1 (10 %)	8 (80 %)
- Vascular	1 (10 %)	1 (10 %)	1 (10 %)	7 (70 %)
- Composite*	1 (10 %)	0 (0 %)	1 (10 %)	8 (80 %)
Liposarcoma				
- Tumor cells	4 (44.4 %)	2 (22.2 %)	0 (0 %)	3 (33.3 %)
- Vascular	2 (22.2%)	0 (0 %)	3 (33.3 %)	4 (44.4 %)
- Composite*	2 (22.2 %)	0 (0 %)	2 (22.2 %)	5 (55.6 %)
Synovial sarcoma				
- Tumor cells	7 (77.8 %)	1 (11.1 %)	1 (11.1 %)	0 (0 %)
- Vascular	2 (22.2 %)	3 (33.3 %)	1 (11.1 %)	3 (33.3 %)
- Composite*	1 (11.1 %)	4 (44.4 %)	1 (11.1%)	3 (33.3 %)
Leiomyosarcoma				
- Tumor cells	2 (66.7 %)	0 (0 %)	1 (33.3 %)	0 (0 %)
- Vascular	0 (0 %)	2 (66.7 %)	1 (33.3 %)	0 (0 %)
- Composite*	0 (0 %)	1 (33.3 %)	2 (66.7 %)	0 (0 %)
Other				
- Tumor cells	5 (31.3 %)	3 (18.8 %)	2 (12.5 %)	6 (37.5 %)
- Vascular	1 (6.3 %)	4 (25.0 %)	3 (18.8 %)	8 (50.0 %)
- Composite*	1 (6.3 %)	2 (12.5 %)	5 (31.3 %)	8 (50.0 %)
**Overall frequency****				
- Tumor cells	29 (29.9 %)	8 (8.2 %)	12 (12.4 %)	48 (49.5 %)
- Vascular	7 (7.2 %)	17 (17.5 %)	26 (26.8 %)	47 (48.5 %)
- Composite*	6 (6.2 %)	12 (12.4 %)	22 (22.7 %)	57 (58.8%)
**Tumor size*****				
< 5cm	7 (31.8 %)	2 (9.1 %)	1 (4.5 %)	12 (54.5 %)
5 – 10 cm	12 (30 %)	4 (10 %)	3 (7.5 %)	21 (52.5 %)
>10cm	10 (28.6 %)	2 (5.7 %)	8 (22.9 %)	15 (42.9 %)
**Extent of resection*****				
R0	28 (29.8 %)	7 (7.4 %)	11 (11.7 %)	48 (51.1 %)
R1 or R2	1 (33.3 %)	1 (33.3 %)	1 (33.3 %)	0 (0 %)
**Disease site*****				
extremity	23 (29.9 %)	4 (5.2 %)	9 (11.7 %)	41 (53.2 %)
trunk	6 (33.3 %)	4 (22.2 %)	2 (11.1 %)	6 (33.3 %)
neck/head	0 (0 %)	0 (0 %)	1 (50 %)	1 (50 %)

*, composite indicates the highest observed intensity of either vascular/perivascular or tumor cell per case; **, overall frequency indicates n (%) of all histologies together; ***, intensity scores are according to tumor cell CD13 intensity

Positive immunostaining (scores 1 - 3) of tumor tissues with the CD13 antibody was found in endothelial cells and vessel-associated stroma cells in 92.8 % (examples are depicted in Figure 9), and in STS tumor cells in 70.1 % (examples are depicted in Figure 10). Comparison of CD13 expression pattern and expression levels revealed differences according to the histological subtypes of STS, with leiomyosarcoma and translocation-driven synovial sarcoma showing the lowest level of CD13 tumoral positivity (Table 2). Overall, a strong staining (score 3) was found in 49.5 % of the cases in tumor cells and in 48.5 % in vascular cells (Table 2).

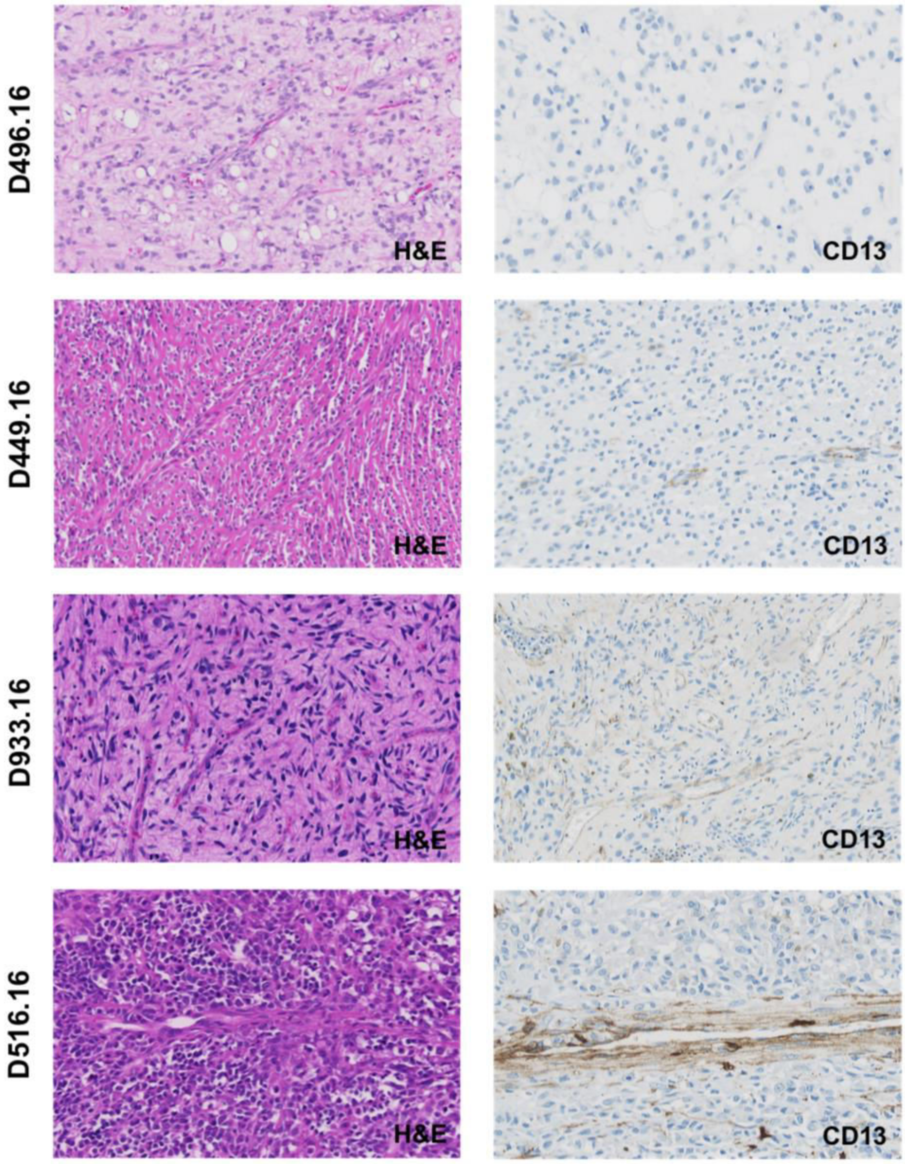

Figure 9: Different intensity degrees of vascular/perivascular expression of CD13 in soft tissue sarcomas: D496.16, intensity score 0; D449.16, score 1; D933, score 2; D516.16, score 3.

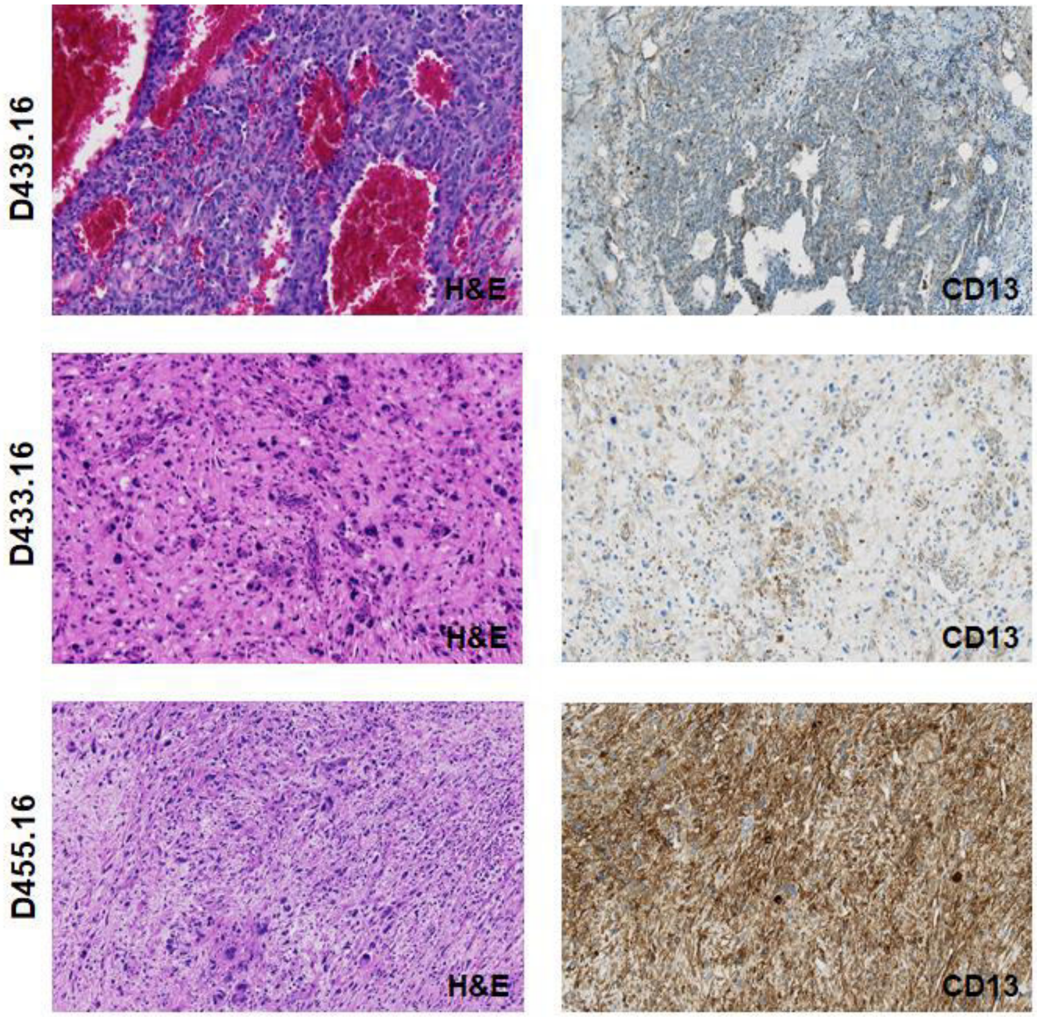

Figure 10: Different intensity degrees of tumoral expression of CD13 in soft tissue sarcomas: D439.16, intensity score 1; D433.16, score 2; D455.16, score 3.

4.1.5 Trabectedin

Trabectedin (also known as ecteinascidin 743 or ET-743) is an antitumor chemotherapy drug sold by Pharma Mar S.A. and Johnson and Johnson under the brand name Yondelis®. The European Commission and the Food and Drug Administration (FDA) have granted orphan drug status to trabectedin for soft-tissue sarcomas and ovarian cancer. The drug is a DNA minor groove binder. Favorable binding sequences are TGG, AGC, or GGC. Once bound, this reversible covalent adduct bends DNA toward the major groove, and interferes directly with activated transcription as one of multiple mechanisms. Specifically, it has been shown, that trabectedin blocks DNA binding of oncogenic transcription factors. Trabectedin also acts on compartments of the tumor microenvironment.

In 2007, the European Medicines Agency (EMA) gave authorization for the marketing of trabectedin, under the trade name Yondelis®, for the treatment of patients with

advanced soft-tissue sarcoma, after failure of anthracyclines and ifosfamide, or who are unsuited to receive these agents. The agency's evaluating committee, the Committee for Medicinal Products for Human Use (CHMP), observed that trabectedin had not been evaluated in an adequately designed and analyzed randomized trial against current best care, and that the clinical efficacy data were mainly based on patients with liposarcoma and leiomyosarcoma. However, the pivotal study did show a significant difference between two different trabectedin treatment regimens, and due to the rarity of the disease, the CHMP considered that marketing authorization could be granted under exceptional circumstances (23). Trabectedin is also approved in South Korea and Russia. In 2015, after a phase III study comparing trabectedin with dacarbaczine (24) with the US FDA approved trabectedin (Yondelis) for the treatment of liposarcoma and leiomyosarcoma that is either unresectable or has metastasized. Patients must have received prior chemotherapy with an anthracycline.

Thus, for this trial the question was to be decided whether to include patients with only liposarcomas and leiomyosarcomas, or whether to also include other entities of soft tissue sarcoma.

Upon detailed study of published literature on trabectedin activity in inducing remissions and prolonged stable disease including non-controlled cohorts and case reports and on the basis of CD13 target expression (20), we decided to also include patients suffering from other subtypes of STS beyond the L-sarcomas such as undifferentiated sarcoma (25), synovial sarcoma (25-28), and rhabdomyosarcoma (29).

4.2 Rationale for the study & benefit-risk assessment

Trabectedin as a single agent is commonly used as a standard 2nd line treatment of metastatic or refractory STS. The dose of the drug of 1.5 mg/m^2/24 h had to be reduced due to toxicity in several studies. tTF-NGR has shown preclinical activity in human STS xenografts by tumor vascular occlusion and infarction and can improve the antitumor activity of trabectedin in these models *in vivo* by entrapping trabectedin inside the tumor, which leads to a stronger antitumor activity with a longer duration. Vice versa, trabectedin increases the pro-coagulatory efficacy of tTF-NGR in the tumor vasculature. The binding side of tTF-NGR, CD13, is strongly expressed in the vascular system and/or on the tumor cells of human STS. This provides a biomarker for patient selection for this study which can be further studied for predictiveness of the activity of the combination treatment.

In a combination of tTF-NGR with trabectedin both drugs showed increased anti-sarcoma activity in comparison to standard trabectedin monotherapy in a xenograft model.

In a run-in phase of this study, 6 patients with soft tissue sarcoma will be treated with a combination of the standard protocol of trabectedin with tTF-NGR at the RPIID. Then both therapies will be randomly compared for efficacy.

The risk of this combination is small and will be further tested in the run-in safety part of the study, a potential benefit for the patients exists based on preclinical observations of synergistic effects.

5 STUDY ENDPOINTS AND OBJECTIVES

5.1 Primary endpoints and objectives

The primary objective of the trial is to evaluate whether tTF-NGR in combination with standard trabectedin chemotherapy given for unresectable or metastatic STS after failure of anthracycline-containing first line therapy or with contraindications to these drugs prolongs progression free survival, as compared with trabectedin alone.

The following **primary efficacy endpoint** will be considered (for the phase III randomized part):
- Progression-free survival (PFS) according to Response Evaluation Criteria in Solid Tumours in cancer immunotherapy trials (iRECIST, Seymour L, Lancet Oncol. 2017, ref. 30) as judged by central blinded radiology. The reason for using the iRECIST modification of RECIST for evaluation rests in the observation within preclinical studies and clinical cases, that intratumoral swelling by blood pooling and vascular disruption can occur and lead to pseudoprogressions (22, IB, and see below).
In particular, even if the assessment of PFS by a local investigator differs from the central blinded assessment, the latter assessment is considered the relevant primary efficacy endpoint.

5.2 Secondary endpoints and objectives (for the phase III randomized part of the study)

The secondary objective of the trial is to evaluate the efficacy of tTF-NGR in combination with standard trabectedin chemotherapy given for unresectable or metastatic STS after failure of anthracycline-containing first line therapy or with contraindications to these drugs with respect to the response rate and overall survival as well as to assess the safety profile of tTF-NGR combined with trabectedin.

To assess the *efficacy*, the following measurements will be considered:
- Overall response rate (ORR, consisting of CR and PR)
- Disease control rate (DCR, consisting of CR, PR, and stable disease (SD) for $\geq$ 18 weeks)
- Median progression-free survival (mPSF)
- Median overall survival (mOS)
- Overall survival (OS) rate at 12 and 18 months

To assess the *safety profile* of tTF-NGR combined with trabectedin (for both parts of the study). The
following safety endpoints will be considered:
- Adverse Events (AEs) assessment based on CTCAE v.5.0.
- Standard laboratory parameters and pharmacokinetics
- Physical examination findings including assessment of vital signs
- Analysis of patient reported outcomes (PRO)

6 OVERALL STUDY DESIGN

6.1 Overall Study Design and Plan

6.1.1 Description
Open label, randomized, controlled study in subjects with metastatic or refractory soft tissue sarcoma. CD13 positivity in central histology (grade 1+; 20) is a prerequisite for entry on study and explains part of the drop-out rate.
Approx. 150 patients will be pre-screened, at least 6 patients are treated in the safety part, 126 evaluable patients are enrolled and parallel assigned in a 1:1 fashion to one of two different arms (phase III), as outlined below. Neither trabectedin nor tTF-NGR is expected to differ in its effects on male and female patients. Therefore, in this study patient recruitment and randomization do not take account of the distribution of gender in the study population. Randomization will be stratified into composite score CD13 1+/2+ versus CD13 3+ and number of chemotherapy regimen before entry on trial: 1 versus >1. Central pathology for CD13 is a routine test, as it can predict prognosis (20).
The safety part will be conducted in 1-6 study sites (University Hospital Muenster and up to five additional study sites). The patients will be treated in-house and in sequence. A following patient can be started upon good tolerability of one cycle in the previous patient. The phase III randomized part will be multicentric with 9-14 study sites, whereby each study site is a high volume sarcoma center and active in the German Interdisciplinary Sarcoma Group (GISG). Permuted block randomization will be done using a suitable software (e.g. randomizer.at).

The study is divided into 2 parts to be performed in sequence:

Safety run-in part:
Before the randomized phase III part of the study, there will be a *safety cohort* of a minimum of 6 patients obtaining at least 2 cycles each of the combination outlined in arm 2 (see below, 1.5 mg/m^2 trabectedin plus 3 mg/m^2 as a starting dose of tTF-NGR) to confirm safety of this combination. In case of dose-limiting toxicity (DLT) in this safety cohort, a dose-modification protocol for tTF-NGR to 2 mg/m^2 is planned, and in case of further tolerability problems further deescalations in 0.5 mg/m^2 steps of tTF-NGR

and/or further by a reduction of application days are planned. The safe dose and the final number of application days is then transferred to the randomized part of the study. The final dose of tTF-NGR established as safe in this combination has to be applied to 6 patients with 2 cycles each. The randomized (phase III) part of the study will be opened after judgement of the safety in the safety cohort by the DSMB, the Paul-Ehrlich Institute (PEI) and the Ethics Committee.
The full pharmacokinetics (full PK) characterization will be performed in safety cohort including 6 patients.

If full PK profiles were not determined for 6 patients in the safety part, the full PK characterization is to be continued with additional patients in Arm 2 of the Phase III randomized part until full PK characterization has been completed for 6 patients.

The safety part of the study will be performed in few centers only (start with UKM and up to five additional study sites). The patients will be treated in-house and in sequence (a 2nd patient can start treatment as soon as the previous has tolerated one cycle of the combination without DLT). DLT is definded as in the phase I study with tTF-NGR (ref. 31) according to CTCAE 5.0: *Dose Limiting Toxicity (DLT):* DLT is defined as occurrence of toxicity of Grade 3 or more according to CTCAE version 5.0 for non-hematological toxicity or Grade 4 or more for hematological toxicity. DLT must be considered by the investigator as being causally related to the IMP. In case the investigator comes to no decision, the data safety monitoring board is to be involved. The patients are monitored daily and study specific examinations are performed as outlined in Table 4 (Arm 2 of the randomized part).

In case 6 patients have obtained 2 cycles of a combination with a fix dose of tTF-NGR (12 cycles) without DLT, a DSMB meeting is convened to discuss the results and advice on a tTF-NGR dose for the randomized part of the study.

The study protocol has to be amended accordingly before starting the randomized part of the trial.

Randomized part:

- ARM 1: Patients will receive 1.5 mg/m^2 trabectedin as a 24-hour central intravenous (iv) infusion on day 1, q d 22 x until disease progression or contraindications of further application (premedication according to institutional guidelines: e.g. 20 mg dexamethasone)

- ARM 2: Patients will receive standard trabectedin according to arm 1 plus the safe dose for the safe number of days (</= 4 days) according to the safety run-in experience of tTF-NGR (1-hour rate-controlled infusion, PORT or central venous access, 0.9 %NaCl ad 100 mL) per day following each trabectedin cycle (1 hour interval between the end of trabectedin infusion and tTF-NGR: e.g.: trabectedin

on monday 8 am to tuesday 8 am followed by tTF-NGR on tuesday 9 am and on the following days, q d 22 x until disease progression or contraindications against further application

As the evaluation of the study results is intention-to-treat based, all patients after randomization will be part of the efficacy population as evaluated by central iRECIST evaluation after end of study.

Therapy in both arms can be given on an out-patient basis. Some patients may require a hospitalization for the 24 hours of trabectedin infusion. All patients receive best supportive care (BSC) according to institutional guidelines.

Anti-cancer activity will be assessed clinically at week 9 and clinically and by imaging at week 18, and then every 9 weeks (adjusted to cycle length of 3 weeks) until iCPD. Decision on application of next cycle at week 9 will be clinical. Imaging and clinical-based decision will follow. This procedure is described in detail below.

Safety assessment will be performed on an ongoing basis during study participation, including standard laboratory assessments. The incidence of AEs will be summarized by severity in all patients with at least one study drug intake.

Patients will be treated in repeated cycles until one of the following criteria are met (in the absence of any other withdrawal criteria):

1) unacceptable toxicity precluding further therapy,
2) disease progression (as described below),
3) the patient or the investigator requests treatment discontinuation.

Patients who stop study treatment in the absence of progressive disease should not receive further line cancer therapies before their disease progresses, unless there are clear reasons to continue with alternative therapies.

The application of the next therapy cycle can be postponed for up to 21 days due to the following reasons:

1) due to patient's clinical conditions or
2) by the decision of the investigator after discussion with the Coordinating Investigator Prof. Schliemann

Detailed tumor assessments after start of therapy are performed at week 9 (+/- 1 week), followed by every 9 weeks (+/- 1 week, independent from cycle length of 3 weeks) until iCPD. Whenever iUPD is stated it has to be confirmed as iCPD by imaging this individual patient 9 weeks later. In case clinically relevant progression is seen, the investigator can decide to perform an earlier tumor

assessment (see unscheduled visits, page 53). In case EOT is not determined by iCPD, tumor assessments will go on after EOT until iCPD.

Post-study treatment (treatment with another anti-cancer agent) is according to investigator´s choice, but is recorded in the eCRFs with name of drug/procedure and duration. At iCPD follow-up will end but time of death will be recorded for estimation of OS. Patients not withdrawn from the study will be followed until iCPD within the study or until death. Survival status will be collected also for patients withdrawn from the study until death. After iCPD, the patients will be seen in 3-monthly intervals outside the study to record further OS.

Patients will go off treatment in case of

- clinically dangerous PD at week 9 (as judged by the local investigator after communication with the Coordinating Investigator/study center),
- or in case of iRECIST-confirmed disease progression (iCPD, as judged by the study center of the patient) beginning at week 18 after treatment initiation),
- or if the study drug is discontinued for any other reason, whatever occurs first (end of study treatment).

Description of clinical and imaging judgement during therapy (= tumor assessements)

The reason for using an iRECIST modification of RECIST for evaluation rests in the observation within preclinical studies and clinical cases treated with tTF-NGR (phase I), that intratumoral swelling by necrosis, blood pooling, vascular disruption and hemorrhage can occur and lead to pseudoprogressions (for preclinical experience and phase I case reports see IB). Thus, although mechanisms leading to pseudoprogression are different, this study uses a modification of iRECIST for determination of progression and PFS in analogy to studies with checkpoint inhibitors (30). iRECIST judgement and comparison of PFS between both arms will be made in a blinded fashion by an independent imaging core lab [redacted] Ad hoc iRECIST judgement for determination of a safe end of study treatment (EOT) for individual patients will be done by the local investigators in the local study centers.

For **determination of EOT for an individual patient** the following procedure is used (TP=timepoint, iRECIST on the basis of RECIST v.1.1 is used):

TP0: clinical and imaging examination before start of treatment. Assessment of target lesions as defined in the eCRF. **For all time points: Transfer of pseudonymized imaging pictures (CD, DVD) and imaging results of a patient to the study center has to be performed after each imaging time point.**

TP1 (in week 9 (+/- 1 week), after 3 planned cycles of treatment, but independent from actual cycle times): Restaging with imaging and clinical judgement is done with

identical procedure as on TP0. EOT decision by local clinical investigator according to clinically critical progression or according to imaging results with critical progression and "danger signs". Local investigator communicates unconfirmed progression by iRECIST (iUPD) (30) or PD results with Coordinating Investigator/study center before decision. In case of clinically non-critical PD (possible iUPD), SD, PR or CR therapy is continued for 3 further cycles.

TP2 (in week 18 (+/- 1 week), after 6 planned cycles of treatment, but independent from actual cycle times): Restaging with imaging and clinical judgement is done with identical procedure as on TP0. EOT decision by local clinical investigator according to imaging (question: iUPC conversion to confirmed progression by iRECIST (iCPD)). For determination of iCPD or PD previous imaging results at nadir of target lesion measurements are compared with the results at TP2 in the local center. Communication of results with Coordinating Investigator/study center before decision is offered. Subsequent therapy after EOT is according to local investigator, but is recorded in eCRF with name of drug/procedure and duration. In case of SD, PR or CR therapy is continued for 3 further cycles.

TP3 and following **TPx** (every 9 weeks (+/- 1 week): Restaging with imaging and clinical judgement is done with identical procedure as on TP0. EOT decision by local clinical investigator according to imaging (iCPD). In case of iCPD upon comparison of TP3 (or TPx) with nadir measurement of target lesions, decisions are according to TP2. In case of SD, PR or CR therapy is continued for 3 further cycles.

Detailed **scenarios** are given in ref. 30. It is not anticipated that iUPD with the mode of action of tTF-NGR will continue longer than 18 weeks after start of therapy. Final comparisons of therapy arms are done by central radiology [redacted]

Obligatory imaging methods: Contrast-enhanced MRI. For lung imaging contrast-enhanced CT.

Data organization and mailing: All scans must be stored on one DVD/CD per patient after pseudonymization and marked with center number, patient identification number (patient ID), and organized in folders according to TP of imaging. CDs/DVDs with a complete data set must be sent to the study center in Muenster after every assessment of a local investigator.

The **determination of progression-free survival (PFS) according to iRECIST** is done by central radiology [redacted] in a blinded manner.

6.1.2 Planned Duration of Study

For several reasons including the COVID-19 pandemic the time line of the study had to be updated:

- Planned start date of the study (FPFV, first patient first visit): Q4, 2021
- Planned end date of recruitment (LPFV, last patient first visit): Q4, 2027
- Planned end date of the study: corresponds to last patient last visit (LPLV)

• Planned duration of recruitment for the randomized part: 60 months

6.1.3 Premature Discontinuation of the Study

The Coordinating Investigator (Prof. Schliemann following consultation with the sponsor) has the right to discontinue this study at any time and for any reason. If the clinical study is prematurely terminated, the investigator has to promptly inform the study patients and should assure appropriate therapy and follow-up for the patients. If the study is prematurely discontinued a detailed written explanation has to be provided to the EC and to the competent authorities and all procedures and requirements pertaining to the archiving of the documents have to be observed.

Possible criteria for the Coordinating Investigator for inducing study termination or a temporary halt of the study include observation of DLT in the patients treated in the randomized phase of the study jeopardizing the safety of the patients treated or planned to be treated, any medical reason leading the coordinating investigator to change the risk benefit assessment of the study with the result of elevated risk for patient´s safety, any situation not allowing further study conduct as planned induced by e.g. aggravating pandemic situations, any DSMB advice to terminate the study on the basis of safety issues or lack of efficacy (futility).

In addition, the sponsor has the right to terminate the trial prematurely for safety reasons or for administrative reasons not allowing further conduct of the trial.

6.1.4 Stopping/Pausing Rules

Troponin T hs increase was the event defining DLT in the phase I study (see IB). It indicates hypoxic cell damage in different tissues, but mainly in the myocardium. By defining DLT with increase of Troponin T hs to values above 50 ng/L, it was possible to avoid clinical DLT in the patients treated with tTF-NGR. Thus, Troponin T hs or Troponin I hs will be used as a safety biomarker to monitor and modify tTF-NGR application in this trial. However, since Trabectedin can also induce Troponin T hs or Troponin I hs value increase and in view of the kinetics of appearance of Troponin T hs or Troponin I hs increase following tTF-NGR and the terminal half lifes of both drugs, the Coordinating Investigator can define DLT and treatment modifications on an individual basis. There are cases with Troponin T hs or Troponin I hs elevation due to inflammatory reactions. Whenever this is suspected, an attempt to reduce the values with anti-inflammatory drugs such as Ibuprofen should be discussed with the Coordinating Investigator. Although corticosteroids have been observed to decrease troponin T hs values, they should not be used for this purpose (for reasons see IB).

Thus, it is important for all investigators to communicate laboratory values under study treatment with the Coordinating Investigator before starting a next cycle.

The following basic rules have to be observed:

1. Troponin T hs levels above 50 ng/L directly before entry on study are an exclusion criterium for study participation.
2. Troponin T hs has to be measured before the application of tTF-NGR and daily during the treatment until day 5 of a cycle.
3. Each increase of Troponin T hs from values below 14 ng/L into the “grey zone” between 14 – 50 ng/L or to above 50 ng/L, or from values between 14 – 50 ng/L into the pathological range >50 ng/L have to be immediately discussed with the Coordinating Investigator (T-con) for judgement and appropriate counter measures (see point 4).
4. Troponin I hs can be used instead of troponin T hs with other ranges for normal, “grey zone” and pathological values according to the assay used.
5. Dose modifications of tTF-NGR such as
   - end of study treatment for this patient,
   - stop of tTF-NGR therapy for this cycle and later re-institution,
   - postponement of the next cycle
   - the re-institution dose level for tTF-NGR (e.g. - 0.5 mg/m^2), or
   - anticoagulation

   are decided by the Coordinating Investigator and the local Investigator during a T-con.

In case of AEs other than Troponin T hs (or I) hs elevation, that in the judgement of the Coordinating Investigator do not recommend continuation of therapy or a next cycle at the same dose level of tTF-NGR, the rules under 6.1.4. point 4 apply equally. The doses and schedules of the treatment in both arms of the study should be executed as exact as possible. All deviations in time schedule and application as dictated by the clinical practice and necessity e.g. from the patient or the physicians team side are to be discussed and agreed upon with the Coordinating Investigator *and are to be clearly documented.*

6.2 Discussion of Overall Study Design

In the phase III randomized part of this study 126 patients will be randomized 1:1 to two different groups. Primary objective is assessment of efficacy of tTF-NGR in combination with trabectedin measured as progression-free survival in combination treatment group (Arm 2) versus trabectedin monotherapy (Arm 1).

6.3 Selection of Study Population

After being informed about the study by the investigator and after having signed the patient informed consent form, patients will be screened for eligibility. Screening procedures should be performed within 14 days prior to treatment start. Standard procedures, which are part of routine patient care, are not considered as study-specific procedures, but may be used and collected to determine eligibility.

6.3.1 Inclusion Criteria

1. Patients of all genders (female, male, diverse), with no restriction regarding ethnic or religious background age 18 – 75 years.
2. Patients with advanced or metastatic soft-tissue sarcoma after failure of anthracycline-containing first line therapy (or anthracycline-containing adjuvant therapy within 12 months before entry on study) or with contraindications to these drugs
3. Patients must have histological evidence of high-grade advanced unresectable or metastatic soft tissue sarcoma (grade 2 – 3) according to the FNCLCC grading system. The following tumor types are included:
    - Dedifferentiated liposarcoma
    - Myxoid liposarcoma (high grade)
    - Pleomorphic liposarcoma
    - Adult fibrosarcoma
    - Myxofibrosarcoma (high-grade)
    - Leiomyosarcoma
    - Rhabdomyosarcoma (alveolar, pleomorphic)
    - Angiosarcoma
    - Synovial sarcoma
    - Undifferentiated sarcoma

Tumor types not listed above may be included upon communication with Coordinating Investigator.

The following tumor types will not be included:

- Gastrointestinal stromal tumors (GIST)
- Epitheloid sarcoma
- Alveolar soft part sarcoma
- Desmoplastic small round cell tumor
- Chondrosarcoma
- Osteosarcoma
- Ewing sarcoma (including CIC-rearranged sarcoma and Sarcoma with BCOR alterations)

4. CD13 positivity with a score of ≥ 1 (20) by central pathology (GDI Münster)
5. Patients must have at least one unidimensionally measurable lesion by adequate imaging as defined by RECIST criteria 1.1. This lesion should not have been irradiated during previous treatments
6. Life expectancy of at least 3 months
7. Eastern Cooperative Oncology Group (ECOG) Performance Status ≤ 2
8. No contraindications for trabectedin (see attachment)
9. Negative serum pregnancy test for females of childbearing potential* within 14 days of starting treatment

10. Informed consent signed and dated to participate in the study
11. Willingness and ability to comply with the scheduled visits, treatment plan, laboratory tests and other study procedures

* Women of childbearing potential (WOCBP) must be using, from the screening to 3 months following the last trabectedin (Arm 1) or the last last study drug (Arm 2) administration, highly effective contraception methods, as defined by the "Recommendations for contraception and pregnancy testing in clinical trials" issued by the Head of Medicine Agencies' Clinical Trial Facilitation Group (www.hma.eu/ctfg.html) and which include, for instance, progesteron-only or combined (estrogen- and progesteron-containing) hormonal contraception associated with inhibition of ovulation, intrauterine devices, intrauterine hormone-releasing systems, bilateral tubal occlusion, vasectomized partner or sexual abstinence. Pregnancy test will be repeated monthly. For men contraception methods should be performed for 5 months after the last application of trabectedin (Arm1) or study drug (Arm 2).
Women of childbearing potential are defined as females who have experienced menarche, are not postmenopausal (12 months with no menses without an alternative medical cause) and are not permanently sterilized (e.g., tubal occlusion, hysterectomy, bilateral oophorectomy or bilateral salpingectomy)

6.3.2 Exclusion Criteria

1. curative therapy available
2. clinically significant unrelated illness, which in the judgement of the investigators could compromise the patient's ability to tolerate the IMP or be likely to interfere with the study procedures or results
3. immobilized tumor patients (wheel chair etc.) with increased risk for DVT
4. known hypersensitivity reactions to prior application of *E. coli*-derived material
5. history of coronary heart disease, stroke, transitent ischemic attacks, pulmonary embolism, or deep vein thrombosis. **For reason of mechanism of action of tTF-NGR, exclusion of patients with a history of any of the vascular conditions mentioned is important. Clinical suspicion of coronary heart disease must be further checked e.g. by cardiac MRI or myocardial scintigraphy to exclude coronary heart disease.**
6. known hereditary syndromes with elevated thromboembolic risk (FV Leiden and prothrombin mutations (G20210A), hereditary antithrombin, protein C and S deficiency, and antiphospholipid syndrome) after one or more clinical thromboembolic events
7. patients with hereditary vascular disorders (such as Klippel-Trenauny-Weber syndrome) with increased thromboembolic risk.
8. patients with a Khorana score of (Khorana AA, et al. J.Clin. Oncol. 2009, 27, 4839–4847, attached to this protocol) of $\geq$ 3
9. elevated Troponin T hs (> 50 ng/L) or elevated Troponin I hs before entry on study
10. presence of active central nervous system (CNS) disease and/or CNS vascular abnormalities detected by MRI or CT

11. no adequate bone marrow function, absolute neutrophil count (ANC) < 1.0 x 10^9/L, platelets < 50 x 10^9/L (for trabectedin actually < 100 x 10^9/L – to be decided by the investigator on an individual patient basis) and hemoglobin (Hb) < 8.0 g/dl.
12. chronically impaired renal function or creatinine ≥ 2.0 x upper limit of normal (ULN).
13. inadequate liver function (alanine aminotranserase (ALT), aspartate aminotranserase (AST), alkaline phosphatase (ALP) or total bilirubin ≥ 2.5 x ULN) unless due to liver metastasis (decision by the investigator)
14. fibrinogen < 150 mg/dL, and/or International Normalized Ratio (INR) > 1,5 (global coagulation parameters can be discussed with the Coordinating Investigator prior to entry on study)
15. female patients with child-bearing who do not agree to exclusion of potential pregnancy by adequate testing within 48 hours prior to entry on study
16. females of childbearing potential as well as fertile males who do not agree to use a highly effective form of contraception (Pearl Index < 1) during the study and for 3 months (females) following the last trabectedin (Arm 1) or last study drug (Arm 2) administration and 5 months (males) following the last dose of trabectedin (Arm 1) or study drug (Arm 2)
17. women with breast-feeding activity
18. concomitant use of any other investigational agent (agent for which there is currently no approved indication from regulatory authorities) or any other anti-cancer drug
19. concomitant enrolment in another clinical trial interfering with the endpoints of this study.
20. any medical condition which could compromise participation in the study according to the investigator's assessment
21. prophylactic or therapeutic anticoagulation within the last 3 days
22. presence of active and uncontrolled infections or other severe concurrent disease, which, in the opinion of the investigator, would place the patient at undue risk or interfere with the study
23. concurrent malignancies other than STS, unless the patient has been disease-free for at least 2 years
24. serious, non-healing wound, ulcer or bone fracture; not completed wound healing from previous wounds and/or surgery
25. no central venous port system in place (the option of other central venous access than a port should be discussed with the Coordinating Investigator).

NOTE: Outliers of laboratory values can be disregarded and set aside as exclusion criteria by a Coordinating Investigator´s decision. The conditions for the use of trabectedin as specified in the Summary of Product Characteristics are to be followed according to institutional guidelines for standard of care.

6.3.3 Subject Withdrawal from further treatment (Early Termination)
The patient, or where applicable, the patient's legally acceptable representative(s) can withdraw consent for participation in the study at any time without prejudice.

Patients may be excluded from further study treatment at any time at the discretion of the investigator or sponsor for safety or, behavioral reasons, or the inability of the subject to comply with the protocol-required schedule of study visits or procedures at a given study site.

In addition, patients *must* be excluded from further study treatment if any of the following occur:

- Overall response of 'progressive disease' (PD) at evaluation times according to iRECIST criteria (as described above), or clinical diagnosis of critical progressive disease (e.g., symptomatic deterioration without proof of progressive disease, as described above).
- Receipt of any investigational agent other than study drug.
- Pregnancy or refusal to use appropriate contraception.
- Loss of follow-up.

Patients who prematurely discontinue the study will be asked to return to the study site for end of study treatment (EOT) evaluations. Attempts to ascertain the patient's data are made even beyond the 18 months period.

6.3.4 Patient replacement criteria
The phase III randomized study results will be evaluated as intention-to-treat. Thus, any patient enrolled and randomized will stay in the evaluation cohort and will not be replaced.

6.3.5 Patient screening, enrollment and identification
After signing the informed consent form, each patient will be assigned by the site staff a temporary progressive code for unambiguous patient identification number (tUPN) during the screening procedures. After enrollment, each patient will receive by the Sponsor a unique patient identification number (Patient ID) for unambiguous identification during the study. A patient can be rescreened (if the Sponsor agrees) only once and only if the conditions, which caused the first screening failure, were transient (e.g. lab parameters which could be easily corrected, for example rehydration in creatinine increase). The patient will receive a new tUPN.

6.4 IMP or Study Drug

6.4.1 IMP Composition
A complete record of the shipment of all study medication will be maintained in the TMF. Other detailed information such as handling instructions, storage, recall

procedure, etc. will be included by the sponsor in the **Pharmacy Manual** delivered to the study center.

6.4.1.1 tTF-NGR

The study drug is tTF-NGR, tTF-NGR will be supplied by the UKM GMP facility (head of production laboratory: Dr. rer. nat. C. Schwöppe, QP, address see Laboratory Manual) in pyrogen-free 10 ml glass containers as frozen (≤ -65°C), liquid, sterile, apyrogenous solution:

The product is to be further diluted by 0.9% NaCl at the bedside to a final volume of 100 mL and immediately being administered intravenously (i.v.; central PORT system) by rate-controlled 1-hour infusion. Filter requirements for tTF-NGR infusions are described in the Pharmacy Manual. An infusion of 250 mL 0.9% NaCl after each tTF-NGR application is recommended to clean the PORT. Further, the patient should be motivated to walk after the tTF-NGR infusion for 30 - 60 minutes, whenever possible.

6.4.2 IMP Labeling, Storage and Packaging – tTF-NGR

Other detailed information such as stability data, packaging, delivery shipping temperature, storage, recall procedures, etc. will be included and supplied by the Sponsor in the ISF for each center. All study drug supplies must be stored at the temperature indicated by the sponsor. Study drug that has been stored differently from the sponsor's recommendations **must not** be used unless the sponsor provides written authorization for use. In the event that the use cannot be authorized, study drug supply must be replaced with fresh stock supplied by the sponsor.

The sponsor will supply the investigational study drug tTF-NGR. The investigator (or pharmacist) will make an inventory and acknowledge receipt of all shipments of study medication.

6.4.3 Identity of IMP

6.4.3.1 tTF-NGR

- Dosage and Administration of tTF-NGR

The appropriate dose of tTF-NGR will be diluted up to a total infusion volume of 100 ml of 0.9% sterile sodium chloride (NaCl) solution. Using an infusion pump, the tTF-NGR containing infusion will be automatically delivered over 60 minutes (1 hour) via central venous (port) access.

- Dose and schedule of tTF-NGR

tTF-NGR will be administered as defined by the results of the safety run-in cohort (e.g. on days 2 and 3) of each 22-days cycle. The application of tTF-NGR on day 2 of each cycle is to be started within 1 hour after the end of the trabectedin infusion. The tTF-NGR dose is to be adjusted based upon patient's body surface area. Individually calculated dose may be rounded up or down by a maximum of 5% to achieve easily

manageable volumes. The single dose per day planned is determined by the safety part of the study.

- Dose modifications of tTF-NGR

For dose modifications see chapter 6.1.4 Stopping/Pausing Rules. Each dose modification must be discussed and agreed upon with the Coordinating Investigator.

6.4.3.3 Premedication

Premediction for trabectedin is according to standards of the center (premed: e.g. 20 mg dexamethasone).

6.4.4 Therapies other than study drug

6.4.4.1 Standard of care chemotherapy: Trabectedin

Trabectedin is considered a non-investigational medicinal product (NIMP) and will be given “in-label”. It is not provided by the Sponsor and is either prescribed or obtained from the pharmacy of each center as usually done outside a trial. For details, please refer to the SmPC of trabectedin as attached to this protocol.

- <u>Dosage and Administration of trabectedin</u>

  Trabectedin will be given “in-label”, and it is either prescribed or obtained from the pharmacy of each center as usually done outside a trial. Please refer to the drug information (“Fachinformation”/SmPC) attached to this protocol.

- <u>Dose and schedule of trabectedin</u>

  Trabectedin 1.5 mg/m^2 will be given as a 24-hour central intravenous (IV) infusion on day 1 (e.g. Monday to Tuesday), qd 22 until disease progression or contraindications against further application (premed: 20 mg dexamethasone).
- <u>Dose Modification of trabectedin</u>

  Dose modification of trabectedin should be performed considering the SmPC according to local standards of the study center and must be documented in the eCRF.

6.4.5 Other concomitant therapies

- *Permitted concomitant therapy*

– All medications needed for pre-existing diseases or for diseases or conditions arising during the study.
– Prophylactic anti-emetics and anti-emetic treatment according to local guidelines or standards if indicated in the opinion of the investigator.

- Corticosteroids have to be given as requested by trabectedin-Fachinformation and according to institutional guidelines before application of trabectedin
- Hematopoietic growth factor G-CSF may be used as per approved package inserts, if deemed appropriate by the investigator.
- Approved agents including those for best supportive care according to local guidelines and/or standards for evaluation and treatment.
- Corticosteroids to treat CTCAE grade ≥3 hypersensitivity reactions.
- Palliative radiation of tumor deposits is allowed according to standards of the study center. Radiation therapy should be documented with site, dose and time. Irradiated lesions must not be taken as indicator lesions for iRECIST judging of therapy efficacy.
- As trabectedin is CYP3A4-dependent, strong CYP3A4 inductors or inhibitors should be avoided.

- *Non-permitted concomitant therapy*
- Treatment with any investigational agent other than study agents.
- Anti-cancer cytotoxic or non-cytotoxic therapy other than that specified in the protocol.
- GM-CSF and erythropoietin should NOT be given.

6.4.6 IMP Compliance
The investigator will be responsible for adequate and accurate accounting of study drug usage. The investigator or designee will administer the study drugs only to individuals included in this study, following the procedures set out in this study protocol. The products will be prepared according to documents included in the ISF. The date, dosage, and time of the administration will be recorded on the drug accountability form. The investigator will track study drugs received, used and wasted and will retain all unused or expired products until the sponsor is satisfied that the study drug accountability records are correct. Thereafter, all unused study drug is to be returned to the sponsor or destroyed at the investigational site. An overall summary of study drug supplied, received, wasted, used and returned will be prepared at the conclusion of the study.

6.4.7. Risk mitigation, safety monitoring and interventional measures for side effects according to Reference Safety Information (RSI*)*

For the safety measures concerning increase of troponin T hs or troponin I hs, the investigator and the study team are referred to chapter 6.1.4. of the study protocol. In addition to local guidelines for supportive care, the following recommendations are given with respect to the risks for AEs connected with tTF-NGR as outlined in the RSI (IB, chapter 6.10.):

For **general risk mitigation of thromboembolic adverse events** the inclusion and exclusion criteria for the study have to be observed.

For **risk mitigation of thromboembolic adverse events**, the patients included in this study must not be immobilized by their disease or other causes (see chapter 6.3.2. of the protocol).

At the first visit the investigators are requested to particularly **explain** to all patients in Arm 2 of the study the **typical symptoms of thromboembolic events** and ask them to be particularly alert in this respect and to immediately contact the study center/team in case any symptoms for thromboembolic events occur. This should be done together with the **hand out and explanation of the "Patientenkarten mit Infografiken"**.

For **further risk mitigation of thromboembolic adverse events**, the patients are requested to walk for 30+ min after the infusion and regularly keep mobile and often exercise thereafter (see "Patientenkarten mit Infografiken" distributed and explained).

At each visit (see Table 4) the investigators are requested to particularly but not exclusively look for clinical or laboratory signs of thromboembolic events and ask the patients again to immediately contact the study center/team in case any symptoms for thromboembolic events occur.

In case of a suspicion of a thromboembolic event the appropriate diagnostic measures are to be taken immediately according to the study center guidelines (e.g. imaging) and **upon diagnosis** obligatory interventions are

1. to withheld further tTF-NGR application,
2. to start therapy including anticoagulation according to study center guidelines (e.g. LMW heparinization with enoxaparin in-house),

to contact the Coordinating Investigator or Deputy Coordinating Investigator (see page 2 of this protocol) for further procedures, and to perform SAE reporting.

One patient in the 1mg/m^2 x 4 days tTF-NGR cohort developed a small pulmonary arterial embolus in the 2nd cycle. Thus, the tumor **reassessment examinations with a CT** besides the judgement of progression of existing or manifestations of new pulmonary metastasis should in particular focus on signs for pulmonary thromboembolic events, which constitute a reason to stop the application of tTF-NGR in the following cycles and start therapeutic anticoagulation according to the institutional standard even without any clinical symptoms or hemodynamic signs being present.

6.5 Procedures and Variables

6.5.1 Measurements Assessed

The *Time and Events* Table below describes the required evaluations. For simplicity, only cycle 1 and cycle 2 is shown in Table 3 for ARM 1 and Table 4 for ARM 2. However, the following treatment cycles must be conducted in the same way as cycle 2.

6.5.1.1 Radiation exposure

Since the radiological examinations for the study follow common clinical guidelines for the patients with STS, a specific approval of the “Bundesamt für Strahlenschutz” is not necessary.

Table 3: Schedule of evaluation ARM 1.

ARM 1

Procedure	Baseline [1]	Cycle 1 and 2							Tumor assessment[6,9]	End of Treatment[2]
		C1 D1	C1D 8-14[10]	C1D15-21[12]	C2D1	C2D8-14[10]	C2D15-21[12]	C3D1[7]		
Trabectedin administration		X			X			X		
**Visit**	X	X	X	X	X	X	X	X	X	X
**BACKGROUND**										
Informed consent obtainment	X									
Inclusion and exclusion criteria	X									
Demography	X									
Disease History	X									
Medical and surgical history	X									
Previous Medications	X									
Concomitant medications	→	→	→	→	→	→	→	→	→	→[8]
**EXAMINATION**										
Height	X									
Weight	X	X	X	X	X	X	X	X	X	X
BSA calculation		X			X			X		
Vital signs[3]	X	X			X			X	X	X
ECOG performance status	X	X			X			X	X	X
Complete Physical Exam	X	X			X			X	X	X
ECG	X									
Echocardiography	X									
AEs evaluation		→	→	→	→	→	→	→	→	→[8]
Patient reported outcomes	X	X	X	X	X	X	X	X	X	X
**LAB.ASSESSMENTS & PROCEDURES**										
**Troponin T** (or **I**) **hs**[4]	X	X	X[11]	X[11]	X	X[11]	X[11]	X		X
Hematology[4]	X	X	X[11]	X[11]	X	X[11]	X[11]	X		X
Coagulation[4]	X	X	X[11]	X[11]	X	X[11]	X[11]	X		X
Serum Chemistry[4, 5]	X	X	X[11]	X[11]	X	X[11]	X[11]	X		X
Liver function tests[4]	X	X	X[11]	X[11]	X	X[11]	X[11]	X		X
Urinalysis[4]	X	X			X			X		X
Serum pregnancy test[13]	X									X
**TUMOR EVALUATION**										
CT or MRI (see chapter 6.1.1)[6]	X								X[6]	X
Evaluation (see chapter 6.1.1)[6]	X								X[6]	X

[1]Screening/Baseline evaluations must be performecd within 2 weeks prior to the first dose of study drug (see chapter 6.1,1).

[2] End of study treatment visit must be performed approximately 28-35 days after the last drug treatment.

[3] Body temperature, blood pressure (BP), heart rate, respiration rate and pulse oximetry.

[4] If Lab abnormality of CTC Grade ≥3 occurs, the appropriate laboratory test(s) should be performed as clinically indicated until recovery.

[5] In case of CTC Grade ≥3 hypophosphataemia, perform 24 h urine phosphate testing.

[6] Tumor assessment must fulfill **iRECIST criteria** (as described above in chapter 6.1.1, ref. 30) for evaluation of response. Pre-study scans are acceptable if they were taken within 2 weeks prior to study treatment and if they yield sufficient information to serve as baseline exams for subsequent iRECIST evaluations. Tumor response or progression will be evaluated as described in chapter 6.1.1 at regular intervals during the study. **If post-treatment response is noted, it must be confirmed with a repeat scan at least 4 weeks, and ideally, no more than 5 weeks after first declaration of response, with subsequent assessments obtained every other cycle thereafter**. Assessment of tumor response at early withdrawal only if last tumor assessment was performed 3 or more weeks before. The time points for stating progressive disease (PD) follow the rules of iRECIST with definitions such as iUPD and iCPD to be followed (see chapter 6.1.1, ref. 30, see also scenarios in Suppl. Appendix of 30). Tumor assessments are to be continued until iCPD. In case EOT is not determined by iCPD, tumor assessments will go on after EOT until iCPD.

[7] If the patient is eligible to receive further treatment, he can start additional cycles starting again from day 1.

[8] AE and concomitant medications will be recorded up to EOT visit. After EOT, only events and relative concomitant medications that are judged as study treatment or procedures related will be recorded. It is particularly important to record anti-cancer therapies after EOT.

[9] Test results on hematology and serum chemistry obtained up to 3 days prior the visit are acceptable.

[10] At least one visit is obligatory during day 8 - 14. The visit can be virtual and examinations as well as the lab. assessments can be done by a primary care physician, but the local investigators has to ensure that the respective data are transferred to the eCRF correctly.

[11] Determination of the laboratory values including Troponin T or Troponin I hs hs level is not obligatory but should be done, if possible.

[12] Visit not obligatory / can be virtual as decided by local investigator.

[13] Pregnancy test will be performed as described in chapter 6.3.1 at the baseline evaluation, EOT and monthly between C1D1 and EOT. Since one cycle has 21 days, the table shows no crosses during the cycles.

Table 4: Schedule of evaluation ARM 2.

ARM 2

Procedure	Baseline [1]	Cycle 1 and 2											Tumor assessment[6,9]	End of Treatment[2]
		C1D1	C1D2	C1D3	C1D8-14[13]	C1D15-21[16]	C2D1	C2D2	C2D3	C2D8-14[13]	C2D15-21[16]	C3D1[7]		
Trabectedin administration		X					X					X		
tTF-NGR administration[10]			X	X				X	X					
**Visit**	X	X	X	X	X	X	X	X	X	X	X	X	X	X
**BACKGROUND**														
Informed consent obtainment	X													
Inclusion and exclusion criteria	X													
Demography	X													
Disease History	X													
Medical and surgical history	X													
Previous Medications	X													
Concomitant medications	→	→	→	→	→	→	→	→	→	→	→	→	→	→[11]
**EXAMINATION**														
Height	X													
Weight	X	X			X	X	X			X	X	X	X	X
BSA calculation		X					X					X		
Vital signs[3]	X	X	X	X			X	X	X			X	X	X
ECOG performance status	X	X	X	X			X	X	X			X	X	X
Complete Physical Exam	X	X					X					X	X	X
ECG	X												X	
Echocardiography	X												X	
AEs evaluation		→	→	→	→	→	→	→	→	→	→	→	→	→[11]
Patient reported outcomes	X	X	X	X	X	X	X	X	X	X	X	X	X	X
**LAB. ASSESSMENTS & PROCEDURES**														
**Troponin T (I) hs**[4]	X	X	X	X	X[14]	X[14]	X	X	X	X[14]	X[14]	X		X
Hematology[4]	X	X	X	X	X[15]	X[15]	X	X	X	X[15]	X[15]	X		X
Coagulation[4]	X	X			X[15]	X[15]	X			X[15]	X[15]	X		X
Serum Chemistry and CK[4, 5]	X	X	X	X	X[15]	X[15]	X	X	X	X[15]	X[15]	X		X
Liver function tests[4]	X	X	X	X	X[15]	X[15]	X	X	X	X[15]	X[15]	X		X
Urinalysis[4]	X	X					X					X		X
Serum pregnancy test[17]	X													X
**CENTRALIZED LAB. ASSESSMENTS & PROCEDURES**														
HAFA sample (before dosing)		X[8]					X[8]					X[8]	X	X
Pharmacokinetics tTF-NGR[12]			X	X				X	X					
**TUMOR EVALUATION**														
CT or MRI (see chapter 6.1.1)[6]	X												X[6]	X
Evaluation (see chapter 6.1.1)[6]	X												X[6]	X

[1] Screening/Baseline evaluations must be performecd within 2 weeks prior to the first dose of study drug (see chapter 6.1.1).

[2] End of study treatment visit must be performed approximately 28-35 days after the last drug treatment.

[3] Temperature, BP, heart rate, respiration rate and pulse oximetry.

[4] If Lab abnormality of CTC Grade ≥3 occurs, the appropriate laboratory test(s) should be performed as clinically indicated until recovery. Troponin T hs or Troponin I hs has to be measured daily through the cycles at days of tTF-NGR application. It is recommended to measure serum chemistry on D2 of each cycle before tTF-NGR is given to monitor possible trabectedin induced elevated ALT and AST levels.

[5] In case of CTC Grade ≥3 hypophosphataemia, perform 24 h urine phosphate testing.

[6] Tumor assessment must fulfill **iRECIST criteria** (as described above in chapter 6.1.1, ref. 30) for evaluation of response. Pre-study scans are acceptable if they were taken within 2 weeks prior to study treatment and if they yield sufficient information to serve as baseline exams for subsequent iRECIST evaluations. Tumor response or progression will be evaluated as described in chapter 6.1.1 at regular intervals during the study. **If post-treatment response is noted, it must be confirmed with a repeat scan at least 4 weeks, and ideally, no more than 5 weeks after first declaration of response, with subsequent assessments obtained every other cycle thereafter**. Assessment of tumor response at early withdrawal only if last tumor assessment was performed 3 or more weeks before. The time points for stating progressive disease (PD) follow the rules of iRECIST with definitions such as iUPD and iCPD to be followed (see chapter 6.1.1, ref. 30, see also scenarios in Suppl. Appendix of 30). Tumor assessments are to be continued until iCPD. In case EOT is not determined by iCPD, tumor assessments will go on after EOT until iCPD.

[7] If the patient is eligible to receive further treatment, he can start additional cycles starting again from day 1.

[8] Samples for HAFA are to be taken on day 2 of each cycle (table 4 shows day 1) before tTF-NGR administration, tumor assessment visits and at End of Treatment visit (**for details see Laboratory Manual**).

[9] Test results on hematology and serum chemistry obtained up to 3 days prior the visit are acceptable.

[10] exact days should be chosen according to 1st and last tTF-NGR application as specified by the results of the safety run-in cohort. If tTF-NGR is applied on days 2 and 3 of a cycle, checks should be done on those days.

[11] AE and concomitant medications will be recorded up to EOT visit. After EOT, only events and relative concomitant medications that are judged as study treatment or procedures related will be recorded.

[12] Pharmacokinetics (PK): For Full PK (only if additional full PK profils are required; see chapter 6.1.1 only for safety part): Cycle 1-3 at D2 (table 4 shows day 1): 30 min before tTF-NGR dosing and 0, 0.5, 1, 2, 3, 4, 5, 24 hours after start of tTF-NGR dosing and D3 before and after tTF-NGR dosing. For Pop PK (randomized Phase III part): D2-D3 of each cycle before and after tTF-NGR dosing. (f**or details see Laboratory Manual**).

[13] At least one visit is obligatory during day 8 - 14. The visit can be virtual and examinations as well as the lab. assessments can be done by a primary care physician, but the local investigators has to ensure that the respective data are transferred to the eCRF correctly.

[14] Determination of the Troponin T hs or Troponin I hs level is obligatory in cycle 1 and cycle 2. For the following cycles determination of the Troponin T (I) hs level is not obligatory, if Troponin T (I) hs was not >ULN in cycle 1 and cycle 2, but should be done, if possible.

[15] Determination of the laboratory is not obligatory but should be done, if possible.

[16] Visit not obligatory obligatory / can be virtual as decided by local investigator.

[17] Pregnancy test will be performed as described in chapter 6.3.1 at the baseline evaluation, EOT and monthly between C1D1 and EOT. Since one cycle has 21 days, the table shows no crosses during the cycles.

Specific safety remark to Table 4: The investigator and study team are particularly asked to observe the recommendations in chapter 6.4.7. concerning risk mitigation and interventional measures for possible thromboembolic events and increase of troponin T hs or troponin I hs.

6.5.2 Visit Description (as per *Time and Events schedule)*

For simplicity, only cycle 1 and 2 are described in detail. The following cycles must be conducted in the same way as cycle 2. The investigator will be responsible for performing all evaluations and recording information in the medical record as well as completing eCRF. When possible, the same personnel should perform all evaluations for a given patient. The following deviations from the visit schedule are acceptable:

Unscheduled visits

If deemed appropriate for individual patients, it is at the discretion of the investigator to arrange visits in addition to the scheduled visits described in this section. The assessments to be performed during unscheduled visits will be at the discretion of the investigator and will depend on the reason for the unscheduled visit (e.g., AE, tumor assessment).

Scheduled visits

Study visits are scheduled as in-person visits at the study center clinic if not specified otherwise (see Tables 3 and 4). Every effort should be taken to schedule clinic visits on the day stipulated for evaluation. For the tumor assessment visits, a tolerance of +/- 7 days are acceptable. The application of the next therapy cycle can be postponed for up to 21 days due to the following reasons:
1) due to patient's clinical conditions or
2) by the decision of the investigator after discussion with the Coordinating Investigator Prof. Schliemann
However, the tumor assessment visits and TPs with clinical and imaging examinations are to be strictly performed according to the original plan as outlined in chapter 6.1.1. They should not be newly synchronized with the new cycle dates after postponing.

Screening evaluations (Baseline)

After obtaining patient informed consent, screening evaluations and procedures must be performed within 2 weeks prior to the start of therapy.

At the screening visit, the following procedures and assessments will be performed:

- Informed Consent Form signature
- Inclusion / exclusion criteria

- Demographic data
- Medical and surgical history
    - History of cancer
    - History of allergies
    - Any previous disease or surgery
    - Other abnormalities (if indicated)
- Medication history (including date and description of all prior anti-neoplastic therapy)
- Assessment of concomitant medications
- Measurement of height and body weight and full physical examination with vital signs
- Electrocardiogram (ECG) and Echocardiography
- ECOG Performance Status
- Blood sampling for routine safety laboratory examination (hematology, serum chemistry, coagulation and liver functionality)
- Troponin T or I hs
- Urine dip stick analysis for safety laboratory examination
- Serum pregnancy test for women of childbearing potential (according to inclusion criterion 9)
- HIV, Hepatitis B and C tests
- Tumor evaluation (contrast-enhanced MRT or CT for all areas according to normal clinical practice/standard of care) according to RECIST version 1.1. (see 6.1.1). Pre-study assessments are acceptable if they were taken within 2 weeks to the first dose of study drug and if they yield sufficient information for evaluation.

Based on the information obtained from the above assessments (i.e. once all results including laboratory and tumor evaluations are available), it will be decided whether the patient meets all the inclusion/exclusion criteria and therefore can be enrolled into the study and randomized to one of the two arms.

ARM 1 (to be followed for the phase III randomized part)

Cycle 1, Day 1 (C1D1)

- Administration of trabectedin
- Assessment of concomitant medications
- Measurement of body weight and calculation of the body surface area (BSA)
- Full physical examination and vital signs
- ECOG Performance Status
- Recording of adverse events (CTCAE version 5.0)

- Laboratory testing (according to table 3). Test results on hematology and serum chemistry obtained up to 3 days prior to Day 1 are acceptable. Test results must allow for study drug application.
- Troponin T (or I) hs
- Urine dip stick analysis for safety laboratory examination
- PRO (according to 6.5.4)
- *tTF-NGR is NOT given in this arm*

Cycle 1, Day 8-14 (C1D8-14)
At least one visit is obligatory during day 8 - 14. The visit can be virtual.

- Assessment of concomitant medications, including analgesics
- Measurement of body weight
- Laboratory testing (according to table 3). Test results on hematology and serum chemistry obtained up to 3 days prior the visit are acceptable.
- Troponin T (or I) hs (not obligatory, see Table 3)
- Recording of adverse events (CTCAE version 5.0)
- PRO (according to 6.5.4)

Cylce 1, Day 15-21 (C1D15-21)
Visit not obligatory / can be virtual as decided by local investigator.

- Assessment of concomitant medications, including analgesics
- Measurement of body weight
- Laboratory testing (according to table 3). Test results on hematology and serum chemistry obtained up to 3 days prior the visit are acceptable.
- Troponin T (or I) hs (not obligatory, see Table 3)
- Recording of adverse events (CTCAE version 5.0)
- PRO (according to 6.5.4)

Cycle 2, Day 1 (C2D1)

- Administration of trabectedin
- Assessment of concomitant medications, including analgesics
- Measurement of body weight and re-calculation of the body surface area (BSA), if clinically indicated.
- Full physical examination and vital signs
- ECOG Performance Status
- Laboratory testing (according to table 3). Test results on hematology and serum chemistry obtained up to 3 days prior to Day 1 are acceptable. Test results must allow for study drug application.
- Troponin T (or I) hs, daily
- Urine dip stick analysis for safety laboratory examination
- Recording of adverse events (CTCAE version 5.0)

- PRO (according to 6.5.4)

Cycle 2, Day 8-14 (C2D8-14)
At least one visit is obligatory during day 8 - 14. The visit can be virtual.

- Assessment of concomitant medications, including analgesics
- Measurement of body weight
- Laboratory testing (according to table 3). Test results on hematology and serum chemistry obtained up to 3 days prior the visit are acceptable.
- Troponin T (or I) hs (not obligatory, see Table 3)
- Recording of adverse events (CTCAE version 5.0)
- PRO (according to 6.5.4)

Cycle 2, Day 15-21 (C2D15-21)
Visit not obligatory / can be virtual as decided by local investigator.

- Assessment of concomitant medications, including analgesics
- Measurement of body weight
- Laboratory testing (according to table 3). Test results on hematology and serum chemistry obtained up to 3 days prior the visit are acceptable.
- Troponin T (or I) hs (not obligatory, see Table 3)
- Recording of adverse events (CTCAE version 5.0)
- PRO (according to 6.5.4)

Cycle 3, Day 1 (C3D1)
See Cycle 2, Day 1 (C2D1)

For tumor assessment time points (TP) see chapter 6.1.1.

<u>**ARM 2** (to be followed for both parts of the study)</u>

Cycle 1, Day 1 (C1D1)

- Administration of trabectedin (e.g. Monday)
- Administration of tTF-NGR on days 2 and 3 if not defined otherwise by results of the safety cohort
- Assessment of concomitant medications, including analgesics
- Measurement of body weight and calculation of the body surface area (BSA)
- Full physical examination and vital signs
- ECOG Performance Status
- Recording of adverse events (CTCAE version 5.0)
- Laboratory testing (according to table 4). Test results on hematology and serum chemistry obtained up to 3 days prior to Day 1 are acceptable. Test results must allow for study drug application.

- Troponin T(or I) hs, daily during tTF-NGR application
- Urine dip stick analysis for safety laboratory examination
-
- PRO (according to 6.5.4)
- Pharmacokinetics (PK, see Laboratory Manual, centrally evaluated)

Cycle 1, Day 2 (C1D2)

- Administration of tTF-NGR (as specified by the results of the safety cohort)
- Assessment of concomitant medications, including analgesics
- Vital signs
- ECOG Performance Status
- Laboratory testing (according to table 4). Test results must allow for study drug application.
- Troponin T (or I) hs
- Recording of adverse events (CTCAE version 5.0)
- PRO (according to 6.5.4)
- PK
- HAFA

Cylce 1, Day 3 (C1D3)

- Administration of tTF-NGR
- Assessment of concomitant medications, including analgesics
- Vital signs
- ECOG Performance Status
- Laboratory testing (according to table 4). Test results must allow for study drug application.
- Troponin T (or I) hs
- Recording of adverse events (CTCAE version 5.0)
- PRO (according to 6.5.4)
- PK

Cycle 1, Day 8-14 (C1D8-14)
At least one visit is obligatory during day 8 - 14. The visit can be virtual.

- Assessment of concomitant medications, including analgesics
- Measurement of body weight
- Laboratory testing (according to table 4). Test results on hematology and serum chemistry obtained up to 3 days prior the visit are acceptable.
- Troponin T (or I) hs (obligatory in cycle 1 and 2, see Table 4)
- Recording of adverse events (CTCAE version 5.0)
- PRO (according to 6.5.4)

Cylce 1, Day 15-21 (C1D15-21)
Visit not obligatory / can be virtual as decided by local investigator.

- Assessment of concomitant medications, including analgesics
- Measurement of body weight
- Laboratory testing (according to table 4). Test results on hematology and serum chemistry obtained up to 3 days prior the visit are acceptable.
- Troponin T (or I) hs (obligatory in cycle 1 and 2, see Table 4)
- Recording of adverse events (CTCAE version 5.0)
- PRO (according to 6.5.4)

Cycle 2, Day 1 (C2D1)

- Administration of trabectedin
- Assessment of concomitant medications, including analgesics
- Measurement of body weight and re-calculation of the body surface area (BSA), if clinically indicated.
- Full physical examination and vital signs
- ECOG Performance Status
- Laboratory testing (according to table 4). Test results on hematology and serum chemistry obtained up to 3 days prior to Day 1 are acceptable. Test results must allow for study drug application.
- Urine dip stick analysis for safety laboratory examination
- Recording of adverse events (CTCAE version 5.0)
- PRO (according to 6.5.4)
  Pharmacokinetics (PK, see Laboratory Manual)

Cycle 2, Day 2 (C2D2)

- Administration of tTF-NGR
- Assessment of concomitant medications, including analgesics
- Vital signs
- ECOG Performance Status
- Laboratory testing (according to table 4). Test results must allow for study drug application.
- Troponin T (or I) hs
- Recording of adverse events (CTCAE version 5.0)
- PRO (according to 6.5.4)
- PK
- HAFA

Cycle 2, Day 3 (C2D3)
- Administration of tTF-NGR
- Assessment of concomitant medications, including analgesics
- Vital signs

- ECOG Performance Status
- Laboratory testing (according to table 4). Test results must allow for study drug application.
- Troponin T (or I) hs
- Recording of adverse events (CTCAE version 5.0)
- PRO (according to 6.5.4)
- PK

Cycle 2, Day 8-14 (C2D8-14)
At least one visit is obligatory during day 8 - 14. The visit can be virtual.

- Assessment of concomitant medications, including analgesics
- Measurement of body weight
- Laboratory testing (according to table 4). Test results on hematology and serum chemistry obtained up to 3 days prior the visit are acceptable.
- Troponin T (or I) hs (obligatory in cycle 1 and 2, see Table 4)
- Recording of adverse events (CTCAE version 5.0)
- PRO (according to 6.5.4)

Cycle 2, Day 15-21 (C2D15-21)
Visit not obligatory / can be virtual as decided by local investigator.

- Assessment of concomitant medications, including analgesics
- Measurement of body weight
- Laboratory testing (according to table 4). Test results on hematology and serum chemistry obtained up to 3 days prior the visit are acceptable.
- Troponin T (or I) hs (obligatory in cycle 1 and 2, see Table 4)
- Recording of adverse events (CTCAE version 5.0)
- PRO (according to 6.5.4)

Cycle 3, Day 1 (C3D1)
See Cycle 2, Day 1 (C2D1)

For tumor assessment time points (TP) see chapter 6.1.1.

Tumor assessment (Arm 1 and Arm 2)

Tumor assessment for disease progression will be performed as described in chapter 6.1.1.

- Tumor assessment according to iRECIST version 1.1 (see chapter 6.1.1)
- Assessment of concomitant medications, including analgesics
- Measurement of body weight

- Full physical examination and vital signs
- ECOG Performance Status
- ECG and Echocardiography
- Recording of adverse events (CTCAE version 5.0)
- HAFA (only for Arm 2)
- PRO (according to 6.5.4)

End of study Treatment (EOT) Visit (Arm 1 and Arm 2)

Must be performed 28-35 days after last drug treatment.

- Tumor Evaluation according to RECIST version 1.1 (see chapter 6.1.1)
- Assessment of concomitant medications, including analgesics
- Measurement of body weight
- Full physical examination and vital signs
- ECOG Performance Status
- Laboratory testing (hematology, coagulation, liver function, serum chemistry and CK). Test results on hematology and serum chemistry obtained up to 3 days prior the visit are acceptable.
- Troponin T or I hs
- Urine dip stick analysis for safety laboratory examination
- Recording of adverse events (CTCAE version 5.0)
- Serum Pregnancy test (if applicable)
- HAFA (only for Arm 2)
- PRO (according to 6.5.4)

6.5.3 Primary variables

Primary variable for this study is Progression-free survival (PFS) in the tTF-NGR plus trabectedin treatment group (Arm 2) versus trabectedin alone (Arm 1).

PFS is defined as the difference between the date of first progression (iCPD) or death and date of randomization (which represents Day 0 for both arms) assuming as censored the last time where the patients are assessed without progression (progression-free). Patients who are without progression (progression free) will be censored at the time of the last tumor assessment.

iRECIST (30) will be used by central radiology in a blinded fashion for final judgement of therapy efficacy in both arms of the trial. For this purpose after everyassessment of a local investigator pseudonymized tumor imaging data will be sent to the Münster study office immediately. Detailed procedure is given in chapter 6.1.1. The images are checked for complete information in the DVDs and for pseudonymization and are transferred to the central radiology [redacted] to allow for comparative

evaluation in a blinded fashion. Results of the central assessment are transferred back to the Münster study office. Thus, the study office any time during the ongoing trial is informed about cases with divergent progression assessments by a local investigator and the central blinded reviewer. Very few such cases are expected. If a significant number of divergent cases emerge, appropriate measures will be taken. For example, individual cases may be discussed in a pseudonymized fashion in a common meeting of the local investigator and the central blinded reviewer.

6.5.4 Secondary variables

- Efficacy of tTF-NGR plus trabectedin:
  - median PFS in the trabectedin plus tTF-NGR group (Arm 2) versus trabectedin alone (Arm 1).
  - disease control rate (DCR, consisting of CR, PR, and stable disease (SD) for ≥18 weeks)
  - Overall Response Rate (ORR), i.e. rate of CR and PR of tTF-NGR plus trabectedin treatment group (Arm 2) versus trabectedin alone (Arm 1).
  - Overall survival (OS) in the tTF-NGR plus trabectedin treatment group (Arm 2) versus trabectedin alone (Arm 1), assessed at 12 months and 18 months after randomization.
  - Median Overall survival (mOS) in the tTF-NGR plus trabectedin treatment group (Arm 2) versus trabectedin alone (Arm 1).
- Safety of tTF-NGR plus trabectedin:
  - AEs assessment based on CTCAE v.5.0.
  - Standard laboratory (hematology, biochemistry and urinalysis) parameters.
  - Troponin T or I hs
  - Physical examination findings including assessment of vital signs and physical measurements (blood pressure, heart rate, body weight).
- Assessment of the formation of human anti-fusion protein antibodies (HAFA) against tTF-NGR (Arm 2).
- Collection of quality of life data as patient reported outcomes (PRO).

  Patients shall fill out validated EORTC QLQ-C30 questionnaires at all visits. These data shall be transferred to the electronic patient record (eCRF) – to support clinical care – of each site as well as to the study database to enable statistical analysis of patient reported outcomes (PROs). Data transfer shall be compliant with data protection regulations (EU-DSGVO) and provide non-redundant data entry.

6.5.5 Safety variables

The following definitions are based on German Law, Clinical Trial Directive 2001/20/EC and guidelines, and on guidelines of the International Conference on Harmonization (ICH) and on the final rule issued by the Food and Drug Administration (FDA, effective 6 April 1998).

6.5.5.1 Medical history

Any conditions which started before signature of Informed Consent are recorded as medical / surgical history. Any medical occurrence recorded as medical history and worsened after the first treatment should be considered as an AE.

6.5.5.2 Adverse Event

Any untoward medical occurrence in a patient or clinical trial subject administered a medicinal product and which does not necessarily have a causal relationship with this treatment (Directive 2001/20/EC).

All AE assessments will be graded on the CTCAE v.5.0.

Note: An AE can therefore be any unfavorable and unintended sign (including an abnormal laboratory finding), symptom, or disease temporally associated with the use of a medicinal product, whether or not considered related to the investigational medicinal product (CT 3 June 2011).

By definition, for this study, all AEs are regarded as 'treatment emergent', if not seen before treatment or, if already present before treatment, worsened after start of treatment.

Disease-related AEs

Any AE considered related to advanced or metastatic soft tissue sarcoma.

Adverse reaction (AR)

An adverse reaction is any untoward and unintended response to an investigational medicinal product related to any dose administered.
The definition covers also medication errors and uses outside what is foreseen in the protocol, including misuse and abuse of the product.
The definition implies a reasonable possibility of a causal relationship between the event and the investigational medicinal product. This means that there are facts (evidence) or arguments to suggest a causal relationship. (Directive 2001/20/EC, CT 3, June 2011)

For legal reporting purposes, all adverse events judged by either the reporting investigator or the sponsor as having a reasonable causal relationship to a medicinal product qualify as adverse reactions. When the investigator's judgement is missing, the case also qualifies as adverse reaction.

Unexpected adverse reactions

The Coordinating Investigator on behalf of the Sponsor will perform an assessment of expectedness for each serious AR (definition of seriousness see 6.5.5.3). The assessments will be based on the current version of the Investigator's Brochure for tTF-NGR (passage "Reference Safety Information").

An unexpected adverse reaction is defined as an adverse reaction, the nature and severity of which is not consistent with the applicable product information. Reports which add significant information on the specificity, increase of occurrence, or severity of a known, already documented adverse reaction also constitute unexpected events.

Suspected unexpected serious adverse reactions (SUSAR)
A SUSAR is any adverse event that is serious (see 6.5.5.3), qualifies as adverse reaction and has been assessed as unexpected.

Categories for adverse event assessment
All AEs will be assessed and documented by the Investigator according to the categories detailed below.

Severity
The severity of the event will also be assessed using the Common Terminology Criteria for Adverse Events (CTCAE) (see version 5.0). The investigator is to classify the severity of an AE according to the 5-grade CTCAE system.
The term “severe” is used to describe the intensity (severity) of a specific event. This is not the same as “serious,” which is based on patient/event outcome or action criteria.

Study drug action
Any potential study drug action to resolve the AEs is to be documented as follows

- Dose Not Changed
- Dose Reduced
- Drug Increased
- Drug Interrupted
- Drug Withdrawn
- Not Applicable
- Unknown

Concomitant or additional treatment given:

- Yes
- No

Causal relationship to study drug

- Reasonable possibility (meaning that there are facts (evidence) or arguments to suggest a causal relationship)
- No reasonable possiblity

Factors to be considered in assessing the relationship of the AE to study drug include:

- The temporal sequence from drug administration: the event should occur after the drug is given. The length of time from drug exposure to event should be evaluated in the clinical context of the event.

- Recovery on discontinuation (de-challenge), recurrence on reintroduction (re-challenge): Patient’s response after drug discontinuation (de-challenge) or patients response after drug re-introduction (re-challenge) should be considered in the view of the usual clinical course of the event in question.
- Underlying, concomitant, intercurrent diseases: Each report should be evaluated in the context of the natural history and course of the disease being treated and any other disease the patient may have.
- Concomitant medication or treatment: The other drugs the patient is taking or the treatment the patient receives should be examined to determine whether any of them may be suspected to cause the event in question.
- Known response pattern for this class of drug: Clinical/preclinical
- The pharmacology and pharmacokinetics of the test drug: The pharmacokinetic properties (absorption, distribution, metabolism and excretion) of the test drug(s), coupled with the individual patient’s pharmacodynamics should be considered.

Please refer to the eCRF instructions for completion of the Adverse Event CRF module.

Note: The investigator must answer the question regarding the causal relationship for each (anti-cancer) therapy received by the patient. If a causality assessment cannot be made for each (anti-cancer) therapy separately, the assessment should be made for the combination treatment.
For events which span more than one assessment period / cycle, the causality assessment should be the same.

Outcome

The outcome of the AE is to be documented as follows:

1. Recovered / resolved
2. Recovering / resolving
3. Not recovered / not resolved
4. Recovered / resolved with sequelae
5. Fatal
6. Unknown

Assessments and documentation of adverse events

In addition to the information previously requested the following items have to be completed:

- ID event number: identifies univocally the event along the study. If an event undergoes a change of severity and/or seriousness it should be reported with same id number as the previously recorded.
- MedDRA code: the sponsor must codify the event using the MedDRA dictionary, the last updated version.
- Start date:
    - For a new event is the onset date.

  - For a previously reported event is the date when change of severity and/or seriousness occurred.
- Stop date is the date when the AE ends.

6.5.5.3 Serious Adverse Events

The following SAE definition is based on ICH guidelines and applicable law.
A SAE is classified as any untoward medical occurrence that at any dose:

1. Results in death, or
2. Is life threatening, or
3. Requires in subject hospitalization or prolongation of existing hospitalization, or
4. Results in persistent or significant disability / incapacity, or
5. Is a congenital anomaly / birth defect or
6. Is an important medical event.

The term 'life threatening' in the definition refers to an event in which the subject was at risk of death at the time of the event, it does not refer to an event, which hypothetically might have caused death if it were more severe.
Medical and scientific judgment should be exercised in deciding whether it is appropriate to report an AE as serious also in other situations, such as important medical events that may not be immediately life threatening or result in death or hospitalization but may jeopardize the subject or may require intervention to prevent one of the other outcomes listed in the definition above. These should also usually be considered serious. Examples of such events are intensive treatment in an emergency room or at home for allergic bronchospasm; blood dyscrasias or convulsions that do not result in subject hospitalization; or development of drug dependency or drug abuse. Any suspicion of a thromboembolic event has to be considered as important medical event and therefore as serious, if no other criterion applies.

Hospitalization: Any AE leading to hospitalization or prolongation of hospitalization will be considered as Serious, UNLESS at least one of the following exceptions are met:

- The admission results in a hospital stay of less than 12 hours.
  or
- The admission is not associated with an AE (e.g. social hospitalization for purposes of respite care).

However, it should be noted that an AE requiring invasive treatment during any hospitalization may fulfill the criteria of 'medically important' and as such may be reportable as a SAE dependent on clinical judgment. In addition, where local regulatory authorities specifically require a more stringent definition, the local regulation takes precedent.

Disability: A substantial disruption of a person's ability to conduct normal life's functions. The investigator should take appropriate diagnostic and therapeutic measures to minimize the risk to the subject. Where appropriate, he/she should take diagnostic measures to collect evidence for clarification of the relationship between the SAE and study drug.

Pregnancy: Pregnancy is not serious by itself. However, in the rare case that a female subject participating in this clinical trial becomes pregnant after having taken trial medication, the Investigator must report immediately (within 24 hours) the drug exposure during pregnancy (DEDP) to the Sponsor using the SAE form. The outcome of the pregnancy associated with the drug exposure during pregnancy should be followed up and reported to the Sponsor. Follow-up will be done using specific additional questionnaires, supplied by the Safety Desk if needed. Information will be collected as far as covered by consent (information about a child needs to be covered by consent of the patient's partner, too). Any reasonable effort will be made to collect additional consent.

Observation period

For each individual subject, the period for event observation starts with the signing of the informed consent form. In principle, the AE observation period stops with the termination of the End of Treatment visit; however, follow-up information and outcome of prior SAEs must be provided after the End of Treatment visit as available. **Most important according to EMA guidelines** is the AE reporting at 3 months, 6 months, and 1 year to facilitate safety regulatory safety assessment. In addition, if the investigator gains knowledge of an AE after withdrawal withdrawal or after the End of Treatment visit and considers that event drug related, the event has to be reported to the Sponsor.

Subjects who permanently discontinue administration of study drugs due to an AR, SAE, abnormal laboratory value, or any other reason should be followed for at least 30 days following the last dose of study drugs. In case of SAE, patients should be followed up for final outcome until resolution or if resolution becomes unlikely until stabilization, or until death.

Procedure

The investigator must submit a complete SAE Report for all SAE, regardless of a possible causal relationship, to the sponsor´s Safety Desk within 24 hours of awareness of the event.

This report has to be sent to the Zentrum für Klinische Studien (ZKS) in the Faculty of Medicine/UKM Münster using the SAE Report Form provided, duly fulfilled and signed by the Investigator.

The SAE form should be completed with as much information as possible. The investigator should not wait for full details before making the initial report.

Minimum information to be included in any initial report:

1. Trial patient number
2. SAE details
3. Details about administration of investigational medicinal product
4. Causality assessment of SAE to investigational medicinal
5. Reporting investigator

The investigator will also inform the Safety Desk of relevant follow up information and the outcome of the SAE as soon as possible using the SAE form. The Investigator is required to document the full course of SAE and any therapy given, including any relevant findings/records in the report.
SAE reporting has to be done within 24 hours of knowledge by faxing the SAE form to the sponsor's Safety Desk. Personal data have to be replaced by the trial patient number before forwarding any information.

**Safety Desk Contact**
**Zentrum für Klinische Studien (ZKS) Münster**<br>**Von-Esmarch-Straße 62**<br>**48129 Münster**<br>**Germany**<br>**Fax:** [redacted]<br>**Phone:** [redacted]<br>**E-Mail** [redacted]

The investigator should answer any query sent by the Safety Desk as soon as possible. In case of death of a study participant, the investigator also has to supply the competent authority and the Ethics Committee with any details, if requested by them.

Assessment of serious adverse events by the sponsor
The Safety Desk will document each SAE, check it and query additionally required information, if any. The Coordinating Investigator will review each SAE again for seriousness and relatedness. The Coordinating Investigator will also assess whether an SAR is expected or unexpected (SUSAR) according to the applicable Product Information (see above under "Unexpected Adverse Reactions"), and whether any SAE might influence the benefit-risk-ratio or might require changes in the conduct of the trial.

Legal reporting requirements of the sponsor
It is the duty of the Safety Desk to ensure that Ethics Committee/s, competent authority/ies and participating investigators are informed of all suspected unexpected serious adverse reactions (SUSARs) in accordance with legal requirements (fatal or life threatening SUSARs immediately, by the latest within 7 days, detailed follow-up

information within an additional 8 days, if any; all other SUSARs immediately, by the latest within 15 days). SUSAR follow-up reports will be submitted, if appropriate.

The Safety Desk will observe SUSAR cross reporting obligations with other trials of the same sponsor investigating any of the same active substances, if any.

The Coordinating Investigator is responsible for the ongoing safety evaluation of the trial. The Safety Desk and the Coordinating Investigator will inform each other immediately about any relevant safety information coming to their knowledge. In case of safety relevant issues (besides SUSARs) which require expedited reporting, the Safety Desk will support the Coordinating Investigator in submitting an appropriate report in due time. This includes issues which might materially alter the current benefit-risk assessment of the investigational medicinal product or that would be sufficient to consider changes in the investigational medicinal product's administration or in the overall conduct of the trial as well as urgent safety measures to protect the subjects against any immediate hazard.

Annual safety reports will be prepared and submitted in accordance with legal requirements (Development Safety Update Report, DSUR). The Coordinating Investigator is responsible for providing the updated benefit-risk assessment of the trial for the Report and passages requiring medical assessment. The Safety Desk is responsible for preparing all other parts of the Annual Safety Report, finalizing it and submitting it. The report will be prepared annually from the date of the initial Clinical Trial Authorisation and submitted within 60 days of data lock point (day before anniversary of initial authorisation). Additional reports will be prepared, if requested by the competent authority and Ethics committee.

Details of all AEs will be reported to the competent authority, if requested.

The Safety Desk will provide information for the Data and Safety Monitoring Board (DSMB) on request.

6.5.5.4 Physical Examination

Physical examinations will be performed according to the schedule.
At screening the investigator will perform a full examination, including the following:

General appearance	Breast	Abdomen
Skin	Head and neck	Lymph nodes
Eyes	Lungs	Musculoskeletal system (incl. extremities and spine)
Ears, nose and throat	Heart	

At all other visits the extent of the physical examination will be at the discretion of the investigator. Depending on criteria of relative timing, the findings of the physical examination are to be recorded as medical/surgical history or as an AE.

Vital signs

Vital signs as scheduled during this study will include the following measurements:

- Body temperature
- Heart rate (for 1 min, after 3 minutes in sitting position)
- Systolic and diastolic blood pressure (after 3 minutes in sitting position)

Blood pressure monitoring

Blood pressure will be monitored at each drug administration:
(1) during assessment procedure
Arm 1:
(2) immediately prior to start of trabectedin administration
(3) after end of administration (+/- 5 minutes)
(4) 1 hour after end of infusion (± 10 minutes)

Arm 2:
(2) immediately prior to start of trabectedin administration
(3) after end of trabectedin administration (+/-5 minutes)
(4) immediately prior tTF-NGR administration
(5) every 30 minutes (± 5 minutes) from start of tTF-NGR administration on
(6) immediately after end of administration (+/- 5 minutes)
(7) 30 minutes after end of infusion (± 5 minutes)

12-lead electrocardiogram (ECG)

Standard 12-lead ECGs will be obtained. Chest wall and limb leads will be recorded. The following variables will be recorded and analyzed: heart rate, P wave, and standard intervals (PR, QRS, QT, and QTc).

Echocardiography

An echochardiography will be performed in order to determine if the heart's left and right ventricles are functioning properly and to diagnose abnormalities in the heart wall. The ejection fraction will be determined.

6.5.5.5 Clinical Laboratory Tests

Standard laboratory examinations will be performed locally at the individual study center.

Each sample for standard laboratory blood examination will be approximately 10 to 15 mL. The maximum volume of blood to be drawn during a single day is approximately 60 mL.
As indicated below, additional laboratory tests will be performed if medically indicated. Non-fasting blood samples and urine specimens will be analyzed.
If evaluations related to fasting conditions (*e.g.,* glucose) are clinically significantly altered in the non-fasting blood or urine samples, re-examinations will be done under fasting conditions.

Table 5: Laboratory evaluation for standard safety and tumor assessment

Standard laboratory evaluations

**Serum chemistry**	- Creatinine	- Chloride	- Calcium
	- BUN		- Glucose
	- Potassium	- Total Protein	- Phosphorus
	- Sodium	- Albumin	- Magnesium
	- Uric acid		- LDH
**Hematology**	- WBC	- Hematocrit	- Hemoglobin
	- ANC	- Platelet count	- Differential count (5-part)
	- RBC		
**Coagulation**	- Quick/INR	- PTT	D-Dimer
	- Fibrinogen		
**Cardiac**	- CK	- **Troponin T or I hs**	
**Liver function tests**	- ALT (GPT)	- AP	- Total bilirubin
	- AST (GOT)	- γGT	
**Urinalysis (dipstick)**	- Glucose	- Leukocytes	- Protein
	- pH	- Erythrocytes	- Hemoglobin

Selected laboratory variables will be graded according to the CTCAE laboratory toxicity criteria (see CTCAE v.5.0). NOTE: CTCAE 5.0 does not specify Troponin T hs, but only cardiac troponins T and I. Nevertheless, it is important to measure Troponin T hs, as this parameter was defining DLT and bears the potential to be a safety biomarker preventing clinically relevant side effects of tTF-NGR (see IB and chapter 6.1.4 of this protocol).
Abnormalities in clinical laboratory tests that are considered clinically significant by the investigators will be recorded on the “Adverse Events” CRF.

<u>Laboratory Assessments</u>
Before the study, the investigator will supply the sponsor with a list of the normal ranges and units of measurement for the laboratory variables to be determined during the study at the site. All abnormal laboratory values require a comment on the laboratory report, regardless of the clinical significance.

Clinical Laboratory Tests

After reviewing the laboratory report and evaluating any results that are outside the normal range, the investigator must sign and date the laboratory report. Any abnormal laboratory test result that warrants further investigation to guard the patient's safety will be repeated as appropriate (no later than the next scheduled study visit) and reviewed by the investigator.

6.5.6 Efficacy variables

6.5.6.1 Tumor assessment

Anti-cancer activity (iRECIST modification) will be assessed (clinically and imaging) at week 9 and then every 9 weeks (adjusted to cycle length of 3 weeks). Decision on application of next cycle versus EOT is described in chapter 6.1.1.

Overall Response is assessed in analogy to RECIST criteria version vs 1.1:

- Complete Response (CR): Disappearance of all target lesions. Any pathological lymph nodes (whether target or non-target) must have reduction in short axis to <10 mm.
- Partial Response (PR): At least a 30% decrease in the sum of diameters of target lesions, using the baseline sum diameters as reference.
- Progressive Disease (PD): At least a 20% increase in the sum of diameters of target lesions, using the smallest sum in the study as reference (this includes the baseline sum if that is the smallest in the study). In addition to the relative increase of 20%, the sum must also demonstrate an absolute increase of at least 5 mm. (*Note*: the appearance of one or more new lesions is also considered progression, **the rules as described in chapter 6.1.1 must be observed before a progression can be used for ending the treatment in a patient**.
- Stable Disease (SD): Neither sufficient shrinkage to qualify for PR nor sufficient increase to qualify for PD, using the smallest sum diameters as reference in the study.

Table 6: Time point Response according to RECIST version 1.1.

Target lesions	Non-target lesions	New lesions	Overall response
CR	CR	No	CR
CR	Non-CR/non-PD	No	PR
CR	Not evaluated	No	PR
PR	Non-PD or not all evaluated	No	PR
SD	Non-PD or not all evaluated	No	SD
Not all evaluated	Non-PD	No	NE
PD	Any	Yes or No	PD
Any	PD	Yes or No	PD
Any	Any	Yes	PD

CR = complete response, PR = partial response, SD = stable disease, PD = progressive disease, and NE = inevaluable.

Lymph nodes identified as target lesions should always have the actual short axis measurement recorded (measured in the same anatomical plane as the baseline examination), even if the nodes regress to below 10 mm in the study. This means that when lymph nodes are included as target lesions, the 'sum' of lesions may not be zero even if CR criteria are met, since a normal lymph node is defined as having a short axis of <10 mm. eCRFs or other data collection methods may therefore be designed to have target nodal lesions recorded in a separate section where, in order to qualify for CR, each node must achieve a short axis <10 mm. For PR, SD and PD, the actual short axis measurement of the nodes is to be included in the sum of target lesions.

6.5.6.2 Median Progression-Free Survival (mPFS) and Progression-Free Survival Rates and Disease Control rate (DCR)

The PFS time will be assessed for all randomized subjects. The duration is defined beginning from randomization to progression (iCPD) or death from any cause. The PFS rate according to iRECIST will be assessed at week 18 (after 6 cycles), then every 9 weeks (adjusted to cycle length of 3 weeks) thereafter. The DCR, consisting of CR, PR, and stable disease (SD) will be assessed for the time ≥18 weeks.

6.5.6.3 Median Overall Survival (mOS) and Overall Survival Rates

The OS time will be assessed for all randomized patients. The duration is defined beginning from randomization to death from any cause. The median OS time and the OS rate at 12 and 18 months will be calculated. Thereafter the survival of the patients will be monitoired during 3-monthly regular follow-up visits.

iRECIST (30) will be used by central radiology in a blinded fashion for judgement of therapy efficacy in both arms of the trial (see above, chapter 6.5.3).

6.5.7 Other variables and procedures

6.5.7.1 Biomarker: Predictive Marker CD13

To evaluate predictive parameters in this study, central CD13 staining (IHC) will be performed before patient´s entry on trial in the Gerhard Domagk Institute for Pathology at UKM according to a method described (20). CD13 histology can predict prognosis (29) and thus is a routine test and not part of the study. Sample (pathology block material) shipment by study centers has to follow local procedures.
Adress for sending in material:

[redacted]

6.5.7.2 Human anti-fusion protein antibody (HAFA) testing

HAFA testing must be performed on study patients according to details below. A specific manual (**Laboratory Manual**) will be provided to the study site explaining procedures for blood sampling, sample preparation, storage and shipment to study laboratories (UKM). Analysis will be performed using validated methods. Characteristics of the methods will be described in the bioanalytical report and will be referenced in the clinical study report. Analyses will be performed at, or delegated from, the sponsor.

Blood sampling for the assessment of HAFA to tTF-NGR will be taken at Day 2of each cycle prior to dosing in order to avoid potential interference with the assays. Furthermore, samples will be taken at EOT / tumor assessment visit according to table 7.
Approximately 5 mL of blood (total of 40 mL, assuming 6 treatment cycles) will be collected in a blood serum tube for determination of HAFA. The serum samples will be prepared at the study site and then stored at -80°C until shipment to the study laboratories (UKM).

Table 7: HAFA blood sample collection time points.

HAFA Sample ID	Timepoint	Total blood volume withdrawn (mL)
HAFA.1	Cycle 1 Day 2<br>(before administration of tTF-NGR)	5
HAFA.2	Cycle 2 Day 2<br>(before administration of tTF-NGR)	5
HAFA.3	Cycle 3 Day 2<br>(before administration of tTF-NGR)	5
HAFA.4	Cycle 4 Day 2<br>(before administration of tTF-NGR)	5
HAFA.5	Cycle 5 Day 2<br>(before administration of tTF-NGR)	5
HAFA.6	Cycle 6 Day 2<br>(before administration of tTF-NGR)	5
HAFA.7	End of treatment	5
HAFA.8	Tumor assessment visit	5

6.5.7.3 Pharmacokinetics (PK)

PK testing must be performed on study patients according to the **Laboratory Manual** and Table 4. The Laboratory Manual will be provided to the study site explaining procedures for blood sampling, sample preparation, storage and shipment to study laboratories (UKM). Analysis will be performed using validated methods. Characteristics of the methods will be described in the bioanalytical report and will be referenced in the clinical study report. Analyses will be performed at, or delegated from, the central study office.

The full pharmacokinetics (full PK) characterization will be performed in the safety part including 6 patients (for details see Laboratory Manual). If full PK profiles were not determined for 6 patients in the safety part, the full PK characterization is to be continued with additional patients in Arm 2 of the randomized part until full PK characterization has been completed for 6 patients.

Study of population pharmacokinetics (pop PK) will be performed centrally at the time points described in Table 4 (Arm 2) with a validated method as described in the IB (attached).

Blood sampling for the assessment of PK will be taken as described in the Laboratory Manual. Approximately 2.5 mL of blood will be collected in a blood tube for citrated blood for determination of PK. The plasma samples will be prepared at the study site and then stored at -80°C until shipment to the study laboratories (UKM).

6.6 Study Monitoring, Auditis, Inspections and Documentation

Investigators and/or their study staff will be trained at the latest during the initiation visit. During each monitoring visit source data verification will be performed by qualified staff representing the sponsor. A eCRF supplied by the sponsor will be completed for each patient. The entries will be checked by trained delegates of the sponsor.
Monitoring and auditing procedures of the sponsor will be followed, in order to comply with GCP guidelines and to ensure validity of the study data.

6.6.1 Study Monitoring

The objectives of the monitoring procedures are to ensure that the study safety and rights of the study subjects as a study participant are respected and that accurate, valid and complete data are collected, and that the study is conducted in accordance with the study protocol, the principles of GCP and local legislation.

The clinical study sites will be monitored by regular site visits to the investigator by qualified staff representing the sponsor, or their delegate, following internal SOPs of the Zentrum für Klinische Studien (ZKS) Münster. The exact extent of the monitoring procedures is described in a separate monitoring manual. By frequent communication, the site monitor will ensure that the study is conducted according to the protocol. The monitor has the responsibility to treat all information confidentially and to safeguard the integrity and personal privacy of the study participants.

eCRF and all original data collected at the site should be available for review during monitoring visits. The Investigators agree to allow the monitor direct access to all relevant documents and to allocate his/her time and the time of his/her staff to the monitor to discuss findings and any relevant issues. During these visits, the site monitor should review drug accountability records and document retention including the ISF. Additionally, the site monitor should check that clinical study procedures are observed and discuss any problems with the investigator.

6.6.2 Source Data Verification

Inspection and examination of eCRF and source documents (all original recordings, laboratory reports, medical records) - giving due consideration to data protection and medical confidentiality - will be undertaken by representatives of the sponsor. All data recorded in the eCRF must be verified by checking eCRF entries against source documents in order to ensure that the data have been completely and accurately reported as required by the study protocol. Source data verification will be performed and recorded following ZKS´s internal SOP. The patient or the patient’s legally acceptable representative must also allow access to the patient’s medical records. Each patient, or the patient’s legally acceptable representative, will be informed of this prior to the start of the study.

For the purpose of ensuring compliance with the study protocol, the principles of GCP and local legislation, the sponsor may initiate three audits at any study site during the study and after completion.
During or after the clinical study, the regulatory authorities, the EC and/or representatives of the sponsor may request the direct access to all source documents, eCRF and other study documentation for on-site audit or inspection.

6.7 Data Management and DSMB

6.7.1 Data Handling Procedures

The investigator has ultimate responsibility for the collection and reporting of all clinical, safety, and laboratory data entered on the eCRF and any other data collection forms (source documents) and ensuring that they are accurate, authentic/original, attributable, complete, consistent, legible, timely (contemporaneous), enduring, and available when required.

The eCRF includes the capability to record who entered or generated the data and when it was entered or generated. Changes to the data must not obscure the original entry, and must record who made the change, when, and why.
Data reported in the eCRF that are derived from source documents, should be consistent with the source documents or the discrepancies should be explained.

6.7.2 Record Retention

To enable evaluations and/or audits from regulatory authorities or sponsor, the investigator agrees to keep records, including the identity of all participating subjects (sufficient information to link records, eg, eCRFs and hospital records), all original signed informed consent documents, safety reporting forms, source documents, detailed records of treatment disposition, and adequate documentation of relevant correspondence (e.g., letters, meeting minutes, and telephone call reports). The records should be retained by the investigator according to the ICH guidelines, according to local regulations, or as specified in the clinical study agreement, whichever is longer.

If the investigator becomes unable for any reason to continue to retain study records for the required period (eg, retirement, relocation), the Sponsor, the Study Center, and the ZKS should be prospectively notified. The study records must be transferred to a designee acceptable to sponsor.

6.7.3 Data Protection

All participants of the study and the ZKS as the responsible for data management respect the patients' rights to privacy and will ensure the confidentiality of their medical information in accordance with all applicable laws and regulations.

Data management will be done in accordance with applicable rules on data protection.

6.7.4 DSMB

A Data Safety and Monitoring Board (DSMB) is established according to EMA guideline Doc. Ref. EMEA/CHMP/EWP/5872/03 Corr. The DSMB consists of 3 physicians, all being oncologists and experienced in the conduct of clinical studies, 1 of those with specific experienced in ethical questions, and an independent statistician. The DSMB is meeting and working according to a specific charter. Open parts of the sessions can be joined the study statistician Dr. rer. nat. J. Gerß, the Coordinating Investigator Prof. C. Schliemann, representatives of the sponsor, representatives of ANTUREC Pharmaceuticals GmbH. To protect data integrity and unbiased randomization and study conduct the Coordinating Investigator, representatives of the sponsor and representatives of ANTUREC Pharmaceuticals GmbH will leave the meeting as soon as efficacy data are disclosed. After an open part of the session, the DSMB will hold a closed part of the session with only the DSMB members attending before final recommendations are formulated for the sponsor. After the closed session, the meeting will be closed upon discussion of all relevant aspects of the study at this point of time. The DSMB members will have access to unblinded data of the study whenever it is requested.

6.8 Changes in the Conduct of the Study or Planned Analysis

Planned changes in the conduct of the study will be described in protocol amendments; changes in the planned analysis will be described in the clinical study report.

An amendment is a written description of change(s) to, or formal clarification of, a study protocol.

The EC must be informed of all amendments and if necessary prior review and documented approval/favorable opinion must be sought for ethical aspects. Approval must also be obtained from the authorities, if necessary.

Such amendment will be agreed upon by the sponsor, the investigator, the EC and authorities if necessary prior to implementation.

6.9 Statistical Methods and Determination of Sample Size

Statistical analyses will be performed according to the principles of the ICH guideline E9 “Statistical Principles for Clinical Trials” using standard statistical software (e.g. SAS).

6.9.1 Statistical Methods

Statistical analysis plan (SAP)

A statistical analysis plan (SAP) is a document that contains a detailed technical elaboration of the preparation of data for the statistical analysis, as well as detailed procedures for executing the statistical analysis of the primary and secondary endpoints and other data.

Randomization

Randomization is a process by which subjects are allocated to trial groups by chance, which essentially eliminates selection bias. Permuted block randomization with treatment allocation ratio 1:1 will be applied using a suitable software (e.g. randomizer.at). Randomization will be stratified by CD13 positivity grades 1/2 versus CD13 positivity grade 3 (highest grade as given by GDI) and number of chemotherapy regimen before entry on trial: 1 versus >1. Each open study site will get access to the software (login data) and will be informed automatically via e-mail to which arm a new patient was assigned. The study management will receive these e-mails as well and save the data in pdf format.

Analysis populations

The following analysis populations will be considered.

1. **Primary Analysis Population**: all patients enrolled and randomized to one of the two treatment arms (full analysis set). Patients not evaluable for efficacy after randomization and patients lost to follow-up during the study will be considered as censored at the last available assessment date.
2. **Safety Population:** all patients enrolled and randomized to one of the two treatment arms and who received at least one injection.
3. **Per Protocol Population**: all patients in the primary analysis population who completed the treatment (1 cycle) without protocol violation concerning treatment and tumor assessment. Possible protocol violations include concomitant therapies and medications that are not permitted.

Analysis of Demographic and Baseline Characteristics

Patient demographics and baseline characteristics will be analyzed and summarized with descriptive statistics.

Efficacy analysis

Statistical efficacy analyses will generally be performed in the Primary Analysis Population and in the Per Protocol Population. Missing efficacy data will not be replaced applying any kind of statistical imputation.

Descriptive statistics (frequency distribution, mean, standard deviation etc.) will be reported, including the number of treatment cycles.

The primary endpoint is progression-free survival (PFS) according to iRECIST as judged by central blinded radiology. Each patient's PFS will be calculated as the time from randomization to the first occurrence of progression or death. Patients who are progression-free will be censored at the time of the last tumor assessment. In the primary statistical analysis of the phase III randomized part, PFS will be compared between the two randomized treatment groups (Arm 1 versus Arm 2). The primary

statistical analysis will be performed in the Primary Analysis Population according to the intention-to-treat principle. A stratified log-rank test with stratification according to the randomization will be applied (two-sided significance level 5%, power 80%) that provides confirmatory statistical evidence. PFS will be depicted and median progression-free survival (mPFS) will be estimated using the Kaplan-Meier method.

In particular, even if the assessment of PFS by a local investigator differs from the central blinded assessment, the latter assessment is considered the relevant primary efficacy endpoint. In terms of the ICH E9 Addendum (EMA/CHMP/ICH/436221/2017; ICH E9 (R1) addendum on estimands and sensitivity analysis in clinical trials to the guideline on statistical principles for clinical trials), a “treatment policy strategy” is pursued. That means if for example, upon a diagnosed progression of a local investigator a patient’s therapy is changed, whereas central blinded assessment still diagnoses stable disease, in the primary statistical analysis only the central blinded assessment is considered relevant, disregarding the local investigator’s assessment and change of therapy. In addition to this primary “treatment policy” approach, the following supplementary analyses will be performed in order to compare the randomized treatment arms.

- Evaluation of centrally blinded assessed PFS, censored at week 18, disregarding the local investigators‘ assessments
- Evaluation of local investigator assessed PFS, disregarding the centrally blinded assessments
- Evaluation of a combined endpoint of local investigator assessed PFS and centrally blinded assessed PFS (whatever occurs first)
- Competing risk analysis with the primary endpoint centrally blinded assessed PFS, adjusting for the competing risk of a change of therapy after a local investigator diagnosed a progression

The secondary endpoint overall survival (OS) is defined as the time from randomization to death of any cause. In order to compare the two treatment groups with respect to OS, a stratified log-rank test with stratification according to the randomization will be used. OS will be depicted and median overall survival (mOS) will be estimated using the Kaplan-Meier method.

The overall response rate (ORR, consisting of CR and PR) according to iRECIST based on RECIST v1.1 will be calculated at each tumor assessment as well as the best overall response rate across all assessments (BORR). ORR and BORR will be reported for the treatment groups with 95% confidence intervals.

The disease control rate (DCR, consisting of CR, PR, and stable disease (SD) for >18 weeks) will be reported for the treatment groups with 95% confidence intervals.

Prespecified subgroup analyses will be performed with respect to stratification (CD13 1+/2+ versus CD13 3+, number of chemotherapy regimen before entry on trial: 1 versus >1), liposarcoma and leiomyosarcoma (L-sarcoma) versus other type sarcomas (OTS), FNCLCC grade (2 versus 3), and ECOG performance status (ECOG

PS 0 versus PS 1). Further exploratory analyses will be performed for other relevant treatment variables (e.g. sex, age, number of treatment cycles). P-values will not be adjusted for multiple testing and will be interpreted in terms of exploratory signal detection.

Analysis of Safety Criteria

Safety data will be evaluated and summarized descriptively in the safety population. Missing data will not be replaced applying any kind of statistical imputation.

Safety will be assessed through physical examinations, (changes in) vital signs, (changes in) laboratory values (including serum chemistry, hematology parameters) and the recording of Adverse Events (AEs) based on CTCAE version 5.0. Treatment emergent AEs and abnormal laboratory values will be summarized by CTCAE grade (version 5.0) case-wise as well as patient-wise (worst grade per patient).

The toxicity, safety and tolerability of the experimental treatment, the frequency of early treatment termination, early study termination (18 months), and early treatment termination due to AEs will be reported.

Interim Analyses

No Interim Analyis is planned.

6.9.2 Determination of Sample Size

The timing of the study will be event-driven. The primary statistical analysis will be performed at the time when 106 events in terms of PFS have been observed in total across both treatment groups. This required number of events results from the following sample size calculation. Based on the observed PFS in previous trials, PFS is expected to be exponential, and the expected median PFS is 4.6 months in the control arm (Arm 1) and 8 months in the experimental arm (Arm 2). The corresponding expected hazard ratio is HR=0.575. In a 36 months enrolment period with a rate of 3-4 patients per month, 126 patients will be recruited and randomized. The drop-out process after randomization is expected to be exponential with an up to 25% cumulative drop-out rate at month 24 (competing with the survival process). Follow-up after the last randomized patient is planned to be 12 months, after which it is expected that the required number of 106 events in terms of PFS will have occurred. Sample size calculation was performed using the ADDPLAN® software.

6.10 Documentation of Study Findings

Patient data will be collected using a web-based electronic case record form (eCRF) managed by ZKS Münster. Data required according to this protocol are to be recorded in the medical record as a source document and then transcribed to the eCRF as soon as possible. Patient data will be entered into the eCRF by the Investigator who can delegate other designated persons of his/her study staff. Paper CRF may be used only in case of technical difficulties with the electronic CRF.

A reasonable explanation must be given by the investigator for all missing data. As part of the conduct of the trial, the sponsor may have questions about the data documented in the eCRF. These questions will be documented using the electronic data capture system (eCRF) and should be answered by the investigator in a timely manner.

Instructions and procedures on how to use web-based eCRF electronic system will be provided by the sponsor in a dedicated training.

6.11 Record Retention Period

Sponsor retains all study records in accordance with the applicable regulations (GCP-Verordnung §13(10)) for a period of 20 years after the end of the study.

6.12 Use of Information and Publication

Publication of the results of every study is obligatory. After completion of the biostatistical evaluation, the coordinating investigator prepares a study report. The report includes all trial results, irrespective of whether favorable or not. It is signed by him and the person who is responsible for the evaluation. Within 12 months after the end of the study, the coordinating investigator submits a summary of the final report to the Ethics committee and to the competent federal authority. The summary is prepared in the format provided for report synopses by the ICH Guideline E3 “Guidance on Structure and Content of Clinical Study Reports”. For assuring completeness and accuracy of publication and report, the Coordinating Investigator will have complete access to all study data on file at ZKS. As some of the information concerning tTF-NGR and the development activities may be of strictly confidential nature, any publication manuscript (including conference contributions, posters, etc.) must first be reviewed by the Coordinating Investigator and compliance with any legal and contractual confidentiality obligations has to be made sure before its submission or presentation.

Patients’ anonymity will always be protected to the extent agreed upon in the “Patienteninformation” and “Einwilligungserklärung” of used for this study in all publications and presentations that result from this research.

Further details are included in the contracts between the sponsor and the individual investigators.

7 REFERENCE LIST

1. Casali PG, Blay JY. Soft tissue sarcomas: ESMO Clinical Practice Guidelines for diagnosis, treatment and follow-up. Ann. Oncol. 21 Suppl 5: v198-203, 2010.

2. Clark MA, Fisher C, Judson I, J. M. Thomas JM. "Soft-tissue sarcomas in adults." The New Engl. J. Med. 353(7): 701-711, 2005.

3. Riedel RF. Systemic therapy for advanced soft tissue sarcomas: highlighting novel therapies and treatment approaches. Cancer 118(6): 1474-1485, 2012.

4. Kessler T, Bieker R, Padro T, Schwöppe C, Persigehl T, Bremer C, Kreuter M, Berdel WE*, Mesters RM*: Inhibition of tumor growth by RGD peptide-directed delivery of truncated tissue factor to the tumor vasculature. Clin. Cancer Res. 11: 6317-6324, 2005 (*shared senior authorship)

5. Persigehl T, Bieker R, Matuszewski L, Wall A, Kessler T, Kooijman H, Meier N, Ebert W, Berdel WE, Heindel W, Mesters RM, Bremer C: Antiangiogenic tumor treatment: Monitoring with USPIO-enhanced MR imaging in mice. Radiology 244: 449-456, 2007

6. Persigehl T, Matuszewski L, Kessler T, Wall A, Meier N, Ebert W, Berdel WE, Heindel W, Mesters R, Bremer C: Prediction of antiangiogenic treatment efficacy by iron oxide enhanced parametric magnetic resonance imaging. Invest. Radiol. 42: 791-796, 2007

7. Kessler T, Schwöppe C, Liersch R, Schliemann C, Hintelmann H, Bieker R, Berdel WE, Mesters RM: Generation of fusion proteins for selective occlusion of tumor vessels. Current Drug Discovery Technologies 5, 1-8, 2008

8. Bieker R, Kessler T, Schwöppe C, Padro T, Persigehl T, Bremer C, Dreischalück J, Kolkmeyer A, Heindel W, Mesters RM*, Berdel WE*: Infarction of tumor vessels by NGR-peptide directed targeting of tissue factor. Experimental results and first-in-man experience. Blood 113, 5019-5027, 2009 (see also INSIDE BLOOD analysis by Teicher BA pp. 4827-4828)

9. Schwöppe C, Kessler T, Persigehl T, Liersch R, Hintelmann H, Dreischalück J, Ring J, Bremer C, Heindel W, Mesters RM, Berdel WE: Tissue-factor fusion proteins induce occlusion of tumor vessels. Thrombosis Res. 125 (Suppl. 2), S143-S150, 2010

10. Dreischalück J, Schwöppe C, Spieker T, Kessler T, Tiemann K, Liersch R, Schliemann C, Kreuter M, Kolkmeyer A, Hintelmann H, Mesters RM, Berdel WE: Vascular infarction by subcutaneous application of tissue-factor targeted to tumor vessels with NGR-peptides: activity and toxicity profile. Int. J. Oncol. 37, 1389-1397, 2010

11. Von Maltzahn G, Park J-H, Lin KY, Singh N, Schwöppe C, Mesters R, Berdel WE, Ruoslahti E, Sailor MJ, Bhatia SN: Nanoparticles that communicate in vivo

to amplify tumour targeting. Nature Mater. 10: 545-552, 2011 (see also News and Views: Wang Y, Xia Y: Nature Mater. 10: 482-483, 2011)

12. Schwöppe C, Zerbst C, Fröhlich M, Schliemann C, Kessler T, Liersch R, Overkamp L, Holtmeier R, Stypmann J, Dreiling A, König S, Höltke C, Lücke M, Müller-Tidow C, Mesters RM, Berdel WE. Anticancer therapy by tumor vessel infarction with polyethylene glycol conjugated tissue factor. J. Med. Chem. 56. 2337-2347, 2013

13. Persigehl T, Ring J, Bremer C, Heindel W, Holtmeier R, Stypmann J, Claesener M, Hermann S, Schäfers M, Zerbst C, Schliemann C, Mesters RM, Berdel WE, Schwöppe C: Non-invasive monitoring of tumor-vessel infarction by retargeted truncated tissue factor tTF-NGR using multi-modal imaging. Angiogenesis 17: 235-246, 2014

14. Brand C, Dencks S, Schmitz G, Mühlmeister M, Stypmann J, Ross R, Hintelmann H, Schliemann C, Müller-Tidow C, Mesters RM, Berdel WE, Schwöppe C: Low-energy ultrasound treatment improves regional tumor vessel infarction by retargeted tissue factor. J. Ultrasound Med. 34: 1227-1236, 2015

15. Brand C, Fröhlich M, Ring J, Schliemann C, Kessler T, Mantke V, König S, Lücke M, Mesters RM, Berdel WE*, Schwöppe C*: Tumor growth inhibition via occlusion of tumor vasculature induced by N-terminally PEGylated retargeted tissue factor tTF-NGR. Mol. Pharmaceutics 12: 3749-3758, 2015

16. Berdel WE: tTF-NGR, Investigators Brochure (IB) tTF-NGR, attached/on file.

17. Stucke-Ring J, Ronnacker J, Brand C, Höltke C, Schliemann C, Kessler T, Schmidt LH, Harrach S, Mantke V, Hintelmann H, Hartmann W, Wardelmann E, Lenz G, Wünsch B, Müller-Tidow C, Mesters RM, Schwöppe C, Berdel WE: Combinatorial effects of doxorubicin and retargeted tissue factor by intratumoral entrapment of doxorubicin and proapoptotic increase of tumor vascular infarction. Oncotarget 7(50): 82458-82472, 2016

18. Schmidt LH, Brand C, Stucke-Ring J, Schliemann C, Kessler T, Harrach S, Mohr M, Görlich D, Marra A, Hillejan L, Müller-Tidow C, Lenz G, Wardelmann E, Wiewrodt R, Berdel WE*, Schwöppe C*, Hartmann W*: Potential therapeutic impact of CD13 expression in non-small cell lung cancer. PLoS ONE 12(6): e0177146. https://doi.org/10.1371/journal. pone.0177146, 2017

19. Schmidt LH, Stucke-Ring J, Brand C, Schliemann C, Harrach S, Muley T, Herpel E, Kessler T, Mohr M, Görlich D, Kreuter M, Lenz G, Wardelmann E, Thomas M, Berdel WE*, Schwöppe C*, Hartmann W*: CD13 as target for tissue factor induced tumor vascular infarction in small cell lung cancer. Lung Cancer 113: 121-127, 2017

20. Kessler T, Baumeier A, Brand C, Grau M, Angenendt L, Harrach S, Stalmann U, Schmidt LH, Gosheger G, Hardes J, Andreou D, Dreischalück J, Lenz G, Wardelmann E, Mesters RM, Schwöppe C, Berdel WE*, Hartmann W*, Schliemann C*: Aminopeptidase N (CD13): expression, prognostic impact, and use as therapeutic target for tissue factor induced tumor vascular infarction in soft tissue sarcoma. Translational Oncol. 11 (6): 1271-1282, 2018

21. Höink, A, Persigehl T, Kwiecien R, Balthasar M, Mesters R, Berdel WE, Heindel W, Bremer C, Schwöppe C: Gadofosveset-enhanced MRI as simple surrogate parameter for real-time evaluation of the initial tumour vessel infarction by retargeted tissue factor tTF-NGR. Oncol. Letters 17: 270-280, 2019

22. Gerwing M, Herrmann K, Helfen A, Schliemann C, Berdel WE, Eisenblätter M, Wildgruber M: The beginning of the end for conventional RECIST – novel therapies require novel imaging approaches. Nature Rev. Clin. Oncol. doi: 10.1038/s41571-019-0169-5. [Epub ahead of print], 2019

23. "CHMP evaluation" (PDF).

24. Demetri GD, von Mehren M, Jones RL, Hensley ML, Schuetze SM, Staddon A, Milhem M, Elias A, Ganjoo K, Tawbi H, Van Tine BA, Spira A, Dean A, Khokhar NZ, Park YC, Knoblauch RE, Parekh TV, Maki RG, Patel SR. Efficacy and safety of trabectidin or dacarbazine for metastatic liposarcoma or leiomyosarcoma after failure of conventional chemotherapy: results of a phase III randomized multicentre trial. J. Clin. Oncol. 34: 786-793, 2016.

25. De Santis R, Marrari A, Marchetti S, Mussi C, Balzarini L, Lutman FR, Daolio P, Bastoni S, Bertuzzi AF, Quagliuolo V, Santoro A: Efficacy of trabectedin in advanced soft tissue sarcoma: beyond lipo- and leiomyosarcoma. Drug Design, Develop. and Therapy 9: 5785-5791, 2015.

26. Angarita FA, Cannell AJ, Abdul Razak AR, Dickson BC, Blackstein ME: Trabectedin for inoperable or recurrent soft tissue sarcoma in adult patients: a retrospective cohort study. BMC Cancer 16: 30, 2016 (DOI 10.1186/s12885-016-2054-2).

27. Le Cesne A. Cresta S, Maki RG, Blay JY, Verweij J, Poveda A, Casali PG, Balana C, Schöffski P, Grosso F, Lardelli P, Nieto A, Alfaro V, Demetri GD: A retrospective analysis of antitumor activity with trabectedin in translocation-related sarcomas. Eur. J. Cancer 48: 3036-3044, 2012.

28. Sanfilippo R, Dileo P, Blay JY, Constantinidou A, Le Cesne A, Benson C, Vizzini L, Contu M, Bals´di CG, Dei Tos AP, Casali PG: Trabectedin in advanced

synovial sarcomas: a multicenter retrospective study from the European institutions and the Italian rare cancer network. Anti-Cancer Drugs 26: 678-681, 2015.

29. Baruchel S, Pappo A, Krailo M, Baker KS, Wu B, Villaluna D, Lee-Scott M, Adamson PC, Blaney SM: A phase 2 trial of trabectedin in children with recurrent rhabdomyosarcoma, Ewing sarcoma and non-rhabdomyosarcoma soft tissue sarcomas: a report from the children´s oncology group. Eur. J. Cancer 48: 579-585, 2012.

30. Seymour L, Bogaerts J, Perrone A, Ford R, Schwartz LH, Mandrekar S, Lin NU, Litière S, Dancey J, Chen A, Hodi FS, Therasse P, Hoekstra OS, Shankar LK, Wolchok JD, Ballinger M, Caramella C, de Vries EG; RECIST working group. iRECIST: guidelines for response criteria for use in trials testing immunotherapeutics. Lancet Oncol. 18: e143-e152, 2017.

31. Schliemann C, Gerwing M, Heinzow H, Harrach S, Schwöppe C, Wildgruber M, Hansmeier AA, Angenendt L, Berdel AF, Stalmann U, Berning B, Kratz-Albers K, Middelberg-Bisping K, Wiebe S, Albring J, Wilms C, Hartmann W, Wardelmann E, Krähling T, Heindel W, Gerss J, Bormann E, Schmidt H, Lenz G, Kessler T, Mesters RM, Berdel WE: First-in-class CD13-targeted tissue factor tTF-NGR in patients with recurrent or refractory malignant tumors: results of a phase I dose-escalation study. Cancers (Basel) 12 (6), E1488; https://doi.org/10.3390/cancers12061488, 2020.

ATTACHMENTS

Attachment 1 – Summary of product characteristics for trabectedin

Attachment 2 – Fachinformation Yondelis

Attachment 3 – Pharmacy Manual

Attachment 4 – Laboratory Manual

Attachment 5 – KHORANA SCORE

8 INVESTIGATOR AGREEMENT

I have read the preceding protocol "RANDOMIZED STUDY COMPARING TRABECTEDIN (T) VERSUS T PLUS tTF-NGR TO ENTRAP T INSIDE THE TUMOR IN PATIENTS WITH METASTATIC AND/OR REFRACTORY SOFT TISSUE SARCOMA (STS)" and received the following:

- Current version of the Investigator's Brochure (Version 18)

for the respective compound.

I have read this protocol and agree to conduct this study in accordance with all stipulations of the protocol and in accordance with the Declaration of Helsinki.

I will ensure that all persons assisting with the trial are adequately informed about the protocol, any amendments to the protocol, the study treatments, and their trial-related duties and functions. I will maintain a list of appropriate qualified persons to whom I have delegated significant trial-related duties.

I will not enroll the first patient in the study until I have received approval from the appropriate EC, where applicable, and until all legal requirements in my country have been fulfilled.

I will provide a curriculum vitae before the study starts. I agree that these data may be submitted, if necessary, to the relevant authorities.

Signing this protocol I agree to:

- conduct this clinical study in compliance with ICH GCP, with the applicable regulatory requirement(s), and with the study protocol agreed to by the sponsor and given approval/favorable opinion by the EC and competent authority
- comply with procedures for data recording/reporting and
- permit monitoring, auditing, inspection, and EC review and

Investigator

____ ____________

Signature (Place, Date)

Print name:
